# Supplementary material for: Early evolutionary branching across spatial domains predisposes to clonal replacement under chemotherapy in neuroblastoma
Source: Nat Commun. 2024 Oct 18;15:8992. doi: 10.1038/s41467-024-53334-x (PMC11486966; doi:10.1038/s41467-024-53334-x)
Supplement: Supplementary file 1 — Supplementary Information [file 41467_2024_53334_MOESM1_ESM.pdf]

Suppl Fig 1

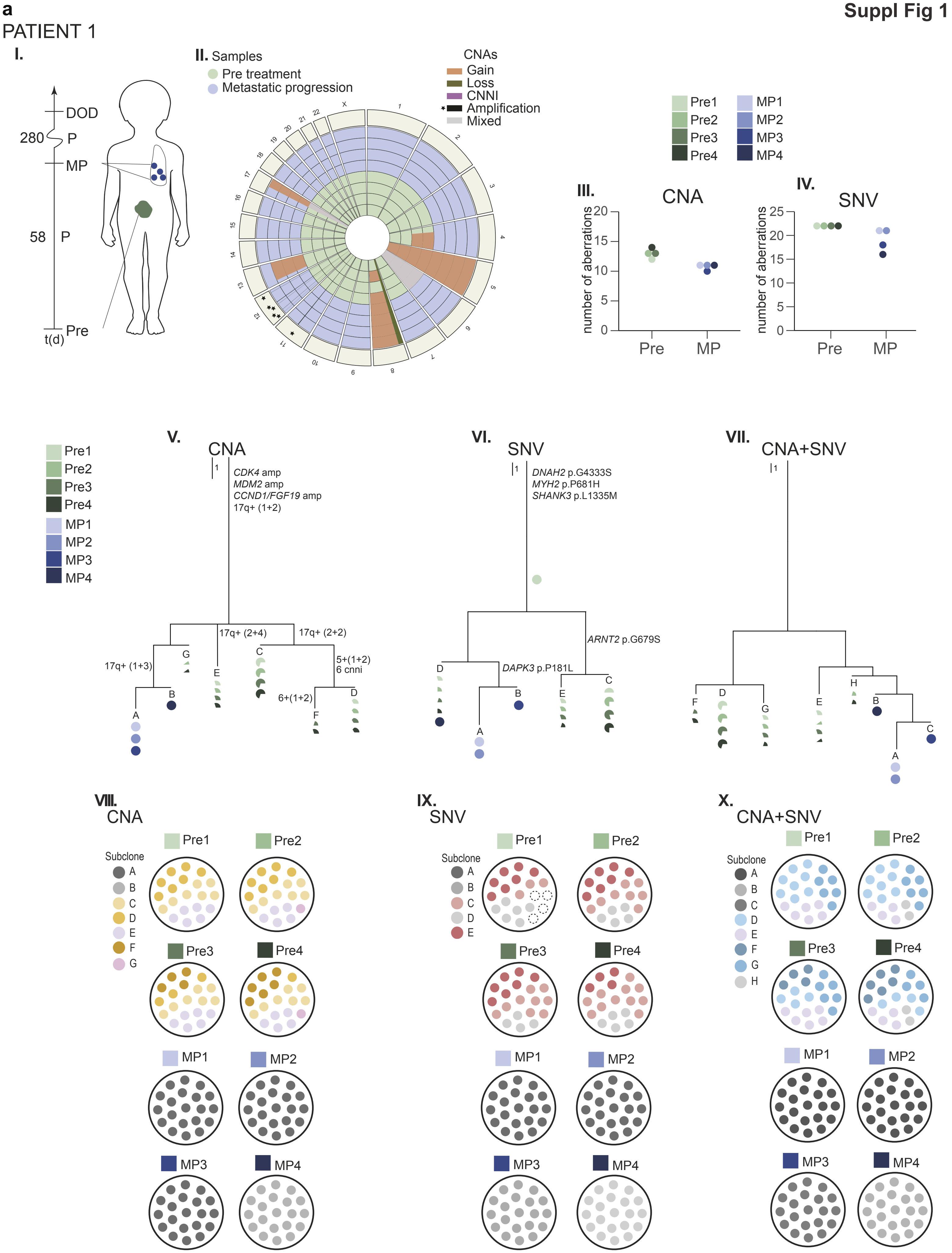
a
PATIENT 1
I.
DOD
280
P
MP
58
P
Pre
t(d)
II. Samples
Pre treatment
Metastatic progression
CNAs
Gain
Loss
CNNI
Amplification
Mixed
Pre1
Pre2
Pre3
Pre4
MP1
MP2
MP3
MP4
III.
CNA
number of aberrations
IV.
SNV
V.
CNA
CDK4 amp
MDM2 amp
CCND1/FGF19 amp
17q+ (1+2)
17q+ (2+4)
17q+ (2+2)
17q+ (1+3)
5+(1+2)
6 cnni
6+(1+2)
VI.
SNV
DNAH2 p.G4333S
MYH2 p.P681H
SHANK3 p.L1335M
ARNT2 p.G679S
DAPK3 p.P181L
VII.
CNA+SNV
VIII.
CNA
Subclone
IX.
SNV
X.
CNA+SNV

Suppl Fig 1

b
PATIENT 2

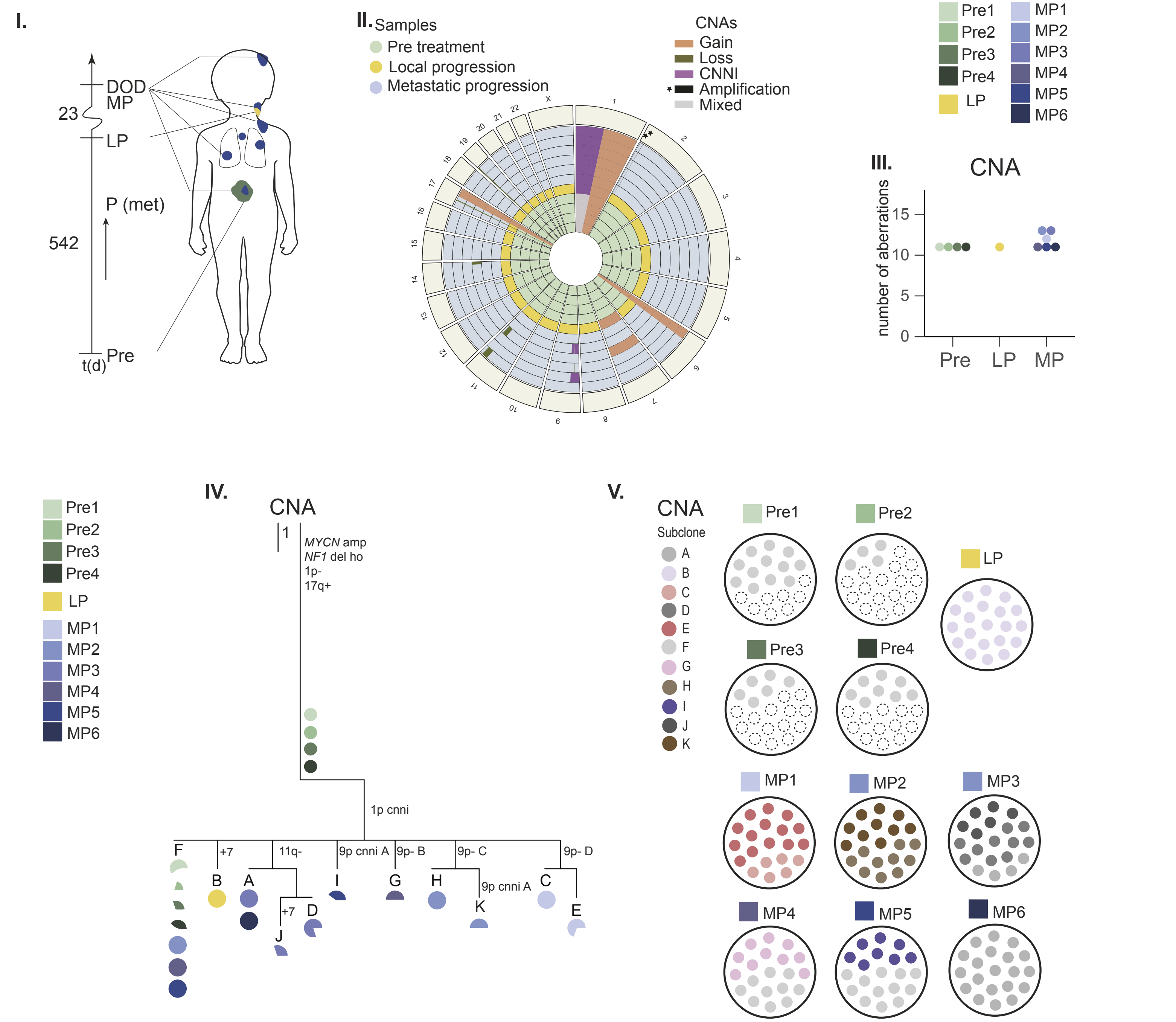

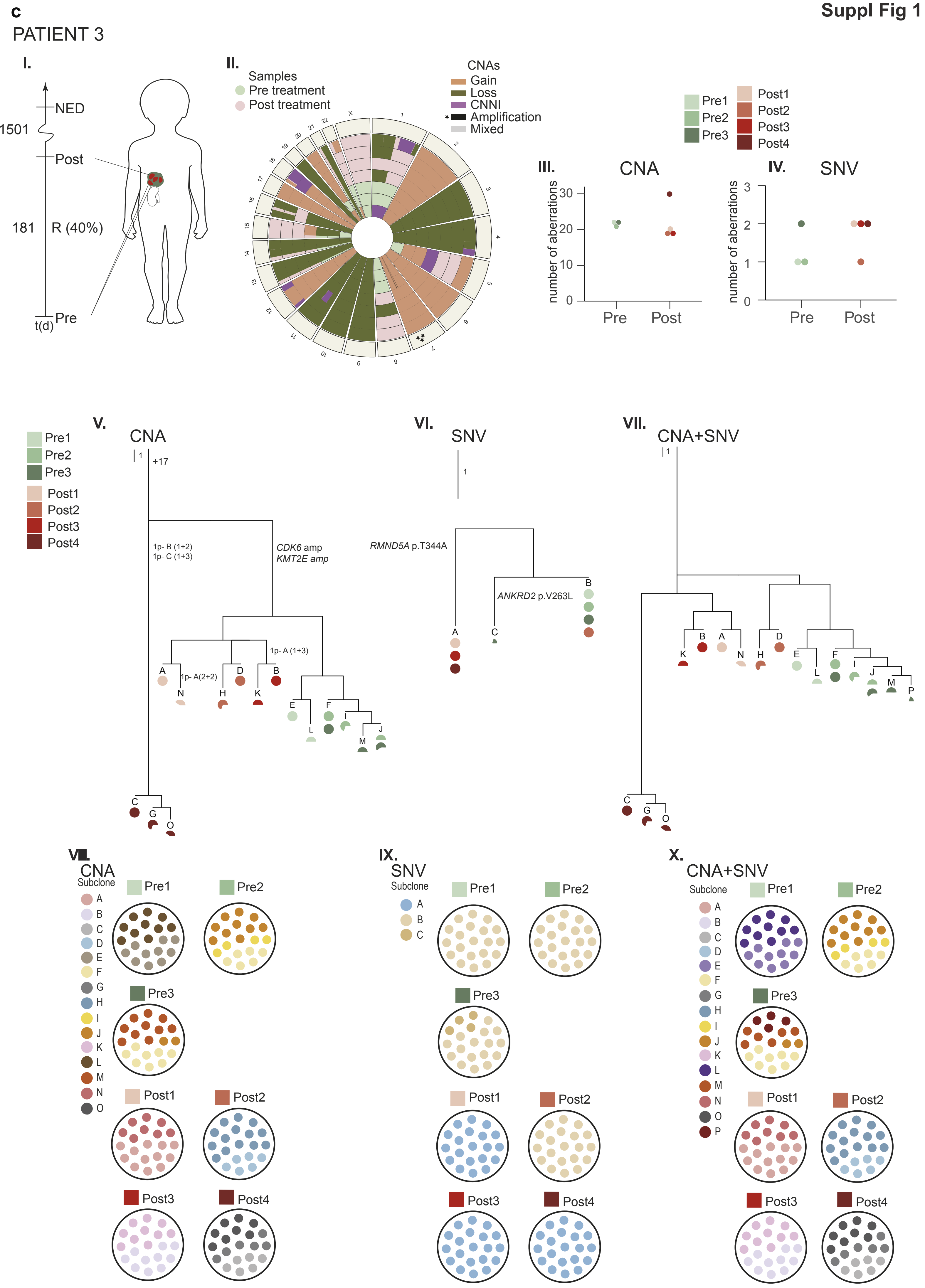
c
PATIENT 3
I.
NED
1501
Post
181
R (40%)
t(d)
Pre
II.
Samples
Pre treatment
Post treatment
CNAs
Gain
Loss
CNNI
Amplification
Mixed
Pre1
Pre2
Pre3
Post1
Post2
Post3
Post4
III.
CNA
number of aberrations
IV.
SNV
V.
CNA
+17
1p- B (1+2)
1p- C (1+3)
CDK6 amp
KMT2E amp
1p- A (1+3)
1p- A(2+2)
VI.
SNV
RMND5A p.T344A
ANKRD2 p.V263L
VII.
CNA+SNV
VIII.
CNA
Subclone
IX.
SNV
Subclone
X.
CNA+SNV
Subclone

Suppl Fig 1

d
PATIENT 4

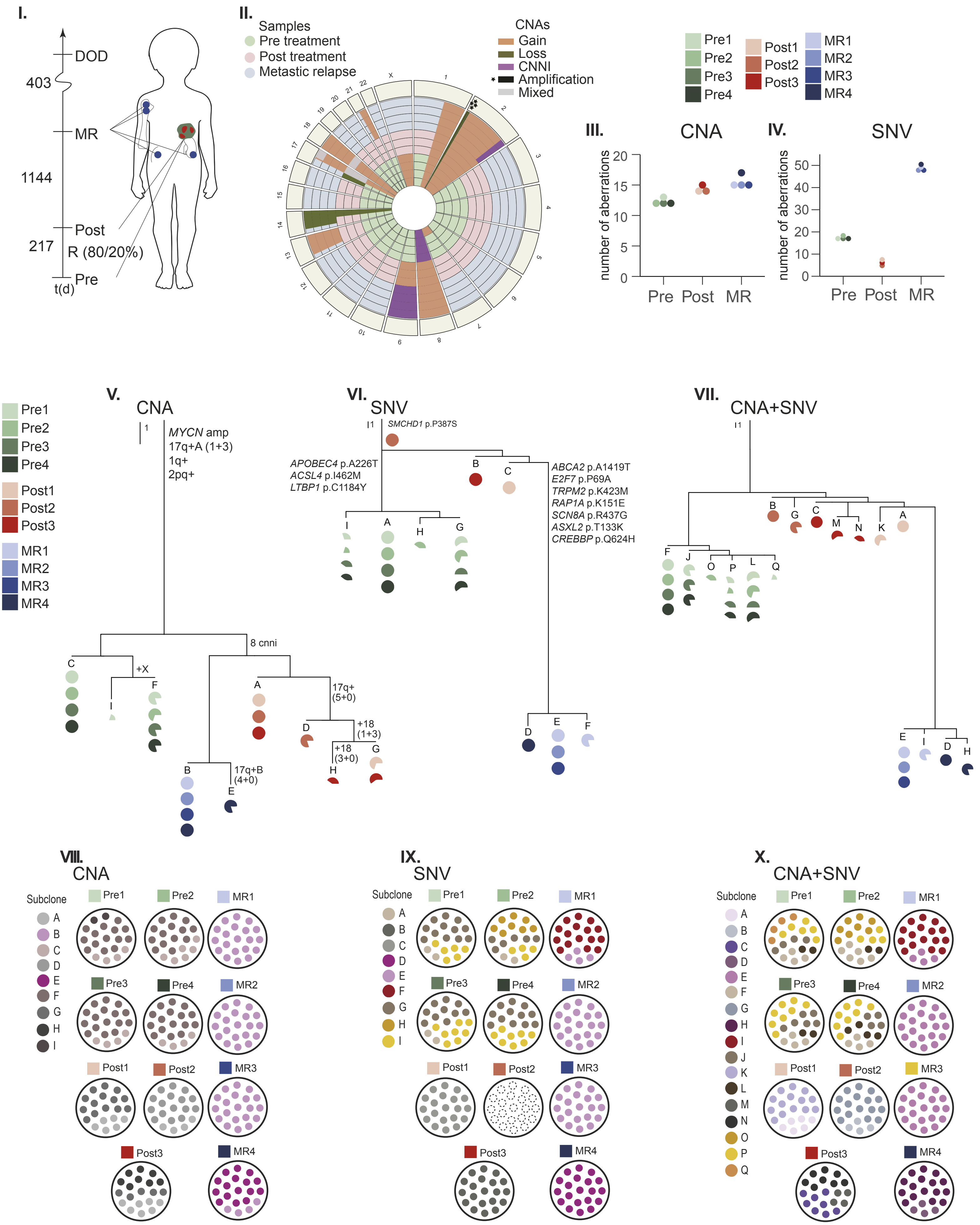

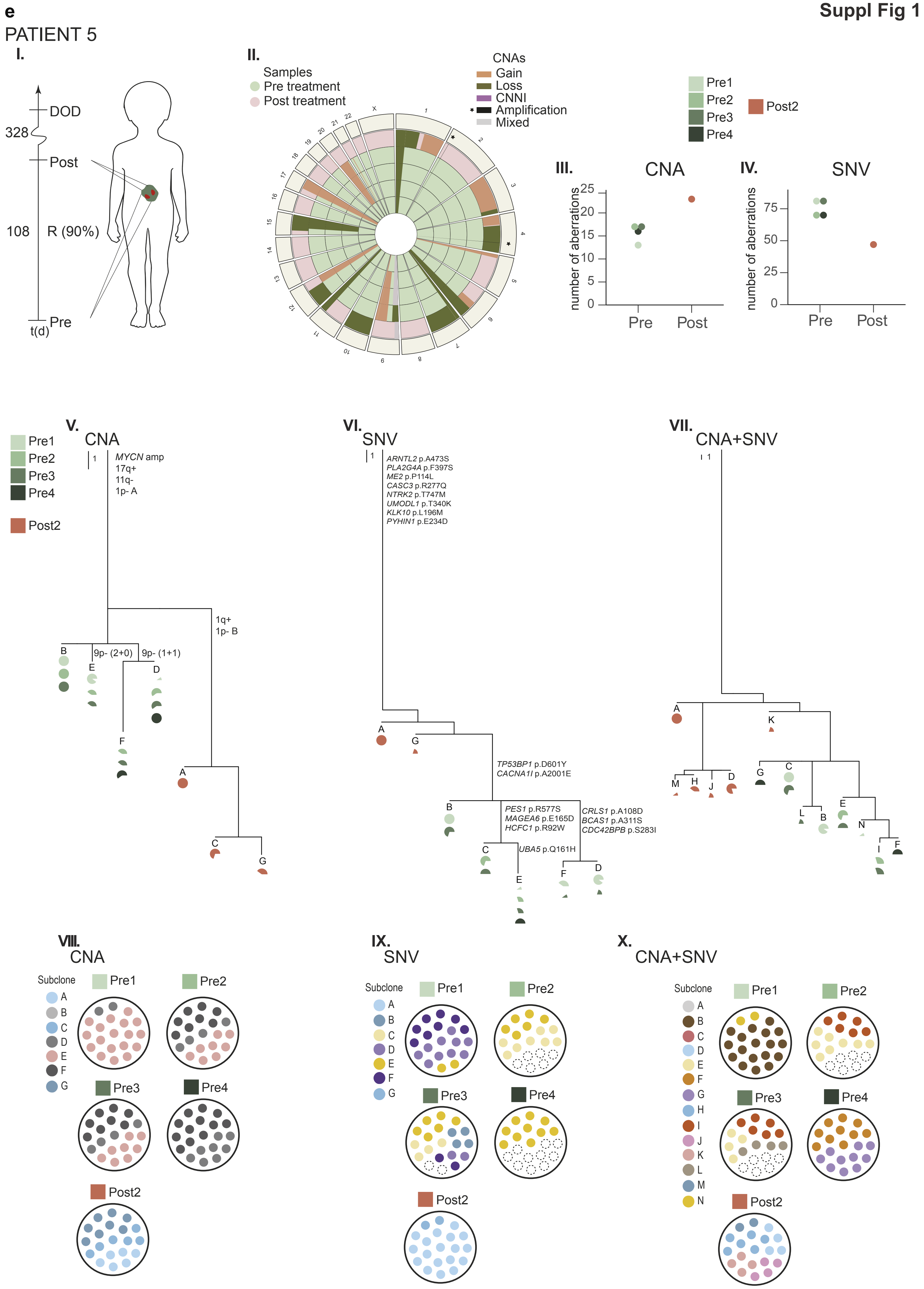
e
PATIENT 5
I.
DOD
328
Post
108
R (90%)
Pre
t(d)
II.
Samples
Pre treatment
Post treatment
CNAs
Gain
Loss
CNNI
Amplification
Mixed
Pre1
Pre2
Pre3
Pre4
Post2
III.
CNA
number of aberrations
Pre
Post
IV.
SNV
V.
CNA
MYCN amp
17q+
11q-
1p- A
1q+
1p- B
9p- (2+0)
9p- (1+1)
VI.
SNV
ARNTL2 p.A473S
PLA2G4A p.F397S
ME2 p.P114L
CASC3 p.R277Q
NTRK2 p.T747M
UMODL1 p.T340K
KLK10 p.L196M
PYHIN1 p.E234D
TP53BP1 p.D601Y
CACNA1I p.A2001E
PES1 p.R577S
MAGEA6 p.E165D
HCFC1 p.R92W
CRLS1 p.A108D
BCAS1 p.A311S
CDC42BPB p.S283I
UBA5 p.Q161H
VII.
CNA+SNV
VIII.
CNA
Subclone
IX.
SNV
X.
CNA+SNV

f

PATIENT 6

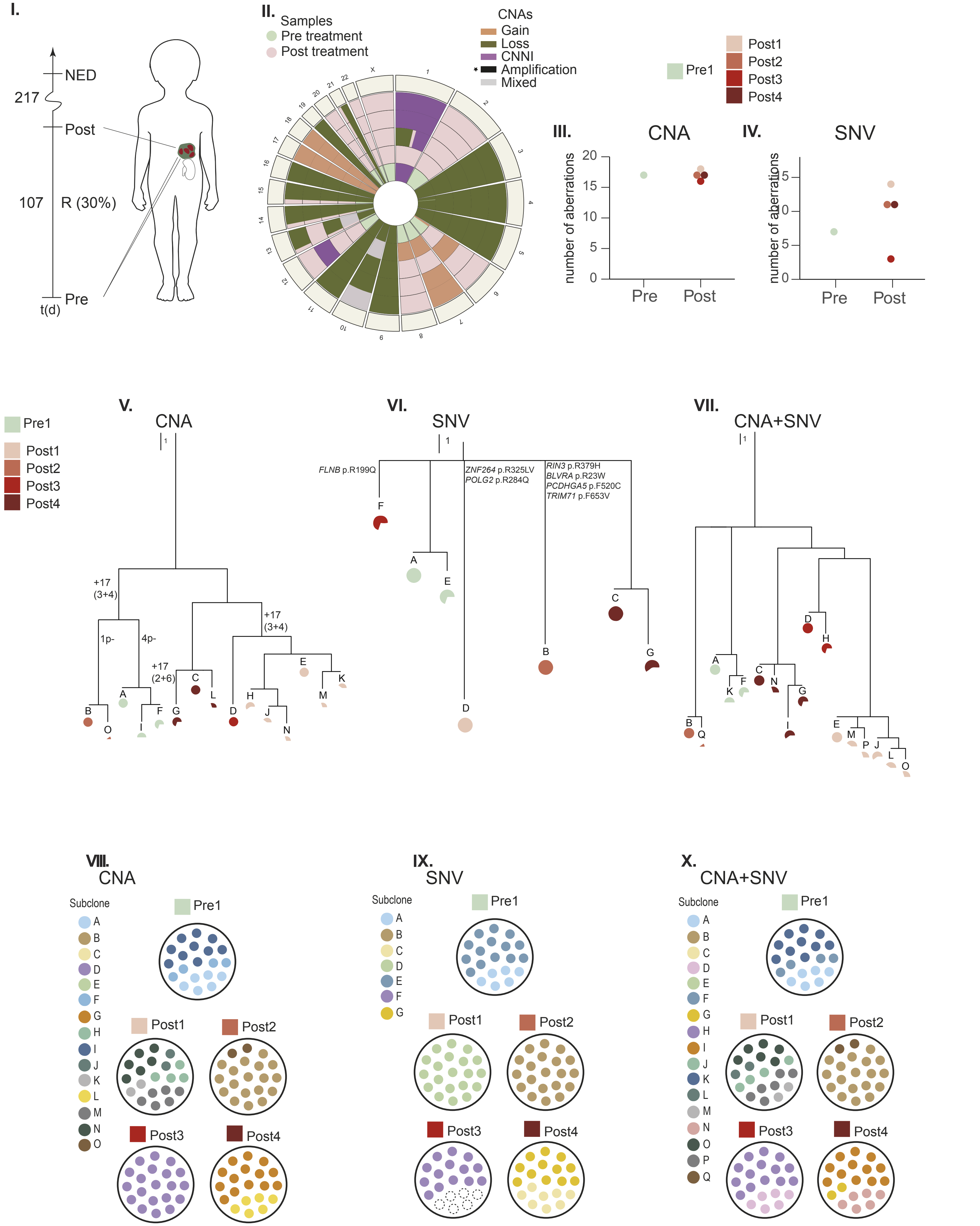
I.
NED
217
Post
107
R (30%)
Pre
t(d)
II.
Samples
Pre treatment
Post treatment
CNAs
Gain
Loss
CNNI
Amplification
Mixed
Pre1
Post1
Post2
Post3
Post4
III.
CNA
number of aberrations
Pre
Post
IV.
SNV
number of aberrations
Pre
Post
V.
CNA
+17 (3+4)
1p-
4p-
+17 (2+6)
+17 (3+4)
VI.
SNV
FLNB p.R199Q
ZNF264 p.R325LV
POLG2 p.R284Q
RIN3 p.R379H
BLVRA p.R23W
PCDHGA5 p.F520C
TRIM71 p.F653V
VII.
CNA+SNV
VIII.
CNA
Subclone
IX.
SNV
Subclone
X.
CNA+SNV
Subclone

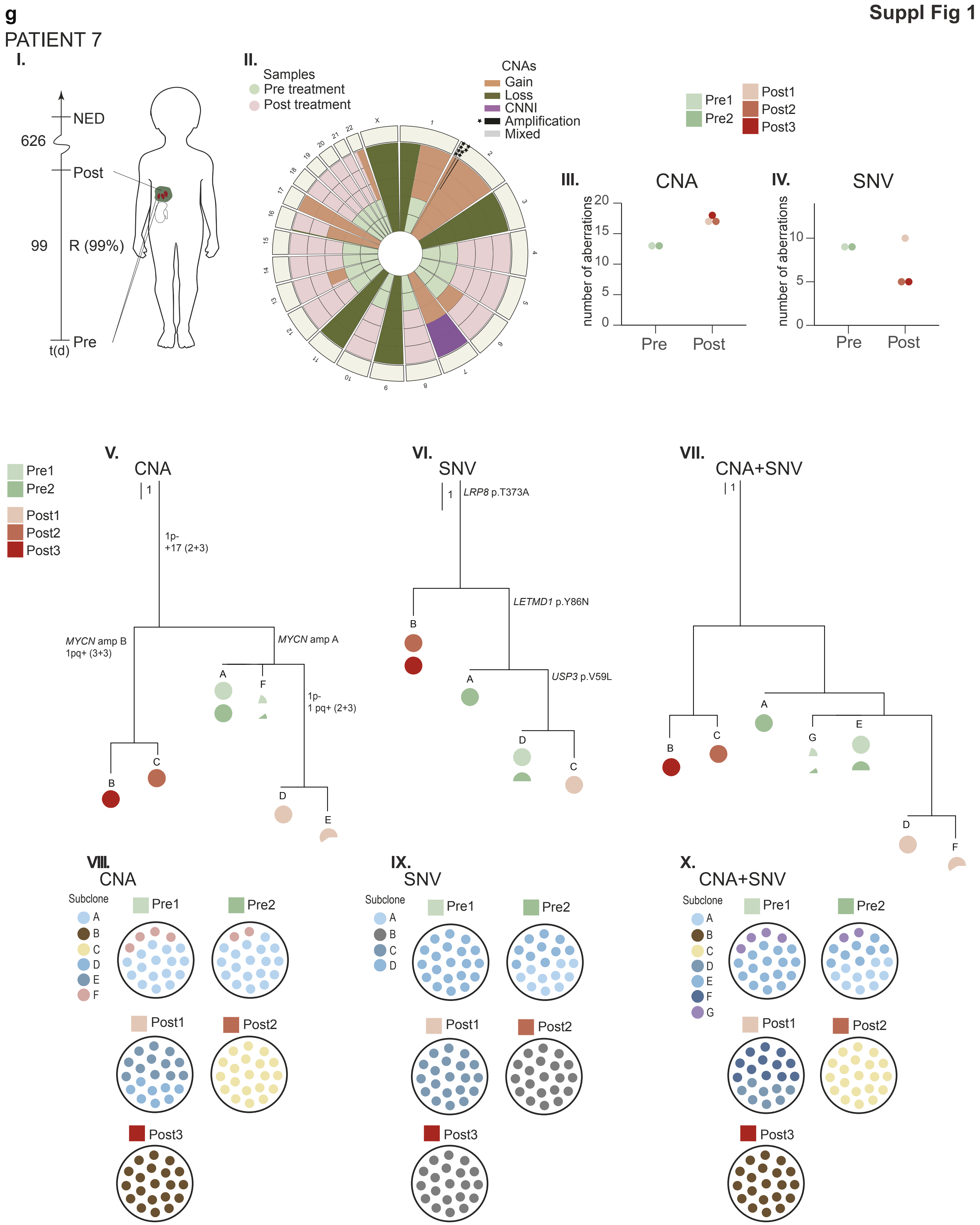
g
PATIENT 7
I.
NED
626
Post
99
R (99%)
t(d)
Pre
II.
Samples
Pre treatment
Post treatment
CNAs
Gain
Loss
CNNI
Amplification
Mixed
Pre1
Pre2
Post1
Post2
Post3
III.
CNA
number of aberrations
Pre
Post
IV.
SNV
number of aberrations
Pre
Post
V.
CNA
1p-
+17 (2+3)
MYCN amp B
1pq+ (3+3)
MYCN amp A
1p-
1 pq+ (2+3)
VI.
SNV
LRP8 p.T373A
LETMD1 p.Y86N
USP3 p.V59L
VII.
CNA+SNV
VIII.
CNA
Subclone
IX.
SNV
Subclone
X.
CNA+SNV
Subclone

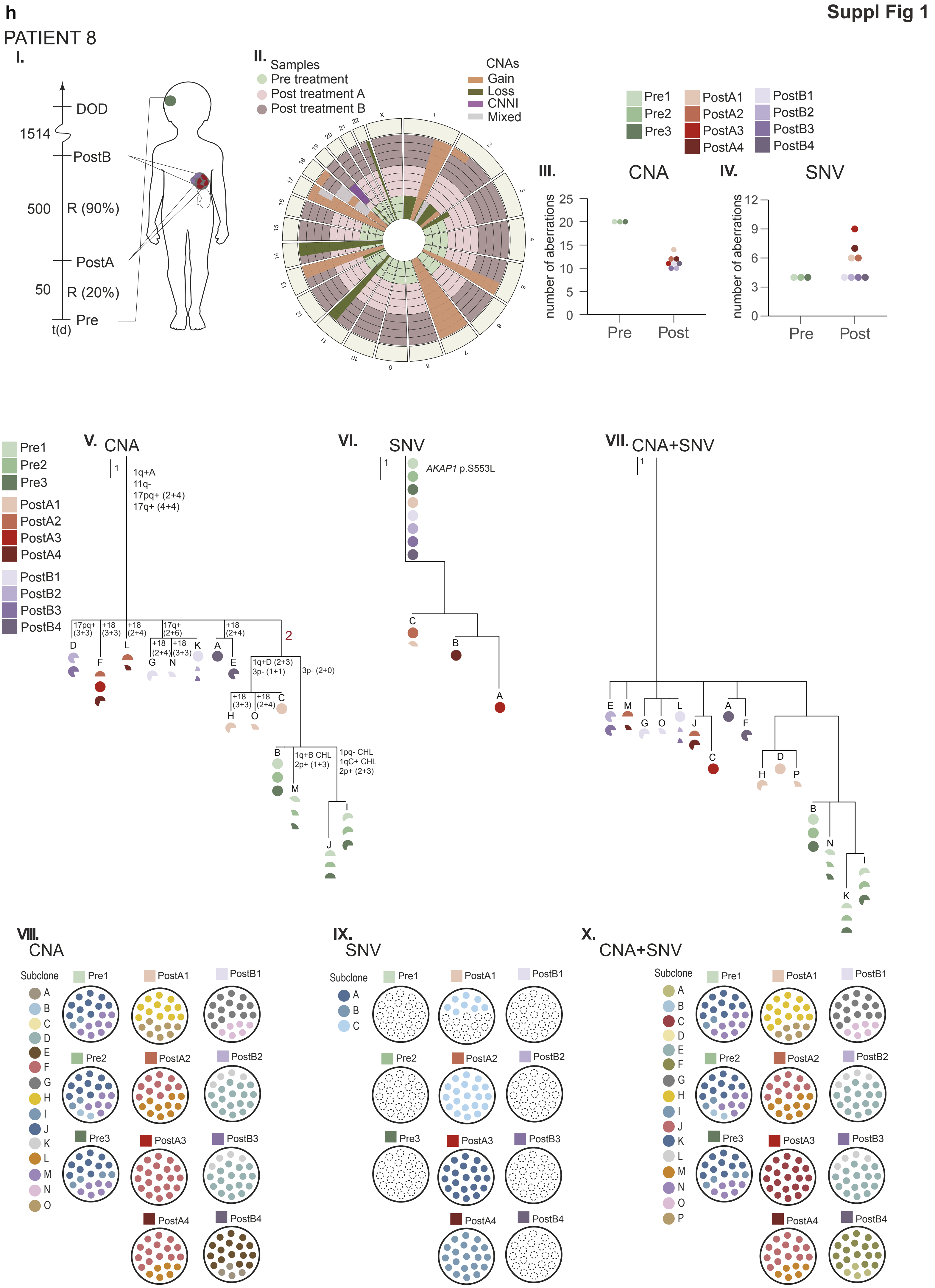
h
PATIENT 8
I.
DOD
1514
PostB
500
R (90%)
PostA
50
R (20%)
Pre
t(d)
II.
Samples
Pre treatment
Post treatment A
Post treatment B
CNAs
Gain
Loss
CNNI
Mixed
Pre1
Pre2
Pre3
PostA1
PostA2
PostA3
PostA4
PostB1
PostB2
PostB3
PostB4
III.
CNA
number of aberrations
Pre
Post
IV.
SNV
V.
CNA
1q+A
11q-
17pq+ (2+4)
17q+ (4+4)
17pq+ (3+3)
+18 (3+3)
+18 (2+4)
17q+ (2+6)
+18 (2+4)
+18 (3+3)
1q+D (2+3)
3p- (1+1)
3p- (2+0)
1q+B CHL
2p+ (1+3)
1pq- CHL
1qC+ CHL
2p+ (2+3)
VI.
SNV
AKAP1 p.S553L
VII.
CNA+SNV
VIII.
CNA
IX.
SNV
X.
CNA+SNV
Subclone

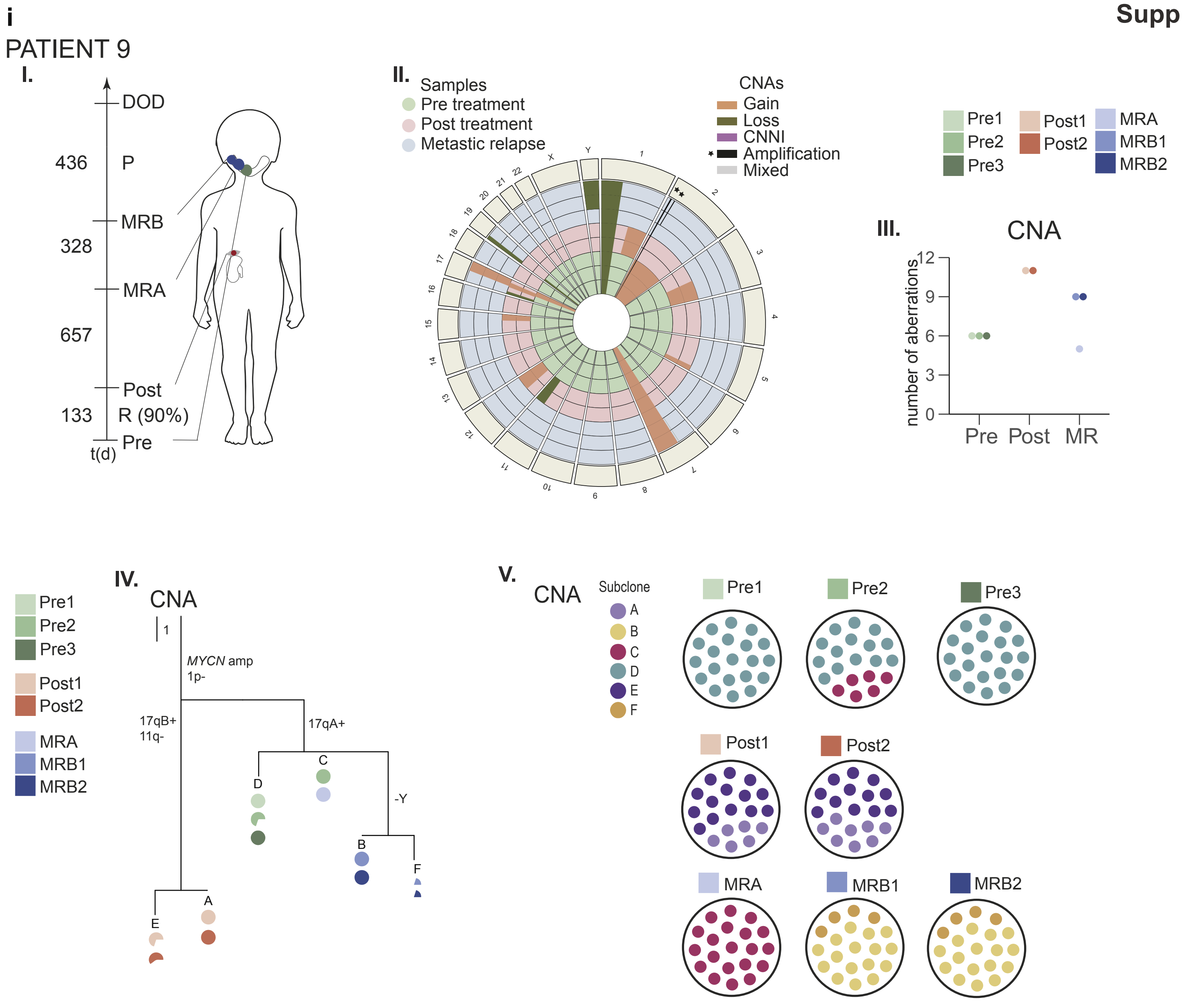
i
PATIENT 9
I.
DOD
436
P
MRB
328
MRA
657
Post
133
R (90%)
Pre
t(d)
II.
Samples
Pre treatment
Post treatment
Metastatic relapse
CNAs
Gain
Loss
CNNI
Amplification
Mixed
Pre1
Pre2
Pre3
Post1
Post2
MRA
MRB1
MRB2
III.
CNA
number of aberrations
12
9
6
3
0
Pre
Post
MR
IV.
CNA
Pre1
Pre2
Pre3
Post1
Post2
MRA
MRB1
MRB2
1
MYCN amp
1p-
17qB+
11q-
17qA+
C
D
-Y
B
F
E
A
V.
CNA
Subclone
A
B
C
D
E
F
Pre1
Pre2
Pre3
Post1
Post2
MRA
MRB1
MRB2

j

PATIENT 10

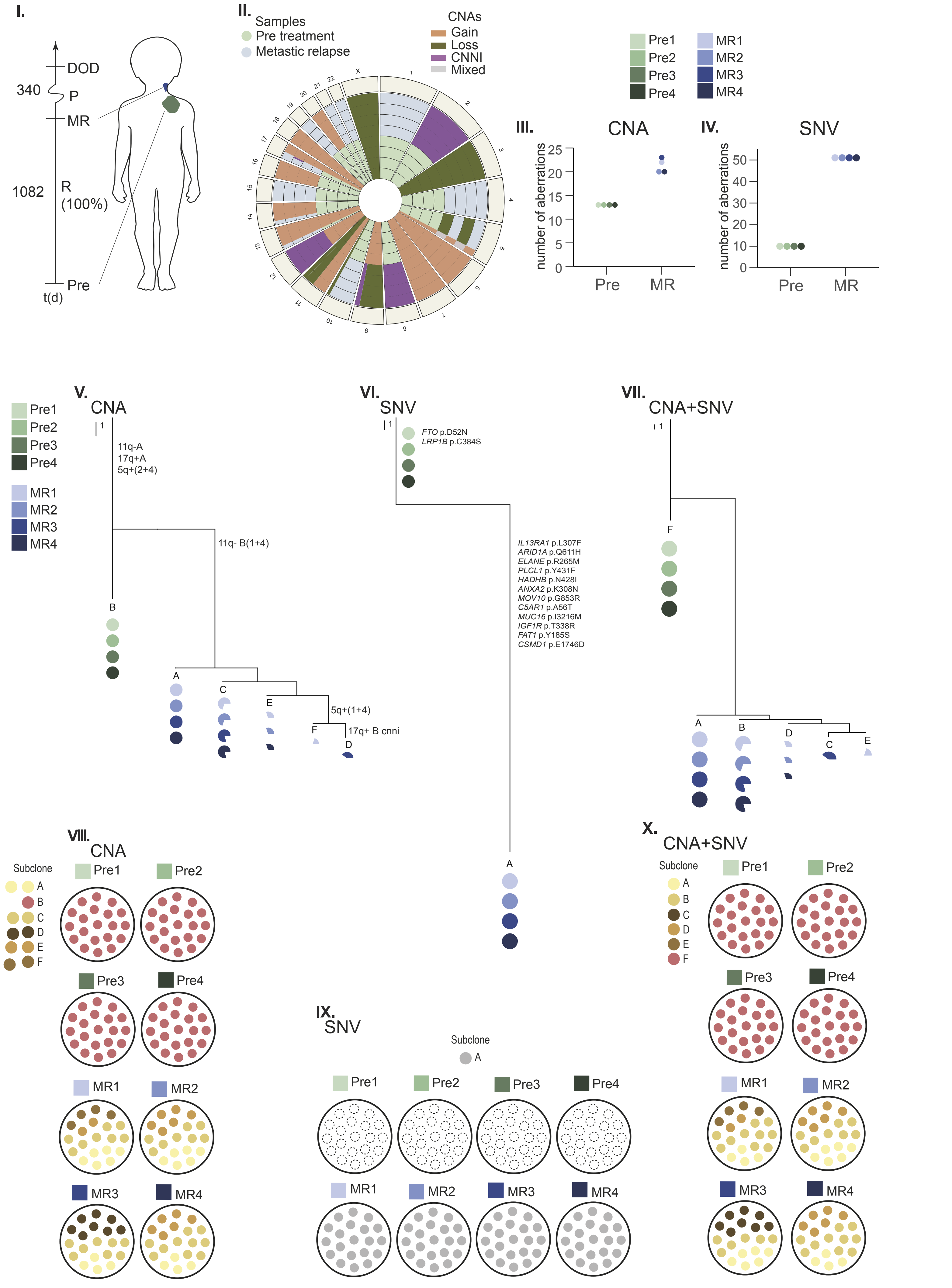
I.
DOD
340
P
MR
1082
R
(100%)
Pre
t(d)
II.
Samples
Pre treatment
Metastic relapse
CNAs
Gain
Loss
CNNI
Mixed
Pre1
Pre2
Pre3
Pre4
MR1
MR2
MR3
MR4
III.
CNA
number of aberrations
Pre
MR
IV.
SNV
number of aberrations
Pre
MR
V.
CNA
11q-A
17q+A
5q+(2+4)
11q- B(1+4)
5q+(1+4)
17q+ B cnni
VI.
SNV
FTO p.D52N
LRP1B p.C384S
IL13RA1 p.L307F
ARID1A p.Q611H
ELANE p.R265M
PLCL1 p.Y431F
HADHB p.N428I
ANXA2 p.K308N
MOV10 p.G853R
C5AR1 p.A56T
MUC16 p.I3216M
IGF1R p.T338R
FAT1 p.Y185S
CSMD1 p.E1746D
VII.
CNA+SNV
VIII.
CNA
Subclone
IX.
SNV
Subclone
X.
CNA+SNV
Subclone

k

PATIENT 11

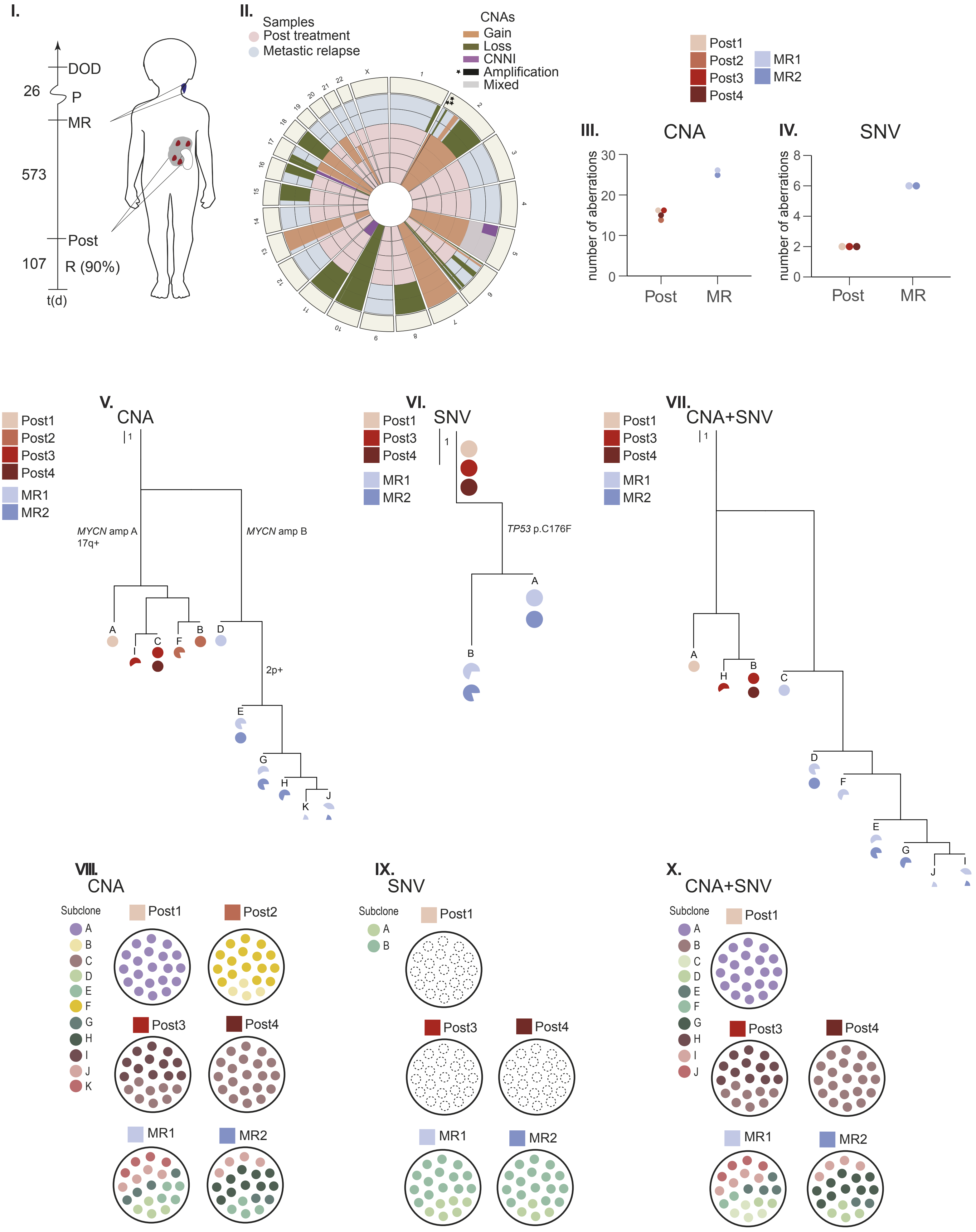
I.
DOD
26
P
MR
573
Post
107
R (90%)
t(d)
II.
Samples
Post treatment
Metastic relapse
CNAs
Gain
Loss
CNNI
Amplification
Mixed
Post1
Post2
Post3
Post4
MR1
MR2
III.
CNA
number of aberrations
Post
MR
IV.
SNV
number of aberrations
Post
MR
V.
CNA
MYCN amp A
17q+
MYCN amp B
2p+
VI.
SNV
TP53 p.C176F
VII.
CNA+SNV
VIII.
CNA
Subclone
IX.
SNV
Subclone
X.
CNA+SNV
Subclone

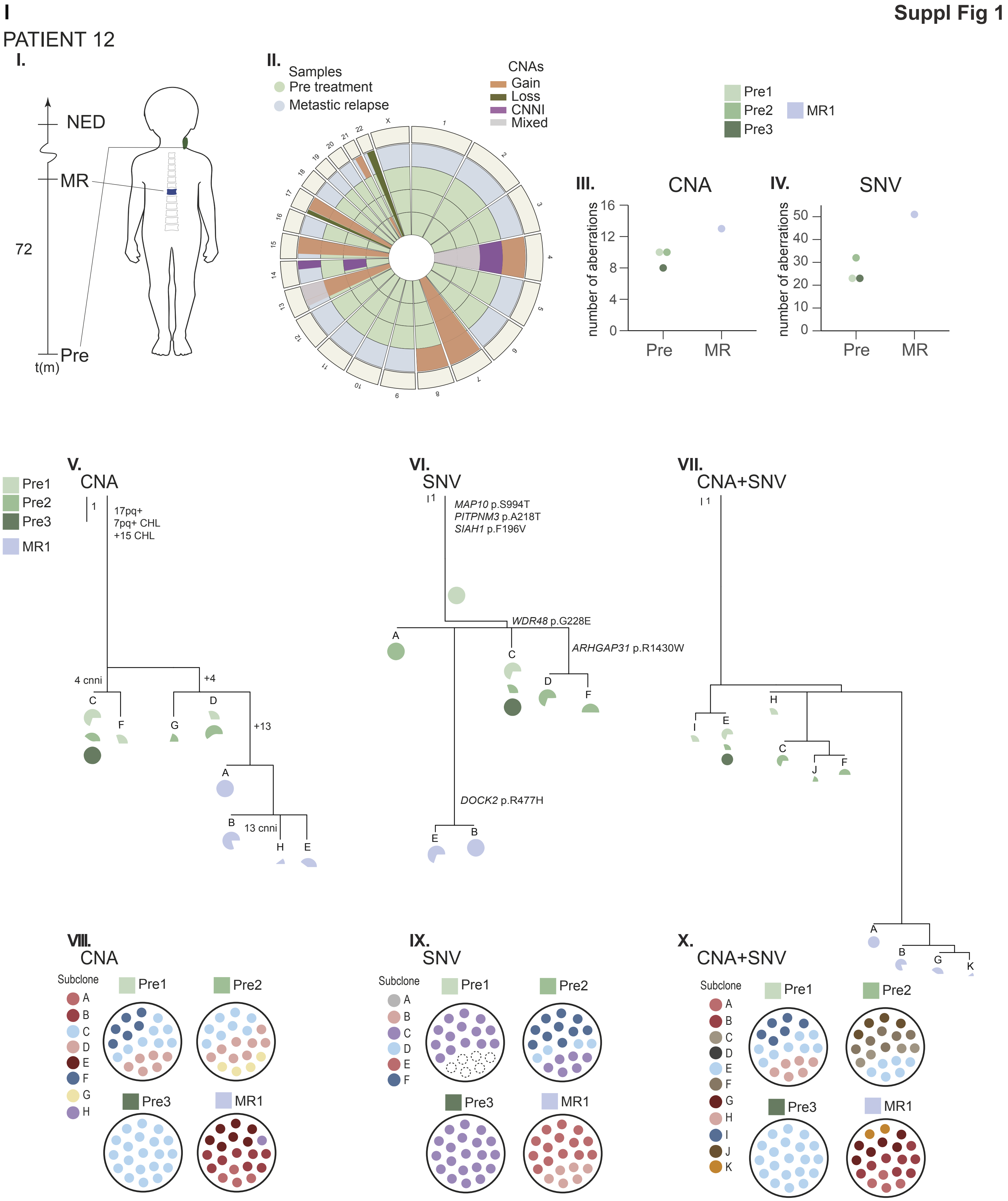

I
Suppl Fig 1
PATIENT 12
I.
NED
MR
72
Pre
t(m)
II.
Samples
Pre treatment
Metastic relapse
CNAs
Gain
Loss
CNNI
Mixed
Pre1
Pre2
Pre3
MR1
III.
CNA
number of aberrations
Pre
MR
IV.
SNV
number of aberrations
Pre
MR
V.
CNA
17pq+
7pq+ CHL
+15 CHL
4 cnni
+4
+13
13 cnni
VI.
SNV
MAP10 p.S994T
PITPNM3 p.A218T
SIAH1 p.F196V
WDR48 p.G228E
ARHGAP31 p.R1430W
DOCK2 p.R477H
VII.
CNA+SNV
VIII.
CNA
Subclone
IX.
SNV
Subclone
X.
CNA+SNV
Subclone

Suppl Fig 1

m

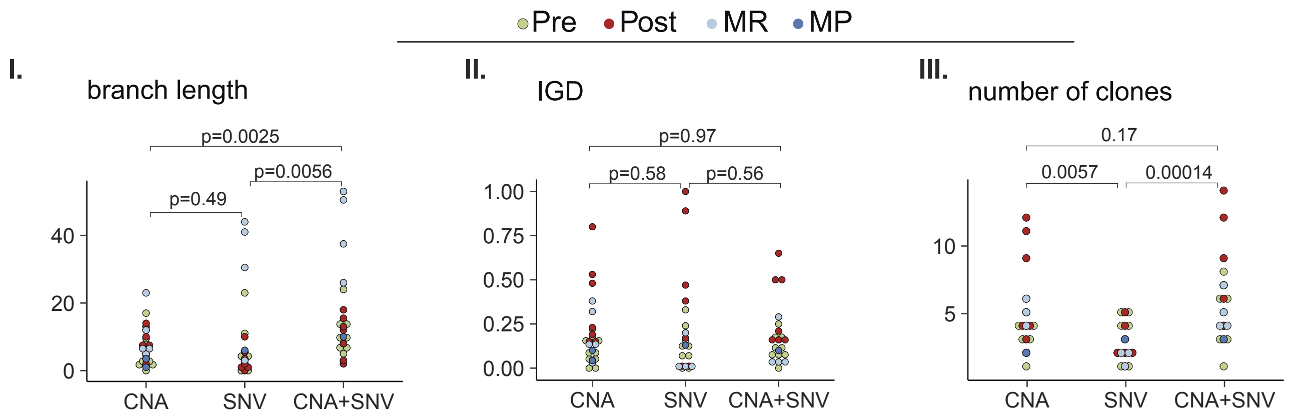

n

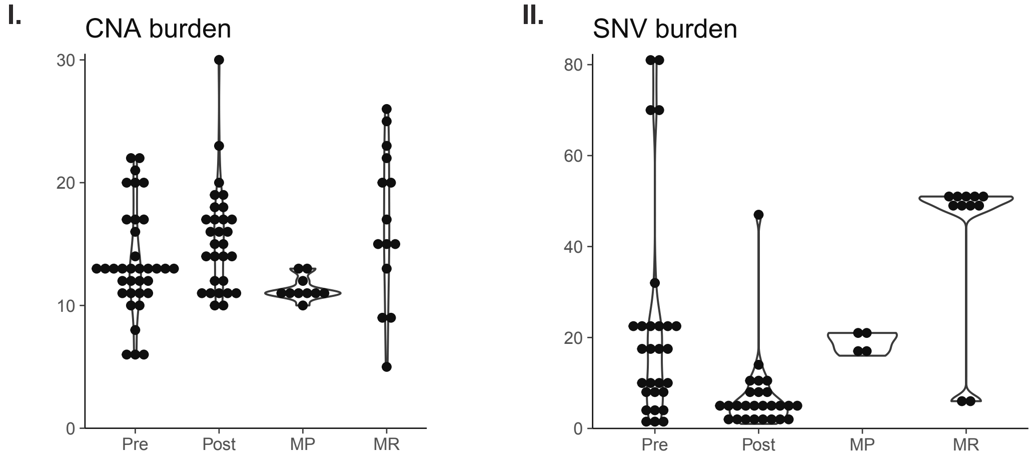

o

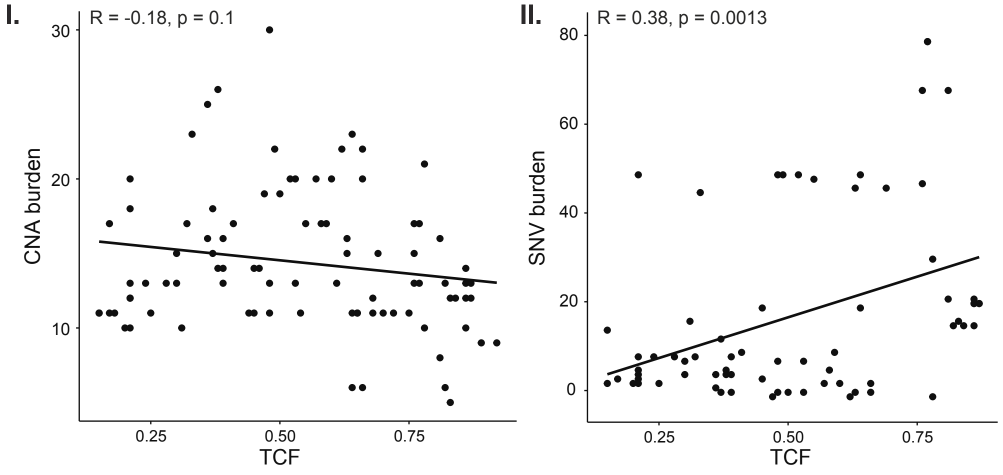

p.

Workflow- From raw data to phylogenetic trees

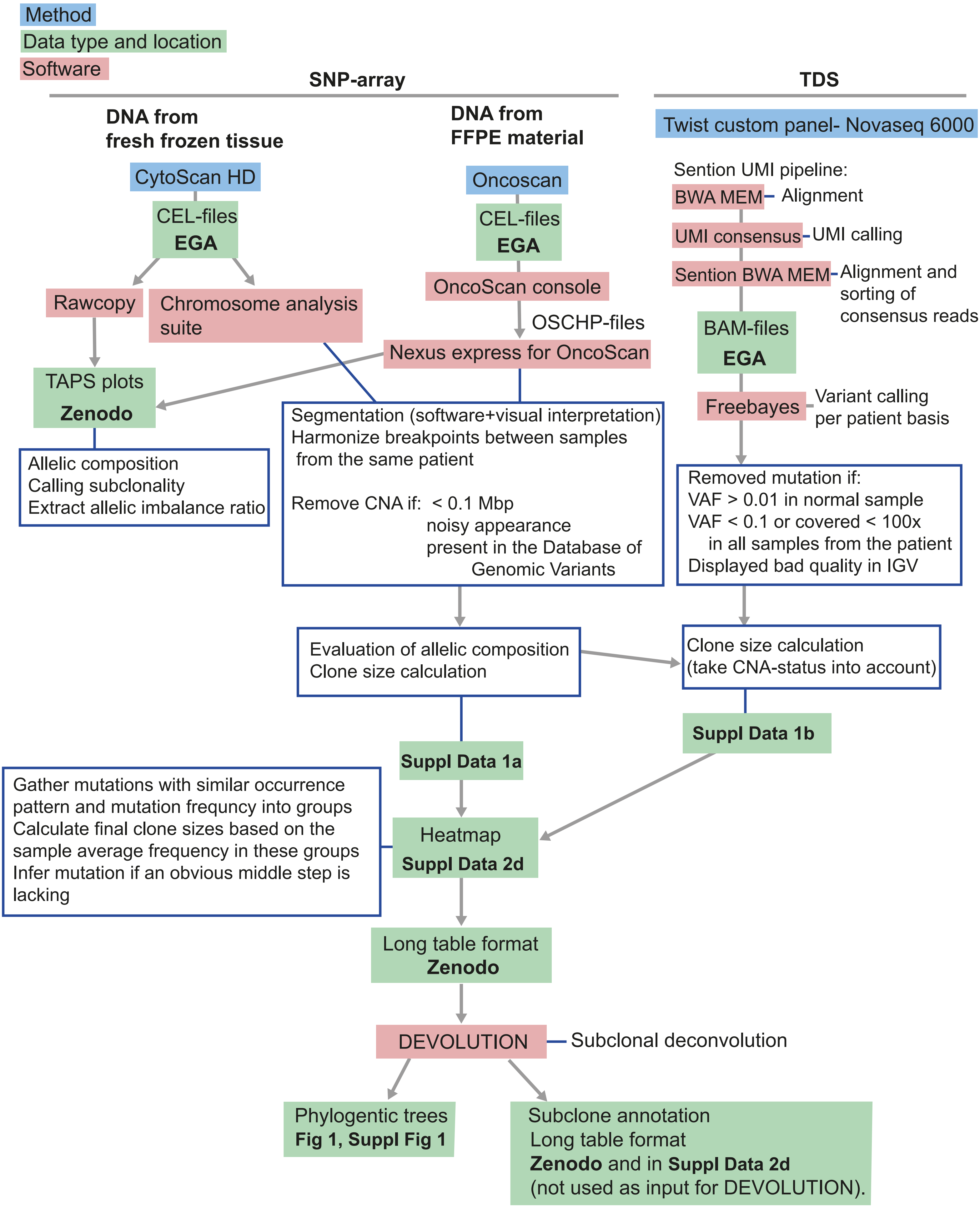

Supplementary Figure 1. Clonal evolution in clinical samples. a-l. Tumors from twelve patients sampled at different time points (Pre= before treatment, Post = post treatment, LP= local progression, MP = metastatic progression, MR = metastatic relapse) were analyzed to investigate the presence of copy number aberrations (CNA). DNA from ten of the patients (all but Patient 2 and Patient 9) were also analyzed with targeted sequencing for single nucleotide variants/InDels (SNV). The data is summarized following the same set up with one patient per page. **I.** Illustration of the location of sampled tumors. The vertical line shows the time course between events or sampling in days (months for patient 12). NED, no evidence of disease; DOD, dead of disease; P, progression; R, regression). **II.** CNAs are illustrated in Circos plots. Each ring corresponds to a sample, and the background color of the ring matches the sample type. All aberrations detected in a sample are indicated with a block of color matching the position of the aberration (see outer ring) and the type of event. The color code for sample type is given to the left of the figure, and the code for the type of aberration is given to the right, where "mixed" indicates an aberration of any type that is present with two different allelic compositions in the same sample. See **Supplementary Data 2a** for details. **III-IV.** Frequency plots showing the number of CNAs (**III**) or the number of SNVs/Indels (**IV**) detected in each sample. The dots are colored according to sample name/type as explained in the color code above the figures. Source data are provided as a Source Data file. **V-VII.** Phylogenetic trees with scale bar (length = one event) to the left of the stem. Aberrations of special interest are highlighted next to the branch leading to the subclone where the event resides. The capital letters at the end of the branches refer to fractions of cells containing the same set of mutations and correspond to information in **Supplementary Data 1d** where a detailed description of all aberrations in the specific fraction can be found. The color of the circles matches the sample where the specific aberrations are present (see code to the left of the trees) and the size of the circle corresponds to the size of the cell fraction with the specific set of aberrations. A full circle next to the stem denotes a sample with no other clonal aberrations in addition to the stem aberrations. Phylogenetic tree based on CNA **(V)**, on targeted sequencing (**VI**) and on the combination of the data (**VII**) respectively. There is no exome sequencing data for Patient 2 or Patient 9, hence the CNV-based trees from these patients are denoted with **IV**. **VIII-X.** Visualization of subclone sizes based on CNA data (**VIII**), on targeted sequencing (**IX**) or on the combination of the two data types (**X**), with the exception for Patient 2 and Patient 9 where clone sizes are based on CNA-data only and shown in figure **V**. Subclone sizes correspond to the fraction of cells containing a specific set of mutations or if followed by a succeeding nested subclone in the phylogenetic tree, calculated by subtracting the size of nested cell populations from the size of the upstream population, figures **V-VII** (**IV**; Patients 2 and 9) and **Supplementary Data 1d**. All cells in a subclone share the same aberrations. Each of the 20 small circles represents a subclone size of 5%. Circles with a dotted line illustrate the size of a subclone with no other aberrations besides the stem events. The names of the subclones (capital letters) match the name of the cell fractions in fig **V-VII** (**IV**; Patients 2 and 9) and the color codes are shown to the left in the figures. **m:I-III.** Comparison of information obtained from phylogenetic trees based on CNA, SNV or the combination (CNA+SNV). The color code to the samples is given above the figures. p-values are calculated with the Mann Whitney U-test (two-sided). Branch length (**I**) is the median value of the number of aberrations detected in each sample from a tumor from one patient at a specific time point. The index of genomic diversity (**II**, IGD) enables the comparison between differences in genetic diversity among subclones. IGD was calculated for each patient using all samples from the same time point. The calculation of IGD is detailed in Methods. The median of the number of all clones (**III**) reflects all tumor cell populations with a unique genomic profile detected in a tumor from a patient of a specific sample type/time point. **n:I-II.** The total number of CNAs and SNVs/indels found per sample at different time points of disease with data included from all patients. **o:I-**

II. Correlation between tumor cell fraction in each sample estimated by largest clone fraction and the total number of detected CNAs and SNVs/indels in each sample. Associations were calculated with the Spearman's correlation test. Source data are provided as a Source Data file (m-o). **p.** Workflow- from raw data to phylogenetic trees. The methods used to analyze the samples are highlighted by blue boxes, input and output files and their location (bold type) by green boxes and softwares by pink boxes. Different methods were used for SNP-array analyses dependent on if the DNA was extracted from fresh frozen tissue or from formalin fixed paraffin embedded (FFPE) material. For details regarding methods and clone size calculations for SNP-array data, see the paragraphs “Copy number analyses by SNP array”, “Clone size estimations based on SNP array data” and “Clonal deconvolution based on CNAs detected by SNP array in clinical samples” in Methods. Details regarding the targeted deep sequencing (TDS) analyses can be found in the Methods sections “Whole exome sequencing and targeted sequencing of clinical samples” and “Calculation of clone sizes based on integration of variant allele frequencies from exome sequencing with copy number from SNP-array data”.

a

Suppl Fig 2

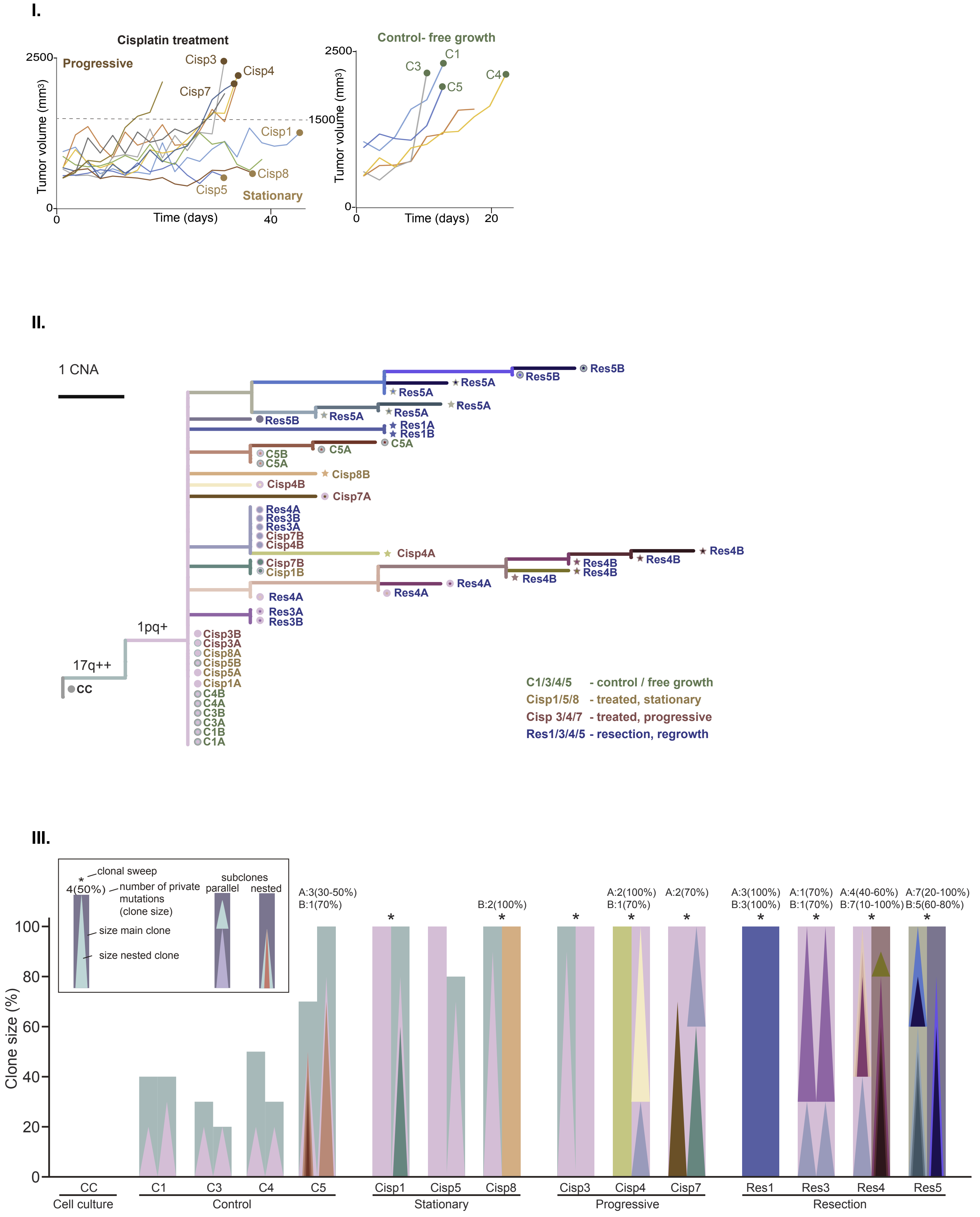

I.
Cisplatin treatment
Progressive
Stationary
Cisp3
Cisp4
Cisp7
Cisp1
Cisp8
Cisp5
Tumor volume (mm³)
Time (days)
2500
1500
0
40
Control- free growth
C1
C3
C4
C5
20
II.
1 CNA
17q++
1pq+
CC
Res5B
Res5A
Res1A
Res1B
C5A
C5B
Cisp8B
Cisp4B
Cisp7A
Res4A
Res3B
Res3A
Cisp7B
Cisp4A
Cisp1B
Res4B
Cisp3B
Cisp3A
Cisp8A
Cisp5B
Cisp5A
Cisp1A
C4B
C4A
C3B
C3A
C1B
C1A
C1/3/4/5 - control / free growth
Cisp1/5/8 - treated, stationary
Cisp 3/4/7 - treated, progressive
Res1/3/4/5 - resection, regrowth
III.
clonal sweep
4(50%)
number of private mutations (clone size)
size main clone
size nested clone
subclones
parallel
nested
A:3(30-50%)
B:1(70%)
B:2(100%)
A:2(100%)
B:1(70%)
A:2(70%)
A:3(100%)
B:3(100%)
A:1(70%)
B:1(70%)
A:4(40-60%)
B:7(10-100%)
A:7(20-100%)
B:5(60-80%)
Clone size (%)
100
80
60
40
20
0
CC
C1
C3
C4
C5
Cisp1
Cisp5
Cisp8
Cisp3
Cisp4
Cisp7
Res1
Res3
Res4
Res5
Cell culture
Control
Stationary
Progressive
Resection

b **Suppl Fig 2**

I.

Color	Group	Location	Type	Shared/Private
	Stem	chr1:1-42924386	Loss	Shared
	Stem	chr2:16020018-17286545	Amp	Shared
	Stem	chr3:4317405-4671835	Loss	Shared
	Stem	chr5:1-46389339	Gain	Shared
	Stem	chr7:146661233-146854133	Loss	Shared
	Stem	chr9:9569860-9683321	Loss	Shared
	Stem	chr11:16844923-16949730	Loss	Shared
	Stem	chr17:54900876-81195210 (1+2)	Gain	Shared
	Stem	chr18:52982062-53191438	Loss	Shared
	α1	chr17:54900876-81195210 (1+3)	Gain	Shared
	α2	chr1:42956395-249224684	Gain	Shared
	α3	chr3:116572755-116791095	Loss	Shared
	α4	chr7:1-58025873	Gain	Shared
	β1	chr12:99231771-99447915	Loss	Private
	β2	chr1:42928433-81628896	Gain	Private
	β3	chr11:102099494-134938470	Loss	Private
	γ1	chr3:116560639-116964135	Loss	Private
	γ1	chr7:77779469-78096262	Loss	Private
	δ1	chr10:56478149-56584348	Loss	Private
	ε1	chr2:80569497-80720855	Loss	Private
	ε1	chr6:161885594-161989970	Loss	Private
	ζ1	chr7:77891737-78023516	Loss	Private
	ζ1	chr7:146600077-146932753	Loss	Private
	η1	chr10:56478223-56741385	Loss	Private
	θ1	chr10:1-38371899	Gain	Private
	θ2	chr17:54900876-81195210 (2+4)	Gain	Private
	θ2	chr18:49829759-50066114	Loss	Private
	θ3	chr17:54900876-81195210 (2+3)	Gain	Private
	θ4	chr17:1-54872134	Gain	Private
	θ5	chr14:20511672-107285437	Loss	Private
	θ6	chr2:212549262-212948126	Loss	Private
	ι1	chr17:1-54872134	Cnni	Private
	κ1	chr2:141890203-142199636	Loss	Private
	κ1	chr2:178657434-178882111	Loss	Private
	κ1	chr3:116852401-116953146	Loss	Private
	λ1	chr10:57021557-57531698	Loss	Private
	λ2	chr2:142367703-142791913	Loss	Private
	λ3	chr10:56532320-56823236	Loss	Private
	λ4	chr4:91628726-91992971	Loss	Private
	λ5	chr9:27974327-28210573	Loss	Private
	λ5	chr12:99294860-99461623	Loss	Private
	λ6	chr2:142615492-142717571	Loss	Private
	λ7	chr20:1-62915555	Gain	Private

II.

Symbol	Main clone	Subclone	Symbol	Main clone	Subclone
	Stem			Stem, α1, α2, γ1	
	Stem	α1		Stem, α1, α2, ζ1	
	Stem	α1, α2		Stem, α1, α2, κ1	
	Stem	α1, α2, β1		Stem, α1, α2, θ1, θ2, θ3	
	Stem	α1, α2, β1, β2		Stem, α1, α2, θ1, θ2, θ3	θ4
	Stem	α1, α2, β1, β2, β3		Stem, α1, α2, θ1, θ2, θ3	θ4, θ5
	Stem, α1	α2		Stem, α1, α2, θ1, θ2, θ3	θ4, θ5, θ6
	Stem, α1	α2, β1		Stem, α1, α2, θ1, θ2, θ3	ι1
	Stem, α1	α2, α3		Stem, α1, α2, λ1	
	Stem, α1, α2			Stem, α1, α2, λ1	λ2
	Stem, α1, α2	α3		Stem, α1, α2, λ1	λ2, λ3
	Stem, α1, α2	α4		Stem, α1, α2, λ1	λ2, λ3, λ4
	Stem, α1, α2	δ1		Stem, α1, α2, λ1	λ5
	Stem, α1, α2	η1		Stem, α1, α2, λ1	λ5, λ7
	Stem, α1, α2	ε1	*	Stem, α2 (-α1)	
	Stem, α1, α2	θ1	*	Stem, α2 (-α1)	α1, λ1, λ5, λ6
	Stem, α1, α2	θ1, θ2	*	Stem, α2 (-α1)	α1, λ1, λ5, λ6, λ7
	Stem, α1, α2	θ1, θ2, θ4			

III.

CC

CC
- CC

Control

C1A
- C1A
- C1A-sα1
- C1A-sα1α2

C1B
- C1B
- C1B-sα1
- C1B-sα1α2

C3A
- C3A
- C3A-sα1
- C3A-sα1α2

C3B
- C3B
- C3B-sα1α2

C4A
- C4A
- C4A-sα1
- C4A-sα1α2

C4B
- C4B
- C4B-sα1
- C4B-sα1α2

C5A
- C5A
- C5A-sα1
- C5A-sα1α2β1
- C5A-sα1α2β1β2
- C5A-sα1α2β1β2β3

C5B
- C5B
- C5B-sα2
- C5B-sα2β1

Stationary

Cisp1A
- Cisp1A

Cisp1B
- Cisp1B
- Cisp1B-sα2
- Cisp1B-sα2α3

Cisp5A
- Cisp5A

Cisp5B
- Cisp5B
- Cisp5B-sα1
- Cisp5B-sα1α2

Cisp8A
- Cisp8A
- Cisp8A-sα2

Cisp8B
- Cisp8B

Progressive

Cisp3A
- Cisp3A
- Cisp3A-sα2

Cisp3B
- Cisp3B

Cisp4A
- (symbol only)

Cisp4B
- Cisp4B-sα4
- Cisp4B-sδ1

Cisp7A
- Cisp7A
- Cisp7A-sε1

Cisp7B
- Cisp7B-sα3
- Cisp7B-sα4

Resection

Res1A
- Res1A

Res1B
- Res1B

Res3A
- Res3A-sα4
- Res3A-sη1

Res3B
- Res3B-sα4
- Res3B-sη1

Res4A
- Res4A-sα4
- Res4A-sθ1
- Res4A-sθ1θ2
- Res4A-sθ1θ2θ4

Res4B
- Res4B
- Res4B-sθ4
- Res4B-sθ4θ5
- Res4B-sθ4θ5θ6
- Res4B-sι1

Res5A
- Res5A-sλ2
- Res5A-sλ2λ3
- Res5A-sλ2λ3λ4
- Res5A-sλ5
- Res5A-sλ5λ7

Res5B
- Res5B
- Res5B-sα1λ1λ5λ6
- Res5B-sα1λ1λ5λ6λ7

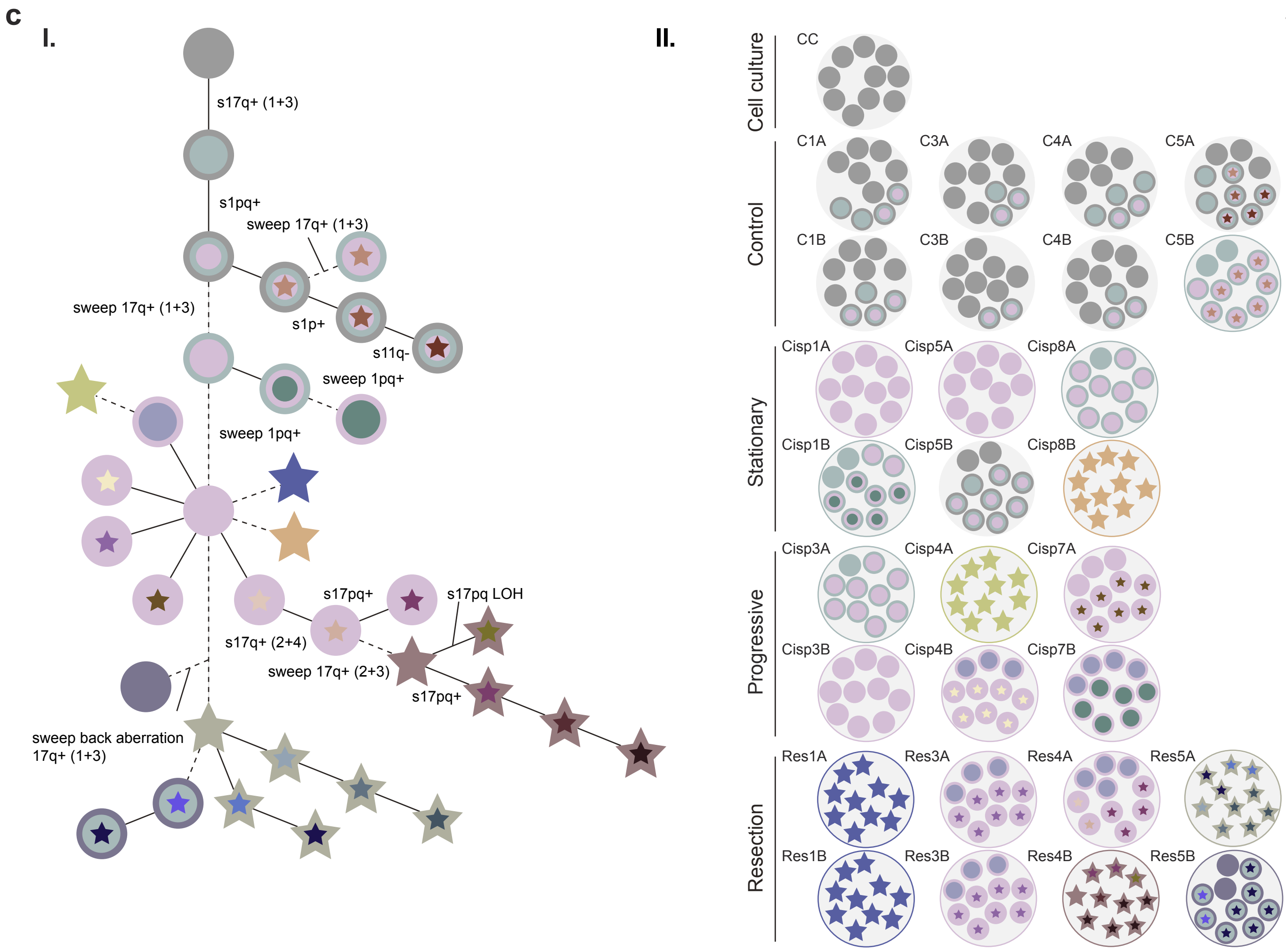
c
I.
s17q+ (1+3)
s1pq+
sweep 17q+ (1+3)
sweep 17q+ (1+3)
s1p+
s11q-
sweep 1pq+
sweep 1pq+
s17pq+
s17pq LOH
s17q+ (2+4)
sweep 17q+ (2+3)
s17pq+
sweep back aberration
17q+ (1+3)
II.
Cell culture
CC
Control
C1A
C3A
C4A
C5A
C1B
C3B
C4B
C5B
Stationary
Cisp1A
Cisp5A
Cisp8A
Cisp1B
Cisp5B
Cisp8B
Progressive
Cisp3A
Cisp4A
Cisp7A
Cisp3B
Cisp4B
Cisp7B
Resection
Res1A
Res3A
Res4A
Res5A
Res1B
Res3B
Res4B
Res5B

d

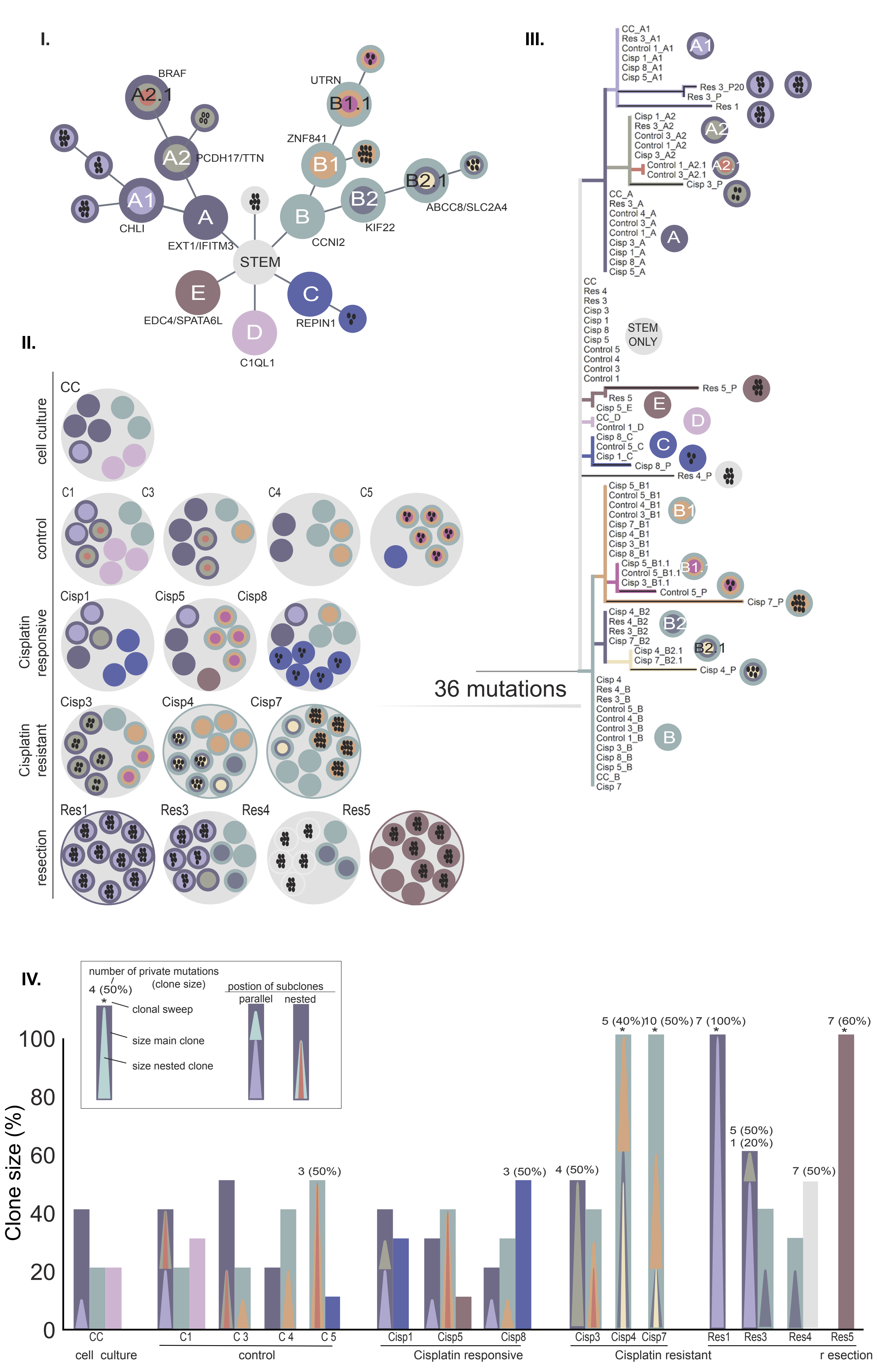
I.
BRAF
A2.1
UTRN
B1.1
ZNF841
A2
PCDH17/TTN
B1
B2.1
A1
CHLI
A
EXT1/IFITM3
B
B2
KIF22
ABCC8/SLC2A4
CCNI2
STEM
E
C
EDC4/SPATA6L
REPIN1
D
C1QL1
II.
cell culture
CC
control
C1
C3
C4
C5
Cisplatin responsive
Cisp1
Cisp5
Cisp8
Cisplatin resistant
Cisp3
Cisp4
Cisp7
resection
Res1
Res3
Res4
Res5
III.
CC_A1
Res 3_A1
Control 1_A1
Cisp 1_A1
Cisp 8_A1
Cisp 5_A1
Res 3_P20
Res 3_P
Res 1
Cisp 1_A2
Res 3_A2
Control 3_A2
Control 1_A2
Cisp 3_A2
Control 1_A2.1
Control 3_A2.1
Cisp 3_P
CC_A
Res 3_A
Control 4_A
Control 3_A
Control 1_A
Cisp 3_A
Cisp 1_A
Cisp 8_A
Cisp 5_A
CC
Res 4
Res 3
Cisp 3
Cisp 1
Cisp 8
Cisp 5
Control 5
Control 4
Control 3
Control 1
STEM ONLY
Res 5_P
Res 5
Cisp 5_E
CC_D
Control 1_D
Cisp 8_C
Control 5_C
Cisp 1_C
Cisp 8_P
Res 4_P
Cisp 5_B1
Control 5_B1
Control 4_B1
Control 3_B1
Cisp 7_B1
Cisp 4_B1
Cisp 3_B1
Cisp 8_B1
Cisp 5_B1.1
Control 5_B1.1
Cisp 3_B1.1
Control 5_P
Cisp 7_P
Cisp 4_B2
Res 4_B2
Res 3_B2
Cisp 7_B2
Cisp 4_B2.1
Cisp 7_B2.1
Cisp 4_P
36 mutations
Cisp 4
Res 4_B
Res 3_B
Control 5_B
Control 4_B
Control 3_B
Control 1_B
Cisp 3_B
Cisp 8_B
Cisp 5_B
CC_B
Cisp 7
IV.
number of private mutations (clone size)
4 (50%)
clonal sweep
size main clone
size nested clone
postion of subclones
parallel
nested
Clone size (%)
100
80
60
40
20
0
3 (50%)
3 (50%)
4 (50%)
5 (40%)
10 (50%)
7 (100%)
5 (50%)
1 (20%)
7 (50%)
7 (60%)
CC
C1
C 3
C 4
C 5
Cisp1
Cisp5
Cisp8
Cisp3
Cisp4
Cisp7
Res1
Res3
Res4
Res5
cell culture
control
Cisplatin responsive
Cisplatin resistant
r esection

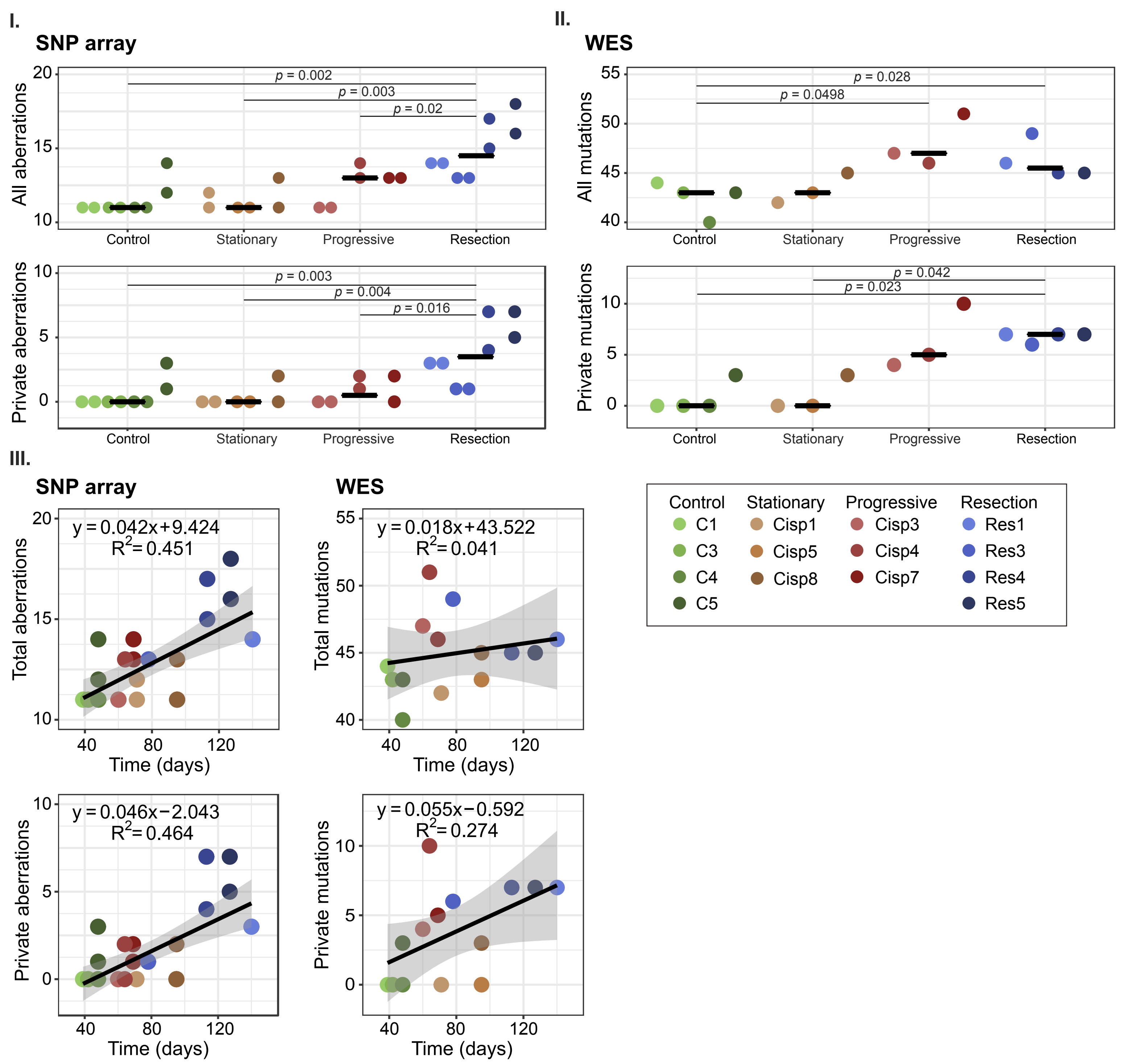
e
I.
SNP array
All aberrations
p = 0.002
p = 0.003
p = 0.02
Private aberrations
p = 0.003
p = 0.004
p = 0.016
Control
Stationary
Progressive
Resection
II.
WES
All mutations
p = 0.028
p = 0.0498
Private mutations
p = 0.042
p = 0.023
III.
SNP array
Total aberrations
y = 0.042x+9.424
R2 = 0.451
Private aberrations
y = 0.046x−2.043
R2 = 0.464
WES
Total mutations
y = 0.018x+43.522
R2 = 0.041
Private mutations
y = 0.055x−0.592
R2 = 0.274
Time (days)
Control
C1
C3
C4
C5
Stationary
Cisp1
Cisp5
Cisp8
Progressive
Cisp3
Cisp4
Cisp7
Resection
Res1
Res3
Res4
Res5

f

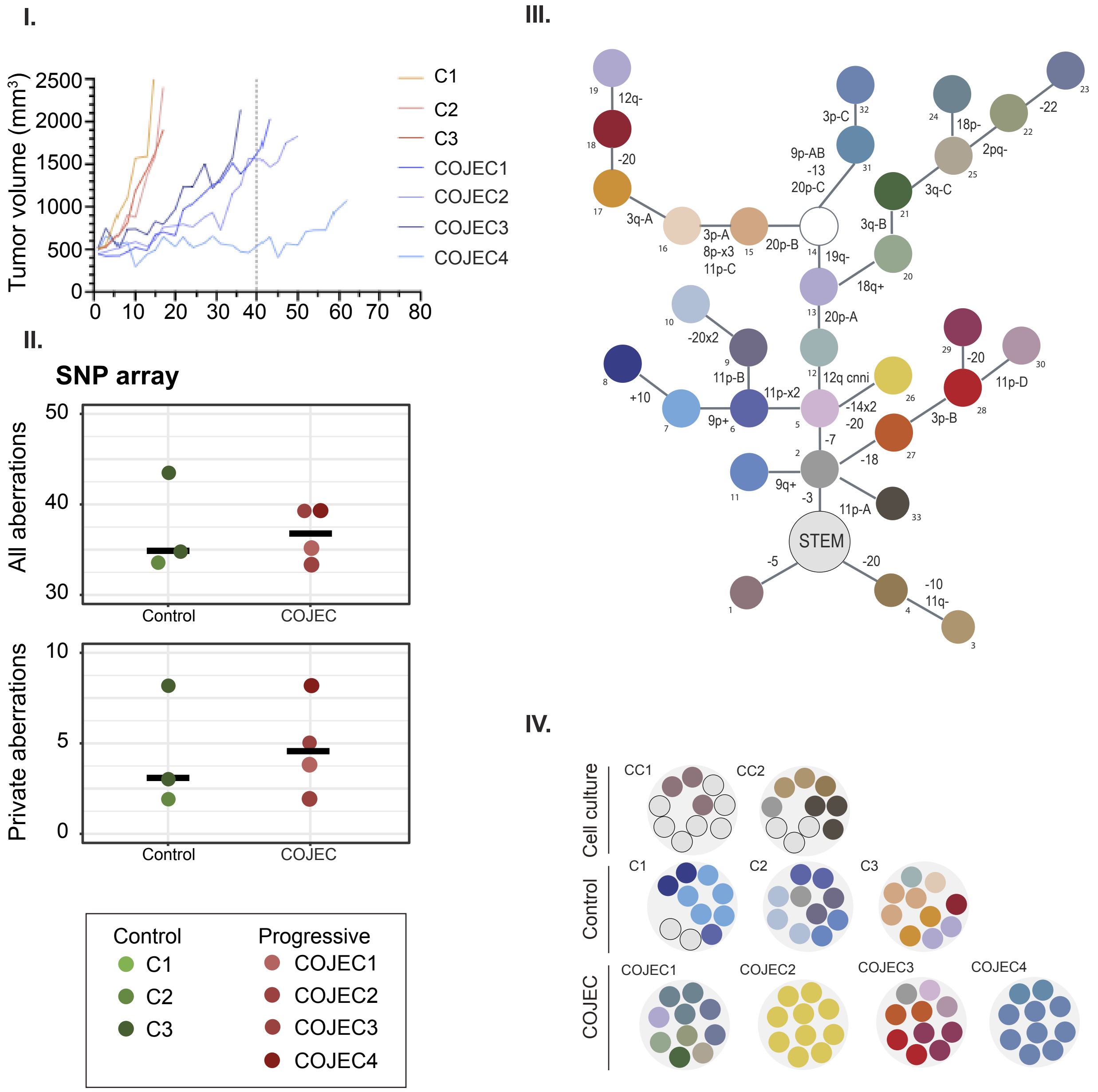

I.
Tumor volume (mm³)
2500
2000
1500
1000
500
0
0 10 20 30 40 50 60 70 80
C1
C2
C3
COJEC1
COJEC2
COJEC3
COJEC4
II.
SNP array
All aberrations
50
40
30
Control
COJEC
Private aberrations
10
5
0
Control
COJEC
Control
C1
C2
C3
Progressive
COJEC1
COJEC2
COJEC3
COJEC4
III.
12q-
-20
3q-A
3p-A
8p-x3
11p-C
20p-B
9p-AB
-13
20p-C
3p-C
19q-
18q+
20p-A
3q-B
3q-C
18p-
2pq-
-22
-20x2
11p-B
+10
9p+
11p-x2
12q cnni
-14x2
-20
-7
-18
3p-B
-20
11p-D
9q+
-3
11p-A
STEM
-5
-20
-10
11q-
IV.
Cell culture
CC1
CC2
Control
C1
C2
C3
COJEC
COJEC1
COJEC2
COJEC3
COJEC4

g

Suppl Fig 2

Color	Group	Location	Type	Shared/private
	Stem	1p22.1-p22.1	Loss (2+0)	Shared
	Stem	1p36.33-p34.2	Loss (2+0)	Shared
	Stem	2p25.3-p23.3	Gain (4+1)	Shared
	Stem	2p24.3-p24.3	Gain (Amp)	Shared
	Stem	2p24.3-p24.3v1	Gain (Amp)	Shared
	Stem	2p23.3-q37.3	Loss (2+1)	Shared
	Stem	3p26.3-q29	Loss (3+1)	Shared
	Stem	4q31.1-q31.1	Gain (Amp)	Shared
	Stem	4q31.1-q31.1v1	Gain (Amp)	Shared
	Stem	4p16.3-q35.2	Loss (2+1)	Shared
	Stem	5p15.33-q35.3	Loss (2+1)	Shared
	Stem	6p25.3-q27	Loss (2+1)	Shared
	Stem	7p21.1-p21.1	Loss (1+0)	Shared
	Stem	7p22.3-q36.3	Loss (3+1)	Shared
	Stem	8p11.22-p11.22	Loss (2+0)	Shared
	Stem	8p23.3-q24.3	Loss (2+1)	Shared
	Stem	9p24.3-p13.3	Loss (2+0)	Shared
	Stem	10p15.3-q26.3	Loss (2+1)	Shared
	Stem	11p15.5-q25	Loss (2+1)	Shared
	Stem	12p13.33-q24.33	Loss (2+1)	Shared
	Stem	13q11-q34	Loss (2+1)	Shared
	Stem	14q11.2-q32.33	Loss (2+1)	Shared
	Stem	15q11.2-q26.3	Loss (2+0)	Shared
	Stem	16p13.3-q24.3	Loss (2+1)	Shared
	Stem	17q21.31-q25.3	Gain (4+1)	Shared
	Stem	17p13.3-q21.31	Loss (2+1)	Shared
	Stem	19p12-p12	Loss (2+0)	Shared
	Stem	19p13.3-q13.43	Loss (2+1)	Shared
	Stem	21q11.2-q22.3	Loss (2+1)	Shared
	1	5p15.33-q35.3	Loss (1+1)	Private
	2	3p26.3-q29	Loss (2+1)	Shared
	3	10p15.3-q26.3	Loss (1+1)	Private
	3	3q11-q25	Loss (2+0)	Private
	4	20p13-q13.33	Loss (2+1)	Private
	5	7p22.3-q36.3	Loss (2+1)	Shared
	6	6p15.5-p15.2	Loss (2+0)	Shared
	7	9p13.3-p13.1	Gain (3+2)	Private
	8	10p15.3-q26.3	Gain (2+2)	Private
	9	9p15.1-p14.1	Loss (2+0)	Private
	10	20p13-q13.33	Loss (1+1)	Private
	11	9q31.1-q34.3	Gain (4+4)	Private
	12	12q13.13-q24.33	Loss (3+1)	Shared
	13	20p12.1-p12.1	Loss (2+1)	Shared
	14	19q13.43-q13.43	Loss (2+0)	Private
	15	20p12.1-p12.1	Loss (1+1)	Private
	16	8p23.2-p23.2	Loss(1+0)	Private
	16	3p26.1-p26.1	Loss (1+1)	Private
	16	11p15.5-p11.12v2	Loss (2+0)	Private
	17	3q13.31-q13.31v1	Loss (1+1)	Private
	18	20p13-q13.33v1	Loss (2+1)	Private
	19	19q13.13-q24.33	Loss (2+1)	Private
	20	18q11.1-q23	Gain (3+2)	Private
	21	3q13.31-q13.31v3	Loss (1+1)	Private
	22	2p23.3-q37.3	Loss (1+1)	Private
	23	22q11.1-q13.33	Loss (2+1)	Private
	24	18p11.32-p11.21	Loss (2+1)	Private
	25	3q13.31-q13.31v2	Loss (1+1)	Private
	26	14q11.2-q32.33	Loss (2+0)	Private
	26	20p13-q13.33v2	Loss (2+1)	Private
	27	18p11.32-q23	Loss (2+1)	Private
	28	3p26.1-p26.1v1	Loss (1+1)	Private
	29	20p13-q13.33v3	Loss (2+1)	Private
	30	11p15.5-p11.12v3	Loss (2+0)	Private
	31	9p24.1-p24.1	Loss (1+0)	Private
	31	9p23-p23	Loss (1+0)	Private
	31	13q11-q34	Loss (2+0)	Private
	31	20p12.1-p12.1v1	Loss (2+1)	Private
	32	3p26.1-p26.1v2	Loss (1+1)	Private
	33	11p15.5-p11.12v1	Loss (2+0)	Private

Supplementary Figure 2. Clonal landscapes of cisplatin treated PDX tumors. a:I. Genetically identical founder populations of tumor cells derived from a patient with *MYCN* amplified neuroblastoma were subcutaneously injected in immunosuppressed mice to form cohorts of untreated tumors and cisplatin-treated tumors. Cisplatin treatment resulted in either stationary or progressive disease, splitting the treated group into two cohorts. A fourth group of tumors were first allowed free growth, followed by incomplete resection and regrowth. PDXs selected for analyses are marked with filled circles. **a:II.** Maximum likelihood phylogeny based on copy number aberration (CNA) profiles and clonal deconvolution. **a:III** Bar charts denote the composition of detected clones based on CNA profiles in biopsies A (left column) and B (right column) from each xenograft. The y axis are clone sizes and asterisks denote clonal sweeps encompassing both A and B samples. Above each bar chart is stated the number of private aberrations with clone sizes within parentheses. Colors denote deconvolved clones as specified in the ideogram to the upper left. **b:I** Tables of copy number aberrations. All aberrant segments were summarized and grouped across clones and subclones and described by corresponding colors and symbols, and further categorized by group of aberrations, location, type and classified as shared or private. A circle or star denote shared or private aberrations, respectively. Greek letters were applied for non-stem aberrations. Location corresponds to the chromosomal start and end point for the aberration (hg 19). The types of aberrations were gain, amp (amplification), loss and Cnni (copy number neutral imbalance). **b:II.** All unique subclones observed were summarized and annotated using the main clonal background and daughter subclone symbols. **b:III.** Detailed annotation of clones observed in each sample. All unique clones were given a sample name and the corresponding subclonal aberrations are written after "-s". **c:I.** Ideogram of clonal evolution. A dashed line denotes a clonal sweep and a solid line was used for the emergence of subclones. Significant aberrations for neuroblastoma were annotated. **c:II.** Subclone maps of PDX tumors based on CNA data. Large grey circles indicate 100% prevalence (clone size), and small circles correspond to a 10% clone size. Clonal sweeps are described by giving the edge of the large circle the same color as the last sweeping aberration. For additional information see **Supplementary Data 2c**. **d:I.** Relationship of clones and presence of key mutations are illustrated as in main **Fig. 3b**, with the additional information of names of branches and subbranches. **d:II**. Subclone maps of PDX tumors based on WES data. Stem mutations are shown as big grey circles in the background of each sample. The color of the border is changed to the color of sweeping clone in samples where a clonal sweep has taken place. The mother, daughter and private mutations are visualized as in (d:I). Each ring corresponds to a clone size of 10%. **d:III.** A phylogenetic tree based on the maximum likelihood method displaying the relationship of between clones. The branches are colored according to the mother and daughter clones (see d:I and d:II). Branches leading to private mutations are marked with a thin black line. **d:IV.** For additional information see **Supplementary Data 2d** and **e**. The sizes of mother clones in each sample are pictured as bars next to each other. Daughter clones are placed as triangles inside the mother clones; they are placed above each other if parallel and placed as a smaller triangle inside the most recent mother clone if they are nested. The number and clone size of private mutations in a specific mother clone is indicated above the column and an asterisk (*) above a column indicates the presence of a clonal sweep. **e:I-II.** The number of genetic changes detected in PDX tumors after cisplatin treatment. Two samples from each tumor were analyzed for CNAs while the two samples were combined prior to WES analyses. Results based on copy number data are shown to the left, and mutation frequencies obtained from WES analyses are summarized to the right. The top plots demonstrate the total number of segmental aberrations (I) or mutations (II), and the lower plots show the total number of private aberrations identified by each technique. Treatment groups are indicated on the x-axis; for details see Methods. Mann-Whitney U-test (two-sided) were performed, and a p-value under 0.05 was considered to

be significant. Black bars denote median values. **e:III**. The number of variants as function of time. The y-axis of the scatter plots denotes the number of CNAs (plots to the left) or SNV/indels (plots to the right), and the time to sampling was assigned to the x-axis. The grey shades show a 95% confidence interval. The linear correlation function and coefficient of determination (R^2) are annotated inside the panels. Source data are provided as a Source Data file. **f:I.** Growth curves of PDX1 tumors progressing under COJEC treatment (COJEC1-4) and free-growth controls (C1-C3). **f:II.** The top plot demonstrates the total number of copy number aberrations, and the lower plot shows the total number of private aberrations identified. Treatment groups are indicated on the x-axis. Black bars denote median values. **f:III-IV.** Phylogenetic ideogram (II) and subclone composition (III) emerging in PDX1 based on CNA profiles. CNA clusters distinguishing different subpopulations of tumor cells (circles) are annotated in the ideogram with details given in **Supplementary Fig. 2g**. From each PDX tumor, one biopsy was analyzed, corresponding to one large circle in (III), in which each small circle corresponds to 10% of tumor cells. **g.** List of CNAs from COJEC1-4 and C1-C3, present in all tumor cells (stem) or distinguishing different subclones in figures f:III-IV.

Suppl Fig 3

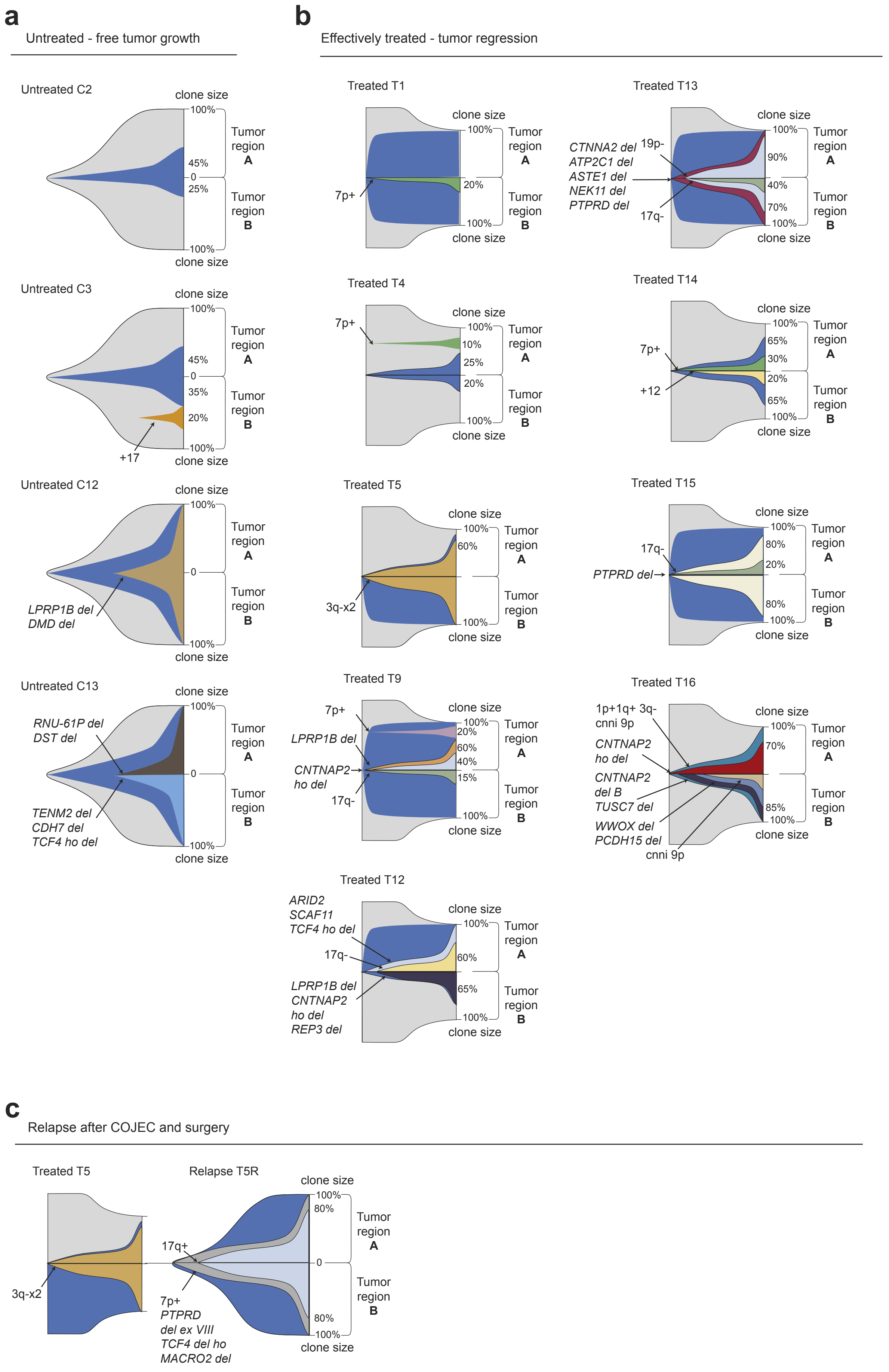
a
Untreated - free tumor growth
Untreated C2
clone size
100%
45%
0
25%
100%
clone size
Tumor region A
Tumor region B
Untreated C3
clone size
100%
45%
0
35%
20%
100%
clone size
+17
Tumor region A
Tumor region B
Untreated C12
clone size
100%
0
100%
clone size
LPRP1B del
DMD del
Tumor region A
Tumor region B
Untreated C13
clone size
100%
0
100%
clone size
RNU-61P del
DST del
TENM2 del
CDH7 del
TCF4 ho del
Tumor region A
Tumor region B
b
Effectively treated - tumor regression
Treated T1
clone size
100%
20%
100%
clone size
7p+
Tumor region A
Tumor region B
Treated T13
clone size
100%
90%
40%
70%
100%
clone size
CTNNA2 del
ATP2C1 del
ASTE1 del
NEK11 del
PTPRD del
19p-
17q-
Tumor region A
Tumor region B
Treated T4
clone size
100%
10%
25%
20%
100%
clone size
7p+
Tumor region A
Tumor region B
Treated T14
clone size
100%
65%
30%
20%
65%
100%
clone size
7p+
+12
Tumor region A
Tumor region B
Treated T5
clone size
100%
60%
100%
clone size
3q-x2
Tumor region A
Tumor region B
Treated T15
clone size
100%
80%
20%
80%
100%
clone size
17q-
PTPRD del
Tumor region A
Tumor region B
Treated T9
clone size
100%
20%
60%
40%
15%
100%
clone size
7p+
LPRP1B del
CNTNAP2 ho del
17q-
Tumor region A
Tumor region B
Treated T16
clone size
100%
70%
85%
100%
clone size
1p+1q+ 3q-
cnni 9p
CNTNAP2 ho del
CNTNAP2 del B
TUSC7 del
WWOX del
PCDH15 del
cnni 9p
Tumor region A
Tumor region B
Treated T12
clone size
100%
60%
65%
100%
clone size
ARID2
SCAF11
TCF4 ho del
17q-
LPRP1B del
CNTNAP2 ho del
REP3 del
Tumor region A
Tumor region B
c
Relapse after COJEC and surgery
Treated T5
3q-x2
Relapse T5R
clone size
100%
80%
0
80%
100%
clone size
17q+
7p+
PTPRD del ex VIII
TCF4 del ho
MACRO2 del
Tumor region A
Tumor region B

Supplementary Figure 3. Clonal landscapes of COJEC-responding PDX tumors. **a.** PDX tumors from mice only injected with saline. Fish plots denote clonal landscapes with annotations as in Fig. 4. Four representative controls are shown here, a fifth in Fig. 4b, and the remaining four in Fig. 3c as they acted as controls also for that experiment. **b.** PDX tumors from mice treated with rapid COJEC (carboplatin, etoposide, vincristine, cisplatin and cyclophosphamide) under the protocol delineated in Fig. 4a. **c.** Clonal landscapes in relapsing treated PDX tumor T5, showing replacement of a clone with 3q deletions (3q-x2) in the primary with a set of clones harboring an additional copy of 17q, deletion of *PTPRD* exon VIII and a second hit in *TCF4*, leading to homozygous deletion in that gene.

Suppl Fig 4

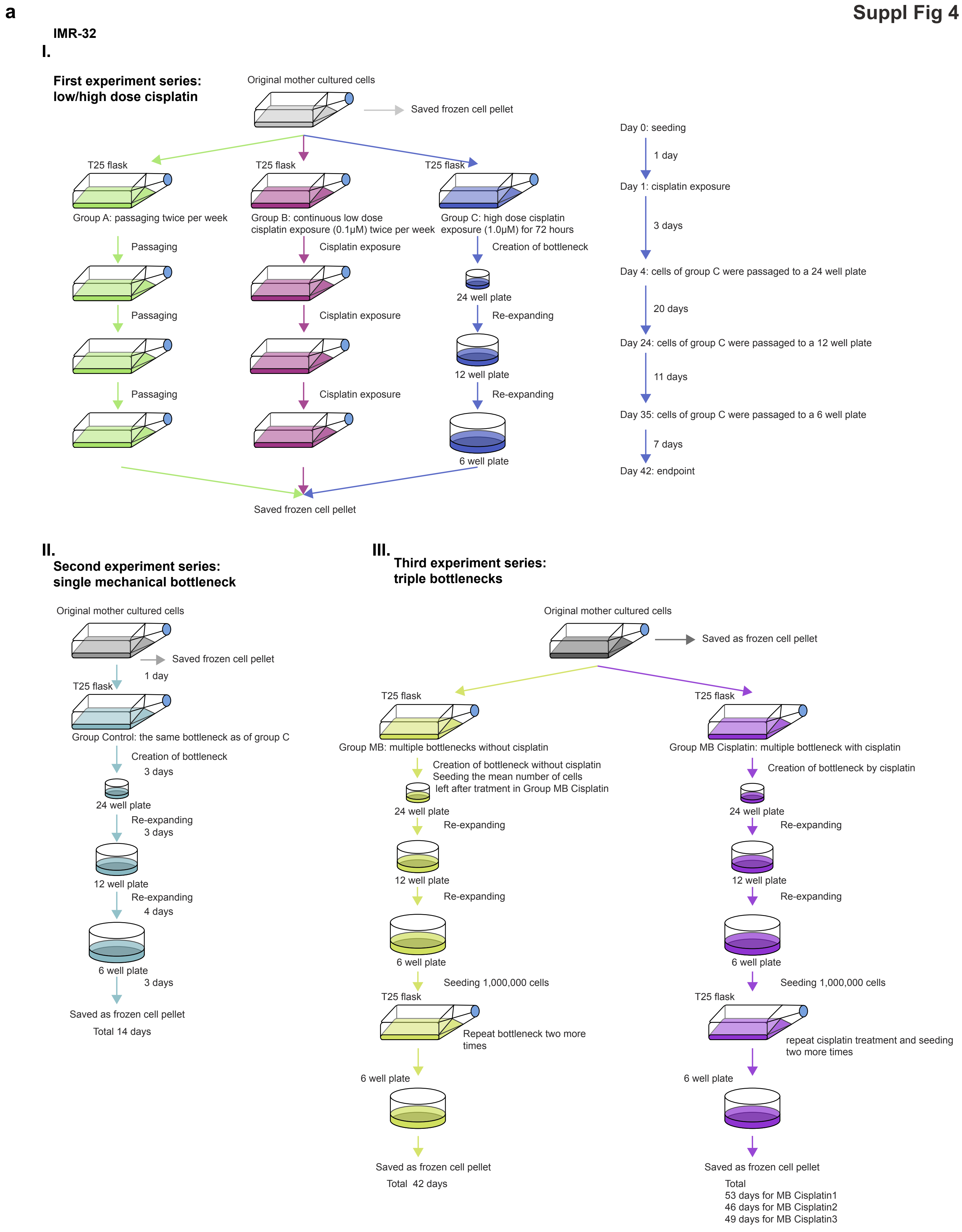
a
IMR-32
I.
First experiment series:
low/high dose cisplatin
Original mother cultured cells
Saved frozen cell pellet
T25 flask
T25 flask
T25 flask
Group A: passaging twice per week
Group B: continuous low dose cisplatin exposure (0.1μM) twice per week
Group C: high dose cisplatin exposure (1.0μM) for 72 hours
Passaging
Cisplatin exposure
Creation of bottleneck
24 well plate
Re-expanding
12 well plate
Re-expanding
6 well plate
Saved frozen cell pellet
Day 0: seeding
1 day
Day 1: cisplatin exposure
3 days
Day 4: cells of group C were passaged to a 24 well plate
20 days
Day 24: cells of group C were passaged to a 12 well plate
11 days
Day 35: cells of group C were passaged to a 6 well plate
7 days
Day 42: endpoint
II.
Second experiment series:
single mechanical bottleneck
Original mother cultured cells
Saved frozen cell pellet
1 day
T25 flask
Group Control: the same bottleneck as of group C
Creation of bottleneck
3 days
24 well plate
Re-expanding
3 days
12 well plate
Re-expanding
4 days
6 well plate
3 days
Saved as frozen cell pellet
Total 14 days
III.
Third experiment series:
triple bottlenecks
Original mother cultured cells
Saved as frozen cell pellet
T25 flask
T25 flask
Group MB: multiple bottlenecks without cisplatin
Group MB Cisplatin: multiple bottleneck with cisplatin
Creation of bottleneck without cisplatin
Seeding the mean number of cells
left after tratment in Group MB Cisplatin
Creation of bottleneck by cisplatin
24 well plate
Re-expanding
12 well plate
Re-expanding
6 well plate
Seeding 1,000,000 cells
T25 flask
Repeat bottleneck two more times
repeat cisplatin treatment and seeding two more times
6 well plate
Saved as frozen cell pellet
Total 42 days
Total
53 days for MB Cisplatin1
46 days for MB Cisplatin2
49 days for MB Cisplatin3

b Key to subclones - First experiment series

I.

Color	Group	Location	Type	Shared/Private
	STEM	chr1:1-24180606	Loss	Shared
	STEM	chr1:43647149-51000919	Loss	Shared
	STEM	chr1:51007213-249224684	Gain	Shared
	STEM	chr2:14738098-16125757	Amp	Shared
	STEM	chr2:29692304-29924102	Amp	Shared
	STEM	chr2:53398553-53601790	Amp	Shared
	STEM	chr2:66719502-67709245	Amp	Shared
	STEM	chr2:69135189-69402138	Amp	Shared
	STEM	chr5:153564069-154139413	Loss	Shared
	STEM	chr7:147377107-148161812	Loss	Shared
	STEM	chr10:33960055-34362459	Gain	Shared
	STEM	chr15:42888126-102429112	Gain	Shared
	STEM	chr16:69799164-90155062	Loss	Shared
	STEM	chr17:40418866-81041938	Gain	Shared
	α1	chr1:24447115-43642216	Cnni	Shared
	α1	chr12:1-133778166	Cnni	Shared
	α2	chr6:148313994-170919482	Gain	Shared
	α3	chr6:1-148259788	Gain	Shared
	α4	chr3:125642302-197851986	Gain	Shared
	α5	chr7:1-19390487	Gain	Shared
	β1	chr1:24447115-43642216	Loss	Shared
	β1	chr11:64859401-89611346	Gain	Shared
	β1	chr11:89904869-134938470	Loss	Shared
	γ1	chr6:1-170919482	Cnni	Private
	γ2	chr6:1-63867780	Cnni	Private
	γ2	chr6:64121071-170919482	Cnni	Private
	γ3	chr6:1-148259788	Cnni	Private
	γ3	chr6:148313994-170919482	Cnni	Private

II.

Symbol	Main clone	Subclone	Symbol	Main clone	Subclone
	STEM	α1		STEM, α1	α2, α3
	STEM	α1, α2		STEM, α1	α2, α3, α4, α5
	STEM	α1, α2, α3		STEM, α1	γ1
	STEM	α1, α2, α3 α4		STEM, α1	γ2
	STEM	α1, α2, α3 α4, α5		STEM, α1, α2	
	STEM	β1		STEM, α1, α2	α3
	STEM	α1, γ3		STEM, α1, α2	α3, α4
	STEM, α1			STEM, α1, α2, α3	
	STEM, α1	α2		STEM, α1, α2, α3	α4

III.

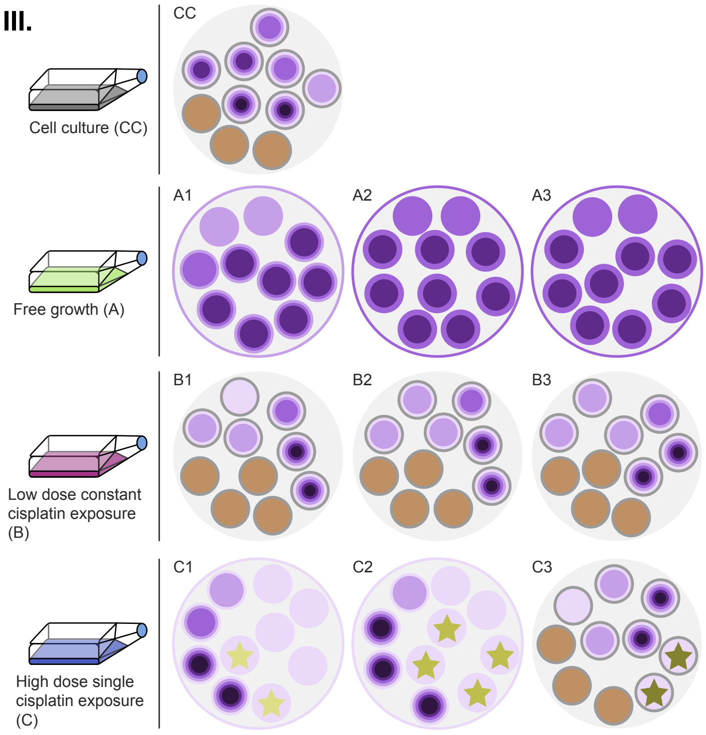

IV.

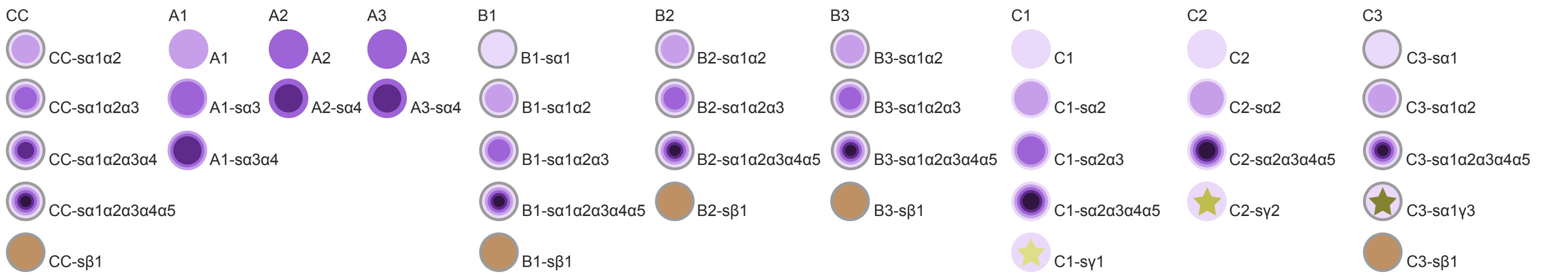

c

Key to subclones - Second experiment series

I.

Color	Group	Location	Type	Shared/Private
	STEM	chr1:1-24180606	Loss	Shared
	STEM	chr1:24447115-43642216	Cnni	Shared
	STEM	chr1:43647149-51000919	Loss	Shared
	STEM	chr1:51007213-249224684	Gain	Shared
	STEM	chr2:14738098-16125757	Amp	Shared
	STEM	chr2:29692304-29924102	Amp	Shared
	STEM	chr2:53398553-53601790	Amp	Shared
	STEM	chr2:66719502-67709245	Amp	Shared
	STEM	chr2:69135189-69402138	Amp	Shared
	STEM	chr5:153564069-154139413	Loss	Shared
	STEM	chr7:147377107-148161812	Loss	Shared
	STEM	chr10:33960055-34362459	Gain	Shared
	STEM	chr12:1-133778166	Cnni	Shared
	STEM	chr15:42888126-102429112	Gain	Shared
	STEM	chr16:69799164-90155062	Loss	Shared
	STEM	chr17:40418866-81041938	Gain	Shared
	α1	chr6:1-148259788	Gain	Shared
	α1	chr6:148313994-170919482	Gain	Shared
	α2	chr3:125642302-197851986	Gain	Shared
	α3	chr7:1-19390487	Gain	Shared
	α4	chr13:90191061-115107733	Gain	Shared
	β1	chr11:64859401-89611346	Gain	Shared
	β1	chr11:89904869-134938470	Loss	Shared

II.

Symbol	Main clone	Subclone
	STEM	
	STEM	α1
	STEM	α1, α2
	STEM	α1, α2, α3
	STEM	α1, α2, α3, α4
	STEM	β1

III.

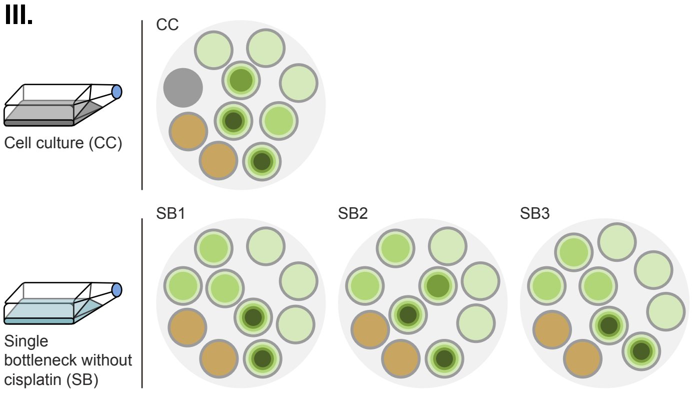

IV.

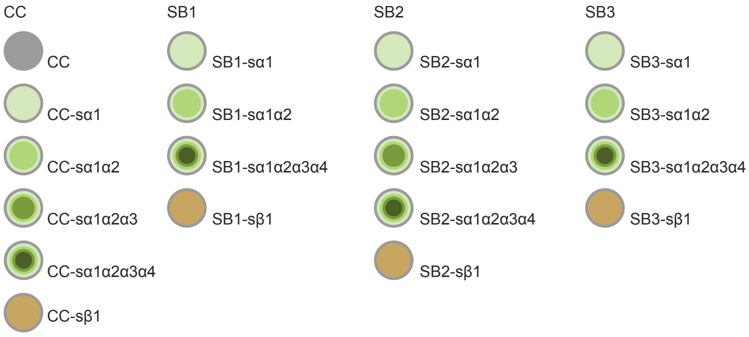

d

Key to subclones - Third experiment series

I.

Color	Group	Location	Type	Shared/Private
	STEM	chr1:1-24180606	Loss	Shared
	STEM	chr1:43647149-51000919	Loss	Shared
	STEM	chr1:51007213-249224684	Gain	Shared
	STEM	chr2:14738098-16125757	Amp	Shared
	STEM	chr2:29692304-29924102	Amp	Shared
	STEM	chr2:53398553-53601790	Amp	Shared
	STEM	chr2:66719502-67709245	Amp	Shared
	STEM	chr2:69135189-69402138	Amp	Shared
	STEM	chr5:153564069-154139413	Loss	Shared
	STEM	chr7:147377107-148161812	Loss	Shared
	STEM	chr10:33960055-34362459	Gain	Shared
	STEM	chr15:42888126-102429112	Gain	Shared
	STEM	chr16:69799164-90155062	Loss	Shared
	STEM	chr17:40418866-81041938	Gain	Shared
	α1	chr1:24447115-43642216	Cnni	Shared
	α1	chr12:1-133778166	Cnni	Shared
	α2	chr6:148313994-170919482	Gain	Shared
	α3	chr6:1-148259788	Gain	Shared
	α4	chr3:125642302-197851986	Gain	Shared
	α5	chr7:1-19390487	Gain	Shared
	β1	chr1:24447115-43642216	Loss	Shared
	β1	chr11:64859401-89611346	Gain	Shared
	β1	chr11:89904869-134938470	Loss	Shared
	γ1	chr6:1-148259788	Cnni	Shared
	γ1	chr6:148313994-170919482	Cnni	Shared
	γ2	chr13:19436286-81800341	Loss	Shared
	δ1	chr13:26924206-98273079	Loss	Private
	δ2	chr13:81836702-105749055	Gain	Private
	δ3	chr13:81836702-105749055	Cnni	Private

II.

Symbol	Main clone	Subclone	Symbol	Main clone	Subclone
	STEM	α1		STEM	α1, α2, α3 α4, δ1
	STEM	α1, α2		STEM	α1, α2, α3 α4, γ2, δ2
	STEM	α1, α2, α3		STEM	α1, α2, α3 α4, γ2, δ3
	STEM	α1, α2, α3 α4		STEM, α1	
	STEM	α1, α2, α3 α4, α5		STEM, α1	α2
	STEM	β1		STEM, α1	α2, α3
	STEM	α1, γ1		STEM, α1	α2, α3, α4

III.

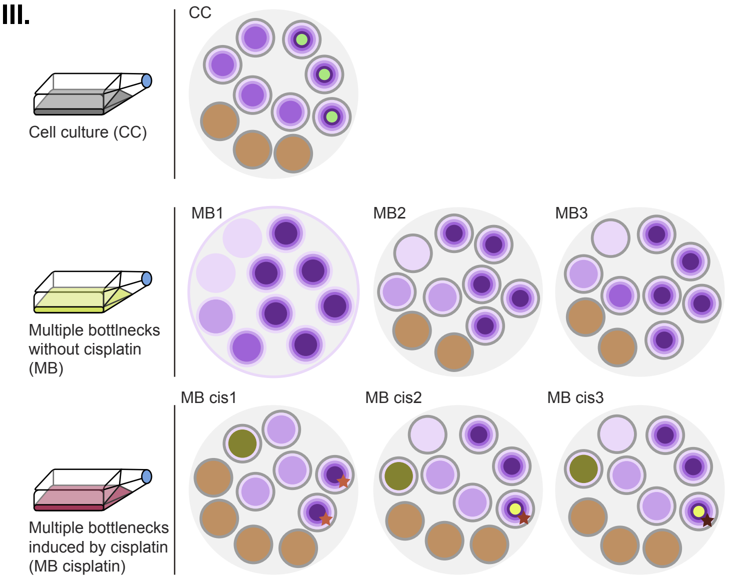

IV.

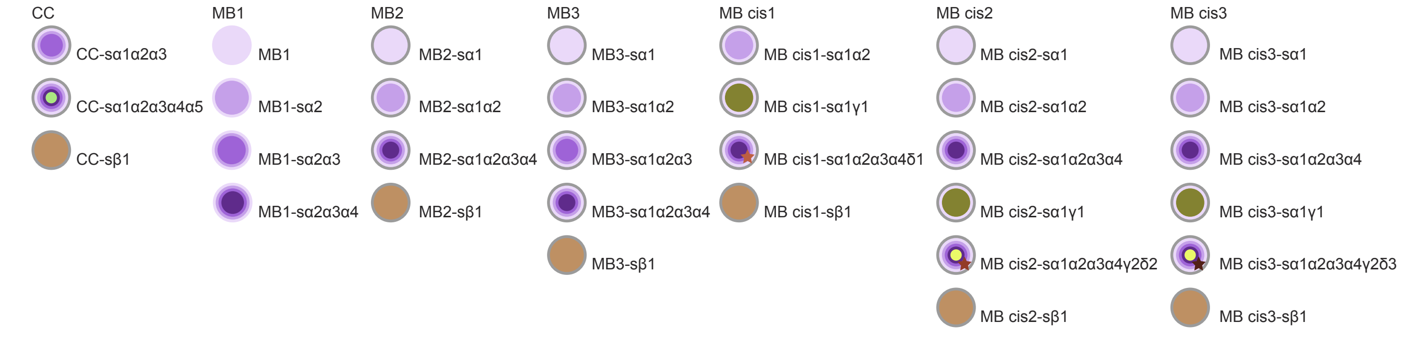

Suppl Fig 4

e

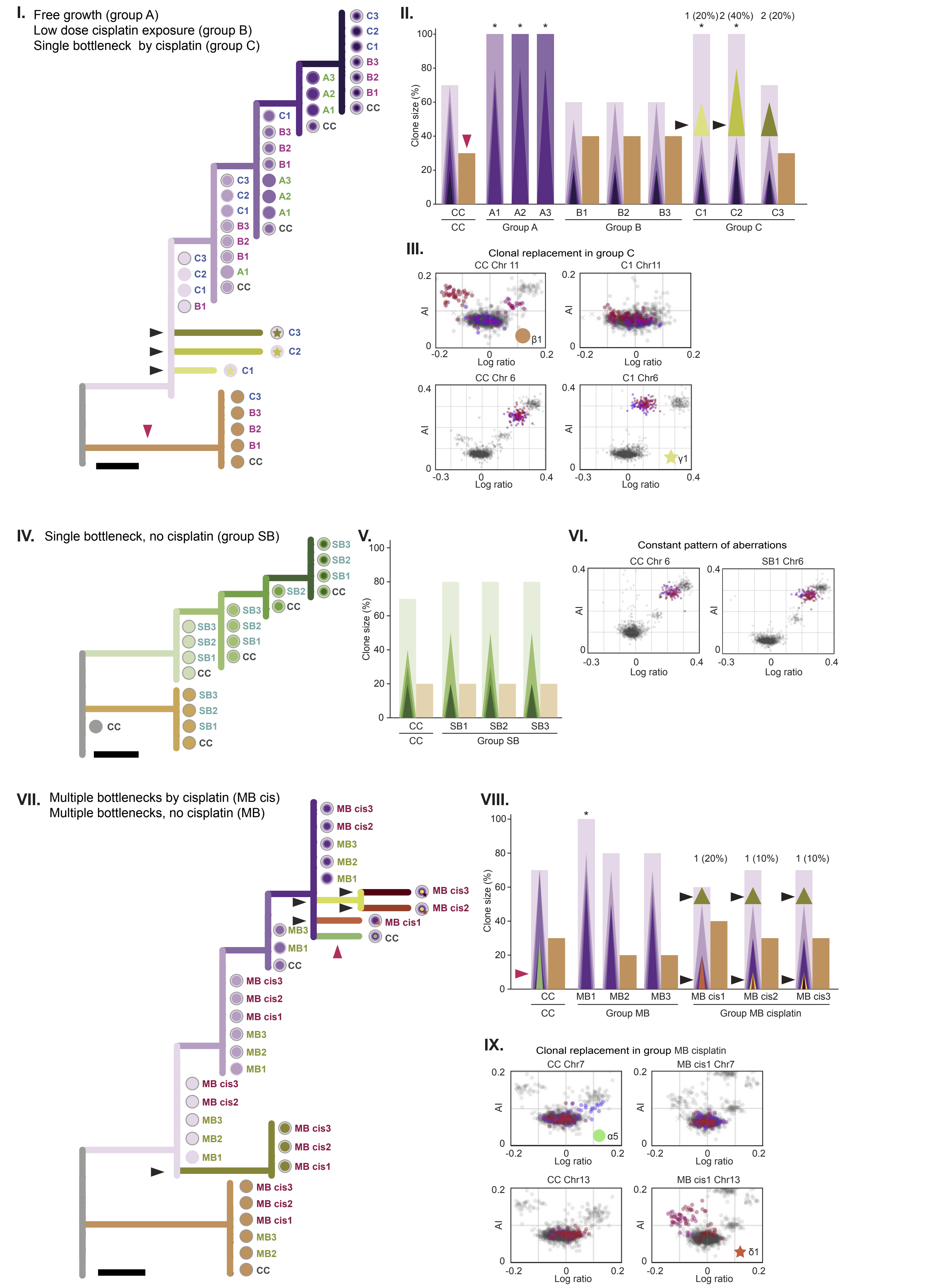
I. Free growth (group A)
Low dose cisplatin exposure (group B)
Single bottleneck by cisplatin (group C)
II.
Clone size (%)
1 (20%)
2 (40%)
2 (20%)
CC
A1
A2
A3
B1
B2
B3
C1
C2
C3
Group A
Group B
Group C
III.
Clonal replacement in group C
CC Chr 11
C1 Chr11
CC Chr 6
C1 Chr6
AI
Log ratio
β1
γ1
IV. Single bottleneck, no cisplatin (group SB)
SB1
SB2
SB3
V.
Group SB
VI.
Constant pattern of aberrations
SB1 Chr6
VII. Multiple bottlenecks by cisplatin (MB cis)
Multiple bottlenecks, no cisplatin (MB)
MB cis1
MB cis2
MB cis3
MB1
MB2
MB3
VIII.
1 (10%)
Group MB
Group MB cisplatin
IX.
Clonal replacement in group MB cisplatin
CC Chr7
MB cis1 Chr7
CC Chr13
MB cis1 Chr13
α5
δ1

f

I.

SK-N-SH
cisplatin-induced single and double bottleneck

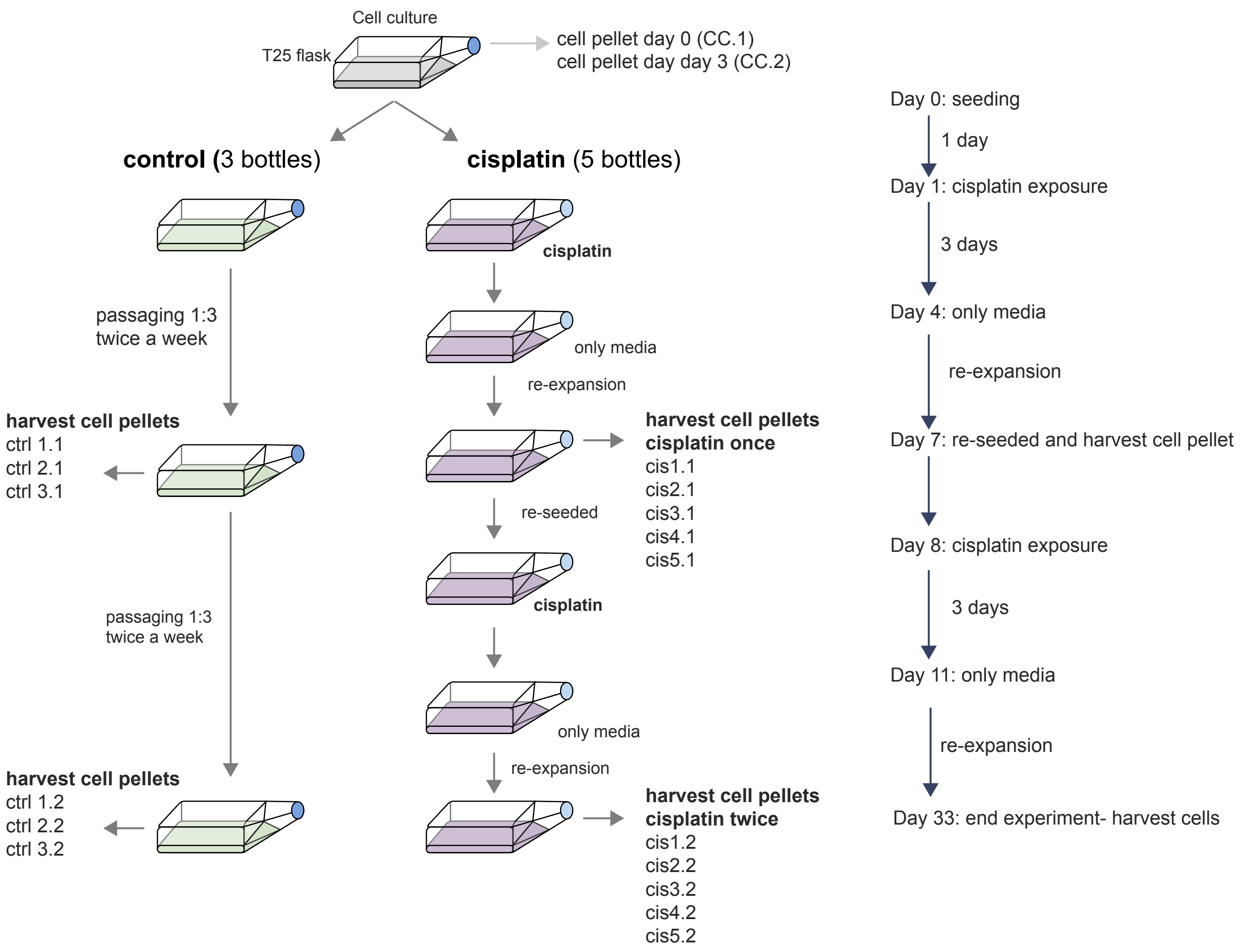

II.

Color	Group	Location	Type
	STEM	22q13q13	loss_1+0
	STEM	22q13q13	cnni_2+0
	STEM	14q13q21	loss_1+0
	STEM	14q21q32	cnni_2+0
	STEM	17q21q25	gain_1+2
	STEM	7 whole	gain_1+2
	1	1q21q44	gain_1+2
	2	2p25p16	gain_1+3
	3	9q33q34	cnni_2+0
	3	11p15p15	gain_1+2
	4	1p36p36	loss_1+0
	4	18p11p11	gain_1+2

g **SK-N-SH**
cisplatin-induced single and double bottleneck

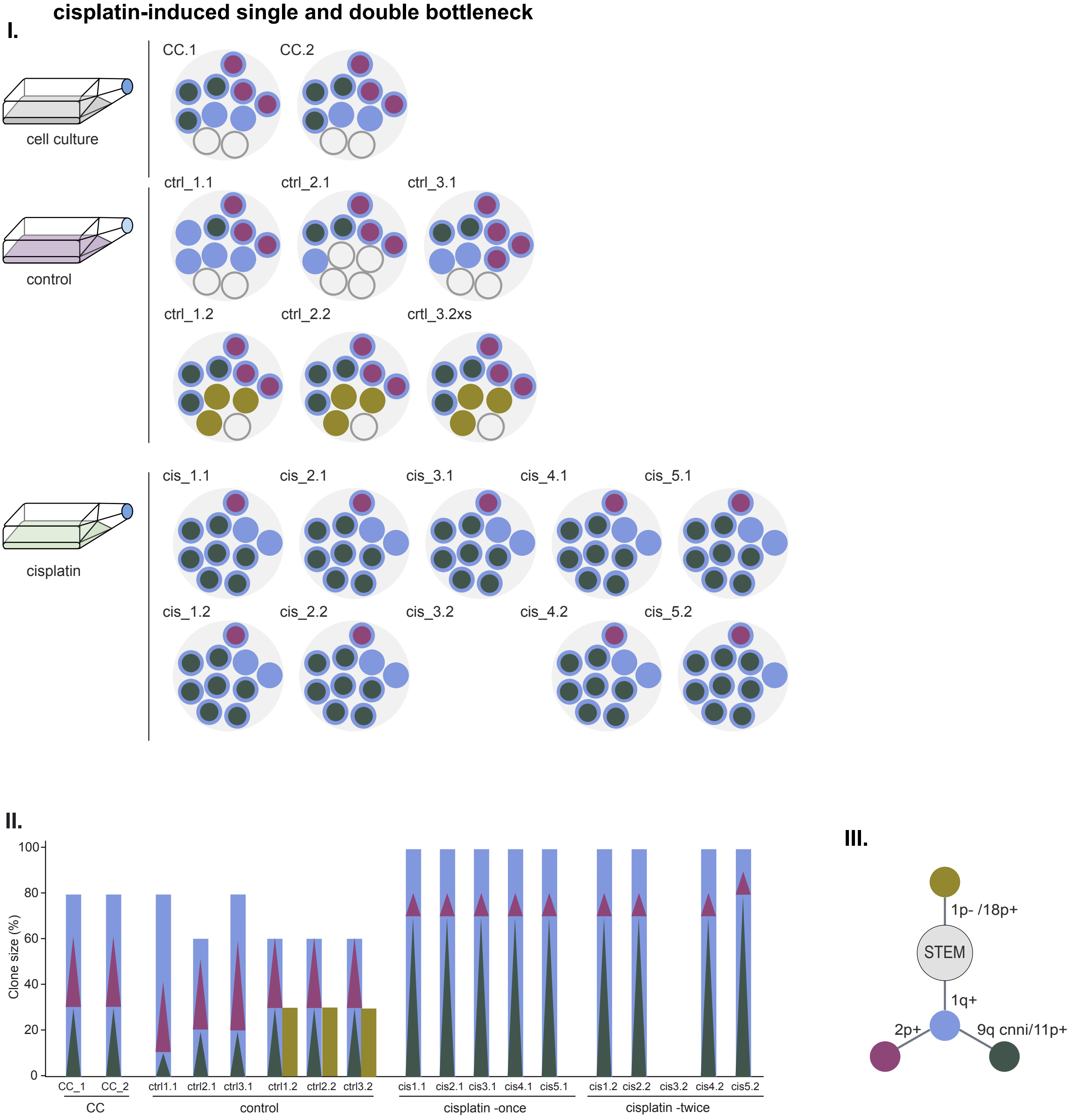

Supplementary Figure 4. Clonal evolution under cisplatin treatment in vitro. The neuroblastoma cell lines IMR-32 and SK-N-SH were used to analyze clonal evolution after chemotherapy treatment *in vitro*. Three experimental setups were performed with the IMR-32 cells, under six different conditions, see Methods for further details regarding the experiments and **Supplementary Data 2h** for raw data. **a:I.** The first setup includes continuous growth without bottleneck (Group A, samples A1-A3); long-term low-dose cisplatin exposure (Group B, samples B1-B3); and a single bottleneck induced by high-dose cisplatin (Group C, samples C1-C3): **a:II.** A single bottleneck without cisplatin (Group Control, samples SB1-SB3): The same number of cells as those surviving 72 h high-dose cisplatin in experiments C1-C3 was collected, re-seeded and allowed to grow. **a:III.** Multiple bottlenecks induced by cisplatin (Group MB Cisplatin, samples MB cis1-MB cis3). Bottlenecks were created by treating the cells with 0.75 μM cisplatin followed by re-growth. This procedure was then repeated two times. This was compared in parallel to multiple bottlenecks without cisplatin (Group MB, samples MB1-MB3). Specifically, three bottlenecks were created by re-seeding the same number of cells as those surviving cisplatin in MB1-MB3 in medium only. Cells from the end point of each experiment and cells from the "mother" culture that were used to start each experiment were collected and frozen. **b:I.** Clonal deconvolution of IMR-32 copy number profiles under varied cisplatin treatment regimens, compared to bottlenecks without cisplatin (restricted passaging), and free growth. All identified aberrant segments were summarized and described by color, group, location, type and classified as shared or private. A circle or star denote shared or private aberration, respectively. Greek letters were applied for non-stem aberrations. Location corresponds to chromosome with a start and end point for each CNA. **b:II.** All subclones observed in each data set were summarized and annotated using the main clonal background and daughter subclone symbols. **b:III.** Maps of subclone content where big grey circles indicate clonal background (>90% of cells in a sample), and small circles correspond to a 10% clone size. When a clonal sweep was confirmed, the edge of the big circle was colored by the corresponding color of the last sweeping aberration. **b:IV.** Detailed annotation of clones observed in each sample. All unique clones were given a name. The names of the corresponding subclonal aberrations are written after "-s". **c:I-IV.** Clonal deconvolution of IMR-32 copy number profiles under a single mechanical bottleneck. **d:I-IV.** Clonal deconvolution of IMR-32 copy number profiles under multiple bottlenecks with or without cisplatin. **e:I-VIII.** Detailed summary; I-III show free growth, low dose cisplatin, and single bottleneck induced by cisplatin; IV-VI show single bottleneck in the absence of cisplatin; VII-IX show multiple bottlenecks in the presence of cisplatin. The results generated from each experiment are described by a phylogenetic tree, a bar chart and a representative log2/ allelic imbalance plot. In the trees (**e:I, IV, VII**), black arrowheads indicate subclones that emerged and red arrows subclones that disappeared under each experiment. Letters at the end of the branches denote different clones/subclones, and clones/subclones resulting from the same experimental set up have a specific color. Scale bars (black lines) correspond to one aberration. In the bar charts (e:II, V, VIII), the y-axes correspond to clone size. Rectangles denote mother clones and triangles daughter clones. The numbers above each bar indicate the total number of private aberrations followed by a corresponding clone size in brackets. An asterisk (*) indicates the presence of a clonal sweep. CCR is indicated by arrowheads. Representative log2/allelic imbalance plots (e:III, VI, IX) demonstrating features of CCR. The x-axes denote the base 2 logarithm value (Log ratio) for copy number content and y-axis the allelic imbalance (AI). Lost aberrations under cisplatin treatment are shown in the top rows and gained aberrations in the bottom rows. In e:VI, a randomly chosen chromosome is shown to demonstrate the absence of replacement. Symbols in the corner of some of the plots correspond to detailed information about each aberration that can be found in b-d. **f-g** Experimental setup and identified clonal landscape of the SK-N-SH mother culture (CC), control cells (ctrl) and in cisplatin treated

cultures (cis). For raw data and additional information, see **Supplementary Data 2i**. **f:I** The cells were treated with cisplatin at a final concentration of 4 μM for three days, allowed to re-expand, treated again at the same condition and re-expanded to semi-confluency one more time. Samples were taken from the cultures after each re-expansion, and in parallel from untreated cells. **f:II** Identified subclones were annotated with color and group and aberrations in the subclones were annotated with location (cytoband) and type. **g:I** Subclone composition in the SK-N-SH mother culture (CC), control cells (ctrl) and cisplatin treated cells (cis). See b:III for explanation to the illustration. **g:II** Barchart demonstrating clone sizes and clonal relationships. Rectangles illustrate mother clones and triangles indicate parallel daughter clones nested in the mother clone. **g:III**. Phylogenetic ideogram showing the relationship between subclones and the identified aberrations.

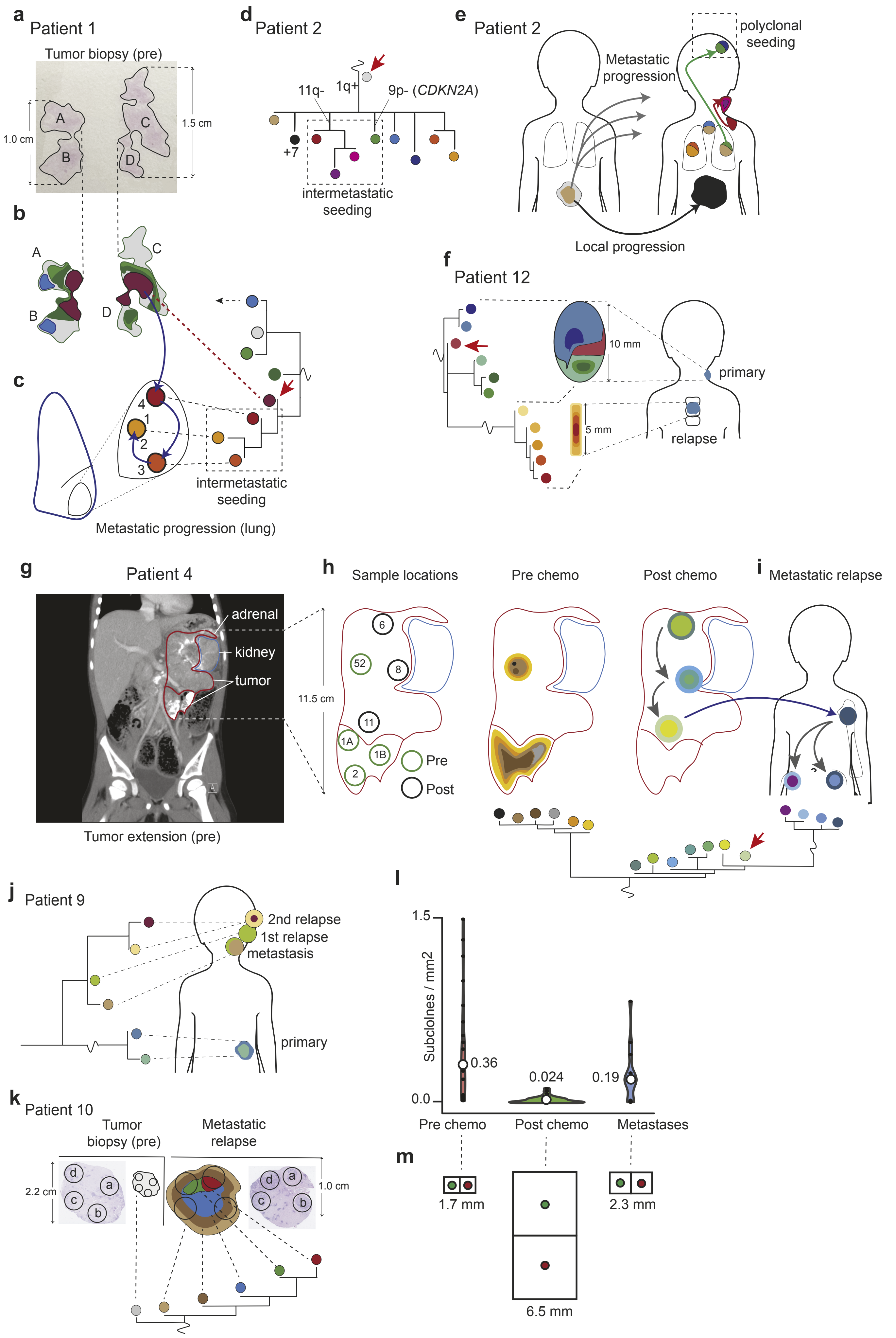
a
Patient 1
Tumor biopsy (pre)
A
B
C
D
1.0 cm
1.5 cm
b
A
B
C
D
c
4
1
2
3
intermetastatic
seeding
Metastatic progression (lung)
d
Patient 2
11q-
1q+
9p- (CDKN2A)
+7
intermetastatic
seeding
e
Patient 2
Metastatic
progression
polyclonal
seeding
Local progression
f
Patient 12
10 mm
primary
5 mm
relapse
g
Patient 4
adrenal
kidney
tumor
Tumor extension (pre)
h
Sample locations
Pre chemo
Post chemo
6
52
8
11
1A
1B
2
11.5 cm
Pre
Post
i
Metastatic relapse
j
Patient 9
2nd relapse
1st relapse
metastasis
primary
k
Patient 10
Tumor
biopsy (pre)
Metastatic
relapse
d
a
c
b
2.2 cm
1.0 cm
l
1.5
0.0
Subclolnes / mm2
0.36
0.024
0.19
Pre chemo
Post chemo
Metastases
m
1.7 mm
6.5 mm
2.3 mm

Supplementary Figure 5. Subclone territories in patients with metastatic disease. a-k. Phylogenies are combined with anatomic charts and histopathological images to map subclone territories before (pre) and after (post) treatment. Patients 1 (a-c) and 2 (d-e) had disease progressing under therapy, while patients 12 (f), 4 (g-i), 9 (j) and 10 (k) relapsed after a clinically disease-free period, with Patient 9 having two relapses at the site (mandible) of a metastasis detected at presentation. For cases with multiple metastases, metastatic routes inferred by the phylogenies are drawn (b-c, e) implying both polyclonal seeding from the primary tumor and intermetastatic seeding. A sufficient number of samples to draw detailed maps of clonal territories in the primary tumor site were present in Patients 1 (b), 12 (f) and 4 (h; MRI scan), and of a metastasis in Patient 10 (k). In the three patients where treatment response was absent (Patients 1 and 2) or minimal (Patient 4; 20% necrosis) or where chemotherapy was not given (Patient 12), a subclone ancestral to all metastatic sites were identified in the primary tumor (b-c, f, h-I, red arrows), while cases where only 0-3% viable tumor cells were detected after chemotherapy (Patients 9 and 10) did not reveal an ancestral subclone in the primary tumor (j, k). In patients with multiple relapse sites, phylogenetic relationships inferred seeding from a single ancestral metastatic site; in the humerus for Patient 4 (i) and in the mandible in Patient 9 (j). **l.** Comparing cross-sectional areas of samples with the number of subclonal populations found in each sample, allowed the calculation of subclone density. In the violin plots, each biopsy is denoted by black points and medians by white circles. Using median values of samples subdivided according to Pre/Post/Relapse, samples obtained after chemotherapy had lower densities of subclones than pre-chemotherapy (p=0.0087; Mann-Whitney U test, two-tailed) and metastases (p=0.048). Median values are denoted next to each violin plot. Median distance required to move across tissue space from one clone to the other can be extrapolated from clone density (**m**) using a grid model with standard 1 mm section tissue section thickness. Source data are provided as a Source Data file.

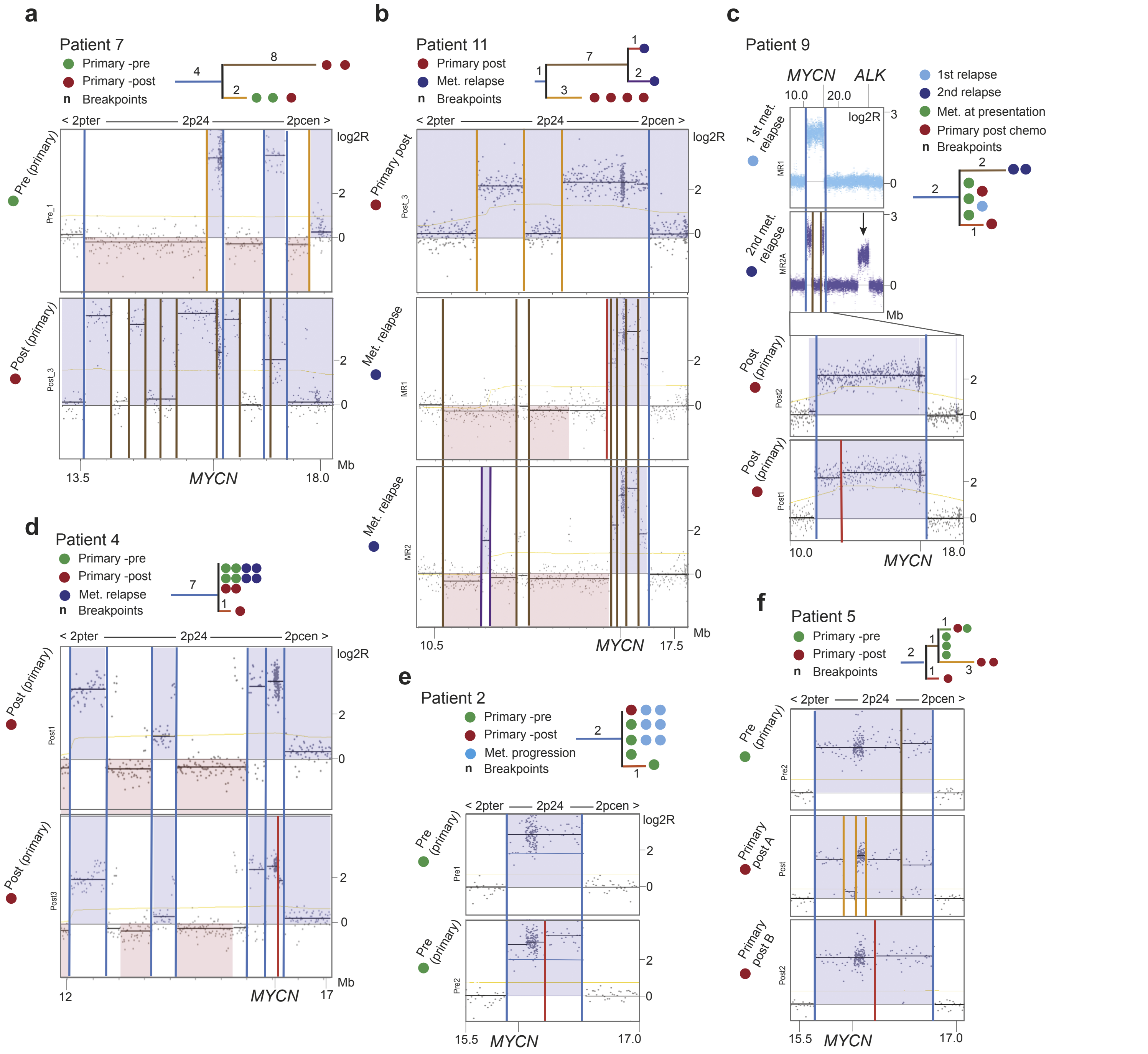
a
Patient 7
Primary -pre
Primary -post
n Breakpoints
< 2pter 2p24 2pcen >
log2R
Pre (primary)
Post (primary)
13.5
MYCN
18.0
Mb
b
Patient 11
Primary post
Met. relapse
n Breakpoints
Met. relapse
10.5
17.5
c
Patient 9
MYCN ALK
10.0 20.0
1st relapse
2nd relapse
Met. at presentation
Primary post chemo
1 st met. relapse
2nd met. relapse
Post (primary)
18.0
d
Patient 4
Met. relapse
12
17
e
Patient 2
Met. progression
15.5
17.0
f
Patient 5
Primary post A
Primary post B

Supplementary Figure 6. Intratumor diversity of *MYCN* amplicons in clinical samples. Intra- and intertumoral diversity of *MYCN* amplicon cassettes. Breakpoints are inferred by shifts in copy number (log2R) in the 2p24 region. Vertical lines orthogonal to genomic maps correspond to breakpoints shared across all samples (blue), common to two or more samples (brown, yellow, green) or unique to a sample (orange). Along each breakpoint map is a maximum parsimony tree based on *MYCN* amplicons only and encompassing all clones detected across all sample times and locations in each patient. Numbers in trees correspond to the number of breakpoints. Pre and post denote diagnostic sampling from untreated tumor and sampling of resection/autopsy specimen after treatment, respectively. **a.** Collateral branching in Patient 7 across samples taken before and after rapid COJEC treatment, where clones from both branches were detected after treatment but only from one branch before treatment. **b.** Collateral branching in Patient 11 with one breakpoint common to all samples and one uniform amplicon structure in the primary tumor replaced in a metastatic relapse by two sets of amplicon cassettes with seven shared breakpoints. **c.** Branching both within the primary tumor and between primary tumor and metastasis in Patient 9, consisting of one breakpoint in a subcompartment of the primary tumor and a deletion in the amplicon cassette (two breakpoints) emerging in a second relapse at a site where metastasis was present already at presentation. In addition to the intra-amplicon deletion, the second relapse also showed de novo amplification of the *ALK* oncogene (arrow). **d.** In Patient 4, variation in amplicon structure is limited to a single region of the primary tumor after chemotherapy. **e.** In Patient 2, there is minimal, linear evolution with one additional breakpoint detected in the primary tumor at sampling prior to chemotherapy. **f.** Patient 5 exhibits extensive variability in breakpoints surrounding *MYCN* in the primary tumor.

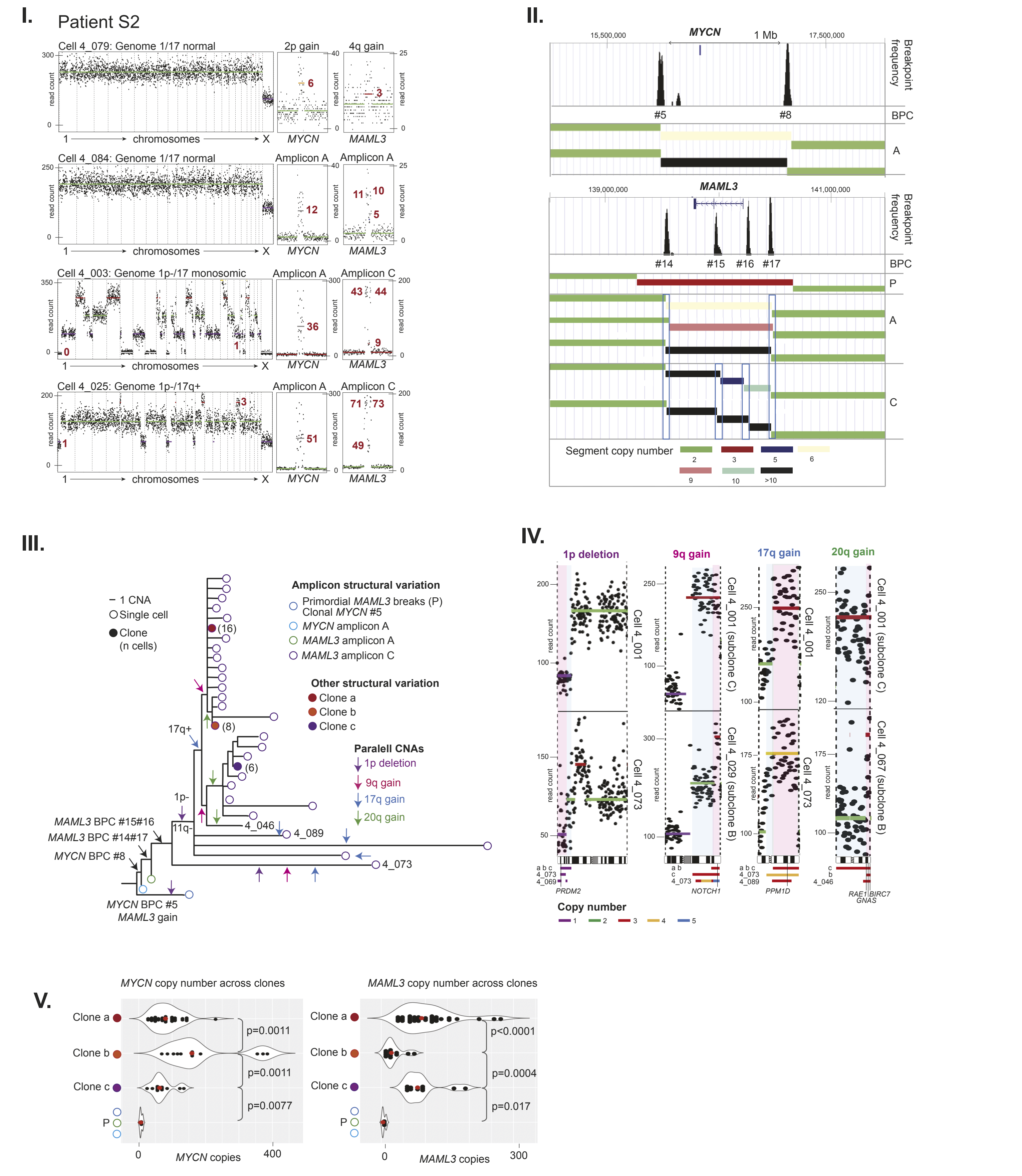
a
I.
Patient S2
Cell 4_079: Genome 1/17 normal
2p gain
4q gain
MYCN
MAML3
chromosomes
Cell 4_084: Genome 1/17 normal
Amplicon A
Amplicon A
Cell 4_003: Genome 1p-/17 monosomic
Amplicon A
Amplicon C
Cell 4_025: Genome 1p-/17q+
Amplicon A
Amplicon C
read count
II.
MYCN
1 Mb
Breakpoint frequency
BPC
MAML3
Segment copy number
III.
1 CNA
Single cell
Clone (n cells)
Amplicon structural variation
Primordial MAML3 breaks (P) Clonal MYCN #5
MYCN amplicon A
MAML3 amplicon A
MAML3 amplicon C
Other structural variation
Clone a
Clone b
Clone c
Paralell CNAs
1p deletion
9q gain
17q gain
20q gain
MAML3 BPC #15#16
MAML3 BPC #14#17
MYCN BPC #8
MYCN BPC #5
MAML3 gain
IV.
1p deletion
9q gain
17q gain
20q gain
Cell 4_001
Cell 4_073
Cell 4_001 (subclone C)
Cell 4_029 (subclone B)
Cell 4_001 (subclone C)
Cell 4_067 (subclone B)
PRDM2
NOTCH1
PPM1D
RAE1 BIRC7
GNAS
Copy number
V.
MYCN copy number across clones
MAML3 copy number across clones
Clone a
Clone b
Clone c
p=0.0011
p=0.0011
p=0.0077
p<0.0001
p=0.0004
p=0.017
MYCN copies
MAML3 copies

Suppl Fig 7

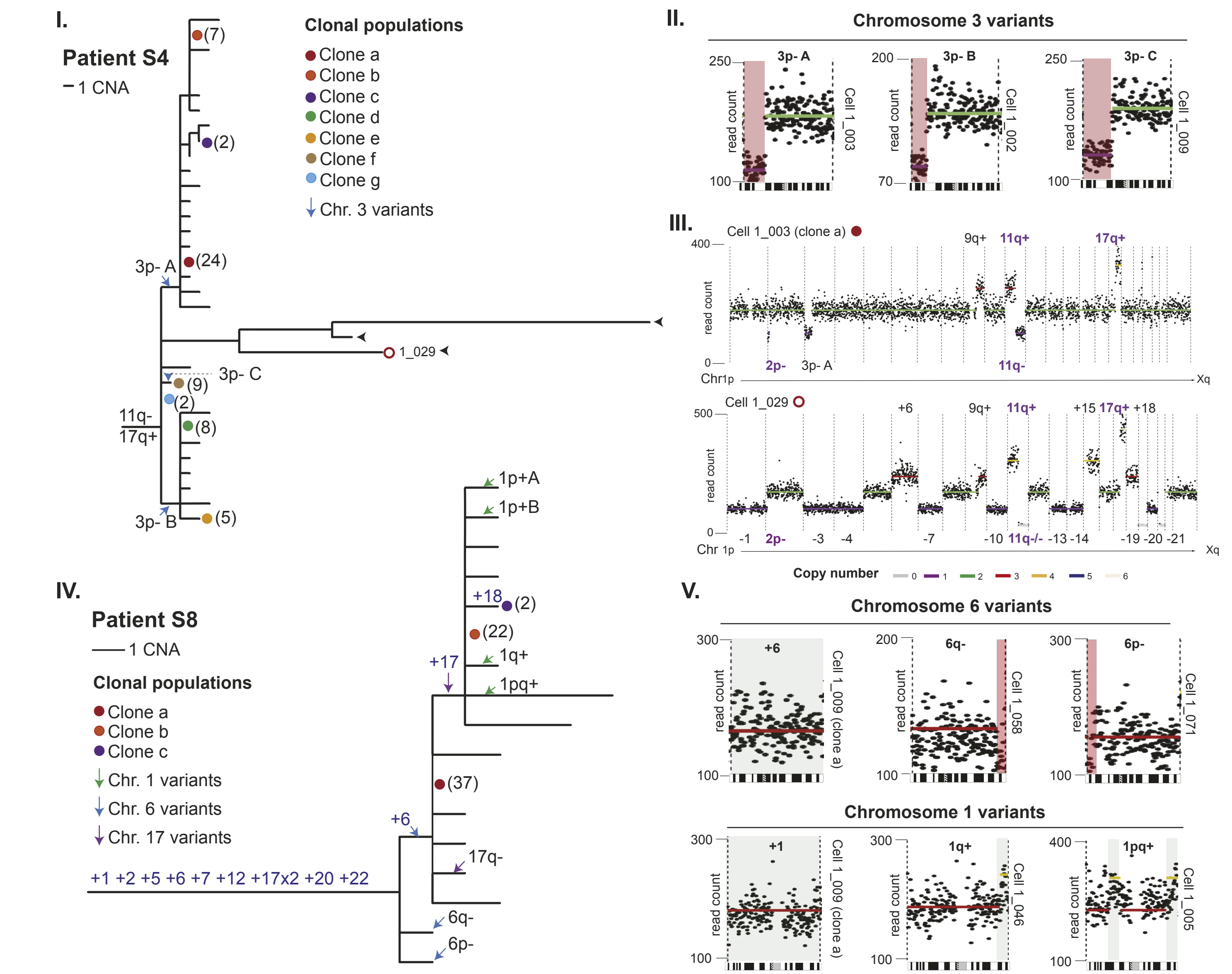
b
I.
Patient S4
1 CNA
Clonal populations
Clone a
Clone b
Clone c
Clone d
Clone e
Clone f
Clone g
Chr. 3 variants
(7)
(2)
(24)
3p- A
1_029
3p- C
(9)
(2)
11q-
17q+
(8)
3p- B
(5)
II.
Chromosome 3 variants
3p- A
3p- B
3p- C
read count
Cell 1_003
Cell 1_002
Cell 1_009
III.
Cell 1_003 (clone a)
9q+
11q+
17q+
2p-
3p- A
11q-
Chr1p
Xq
Cell 1_029
+6
+15
+18
-1
-3
-4
-7
-10
11q-/-
-13
-14
-19
-20
-21
Chr 1p
Copy number
IV.
Patient S8
1 CNA
Clonal populations
Clone a
Clone b
Clone c
Chr. 1 variants
Chr. 6 variants
Chr. 17 variants
+1 +2 +5 +6 +7 +12 +17x2 +20 +22
1p+A
1p+B
+18
(2)
(22)
1q+
+17
1pq+
(37)
17q-
6q-
6p-
V.
Chromosome 6 variants
Cell 1_009 (clone a)
Cell 1_058
Cell 1_071
Chromosome 1 variants
+1
Cell 1_046
Cell 1_005

c

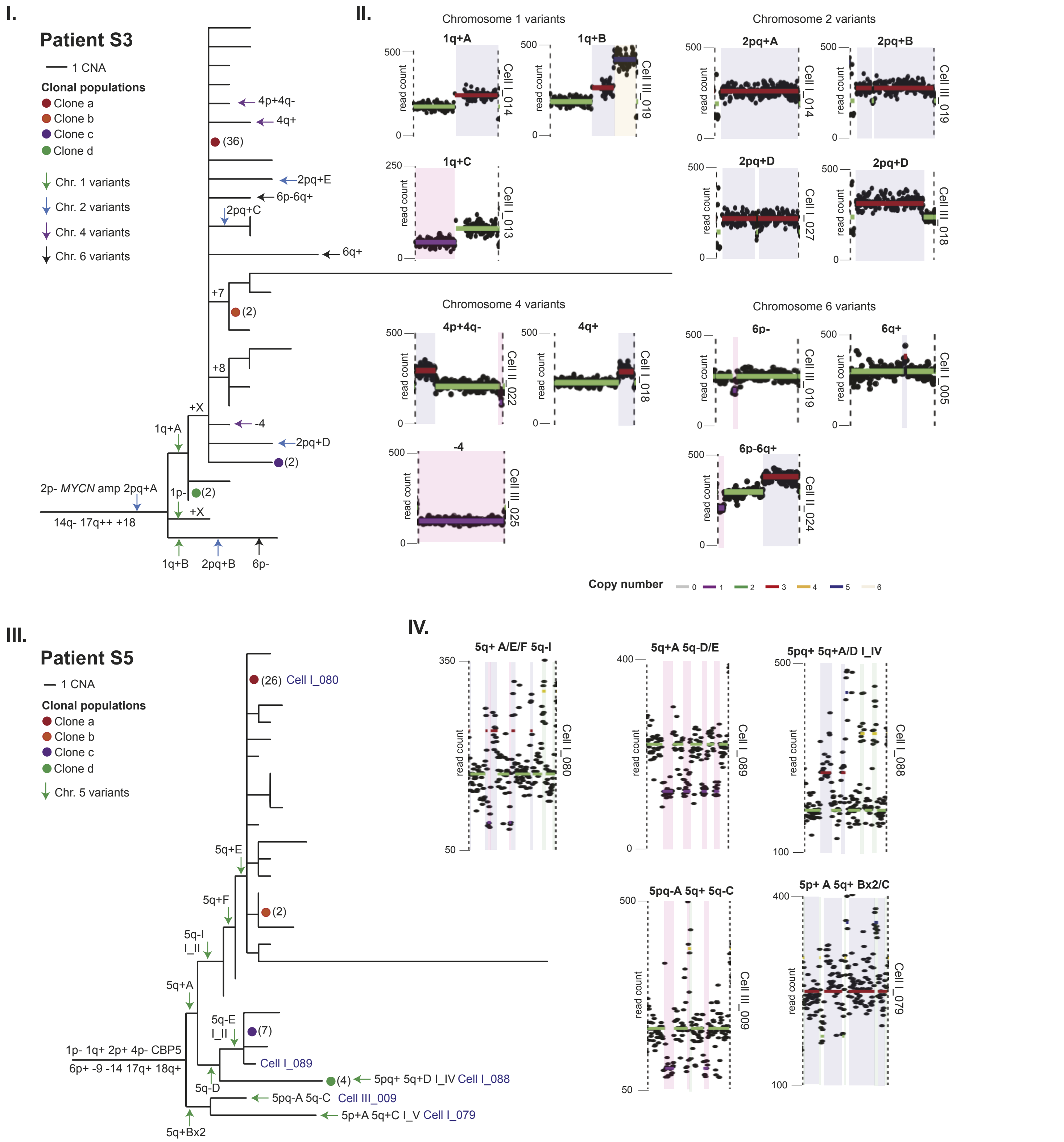
I.
Patient S3
1 CNA
Clonal populations
Clone a
Clone b
Clone c
Clone d
Chr. 1 variants
Chr. 2 variants
Chr. 4 variants
Chr. 6 variants
4p+4q-
4q+
(36)
2pq+E
6p-6q+
2pq+C
6q+
+7
(2)
+8
+X
1q+A
-4
2pq+D
(2)
2p- MYCN amp 2pq+A
1p-
(2)
+X
14q- 17q++ +18
1q+B
2pq+B
6p-
II.
Chromosome 1 variants
1q+A
1q+B
1q+C
Cell I_014
Cell III_019
Cell I_013
Chromosome 2 variants
2pq+A
2pq+B
2pq+D
2pq+D
Cell I_014
Cell III_019
Cell I_027
Cell III_018
Chromosome 4 variants
4p+4q-
4q+
-4
Cell II_022
Cell I_018
Cell III_025
Chromosome 6 variants
6p-
6q+
6p-6q+
Cell III_019
Cell I_005
Cell II_024
read count
Copy number
III.
Patient S5
1 CNA
Clonal populations
Clone a
Clone b
Clone c
Clone d
Chr. 5 variants
(26) Cell I_080
5q+E
5q+F
(2)
5q-I I_II
5q+A
5q-E I_II
(7)
Cell I_089
1p- 1q+ 2p+ 4p- CBP5
6p+ -9 -14 17q+ 18q+
5q-D
(4) 5pq+ 5q+D I_IV Cell I_088
5pq-A 5q-C Cell III_009
5p+A 5q+C I_V Cell I_079
5q+Bx2
IV.
5q+ A/E/F 5q-I
5q+A 5q-D/E
5pq+ 5q+A/D I_IV
5pq-A 5q+ 5q-C
5p+ A 5q+ Bx2/C
Cell I_080
Cell I_089
Cell I_088
Cell III_009
Cell I_079
read count

Suppl Fig 7

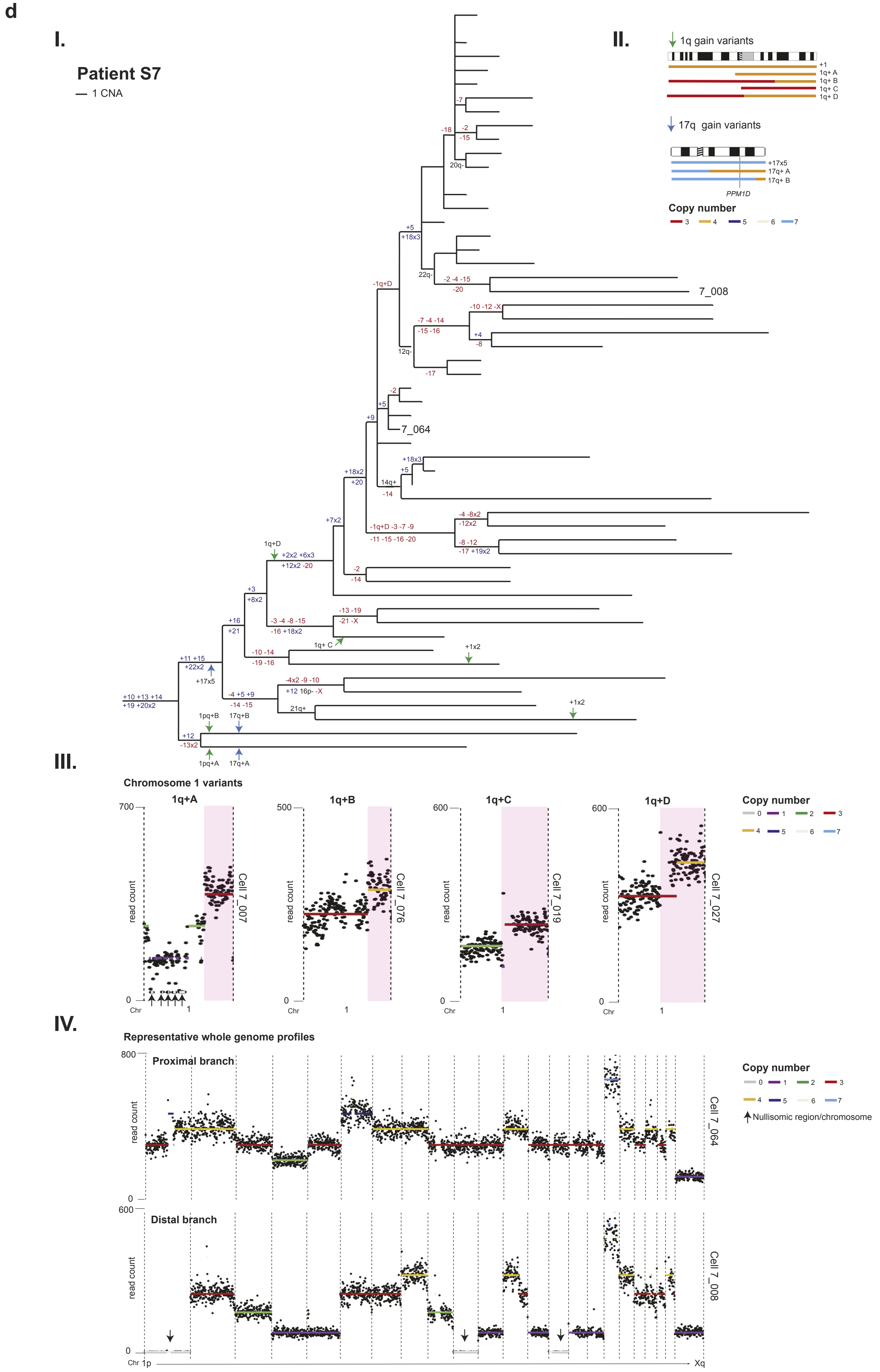
d
I.
Patient S7
1 CNA
7_008
7_064
II.
1q gain variants
+1
1q+ A
1q+ B
1q+ C
1q+ D
17q gain variants
+17x5
17q+ A
17q+ B
PPM1D
Copy number
III.
Chromosome 1 variants
1q+A
1q+B
1q+C
1q+D
Cell 7_007
Cell 7_076
Cell 7_019
Cell 7_027
read count
Chr
IV.
Representative whole genome profiles
Proximal branch
Distal branch
Cell 7_064
Cell 7_008
Nullisomic region/chromosome
Chr 1p
Xq

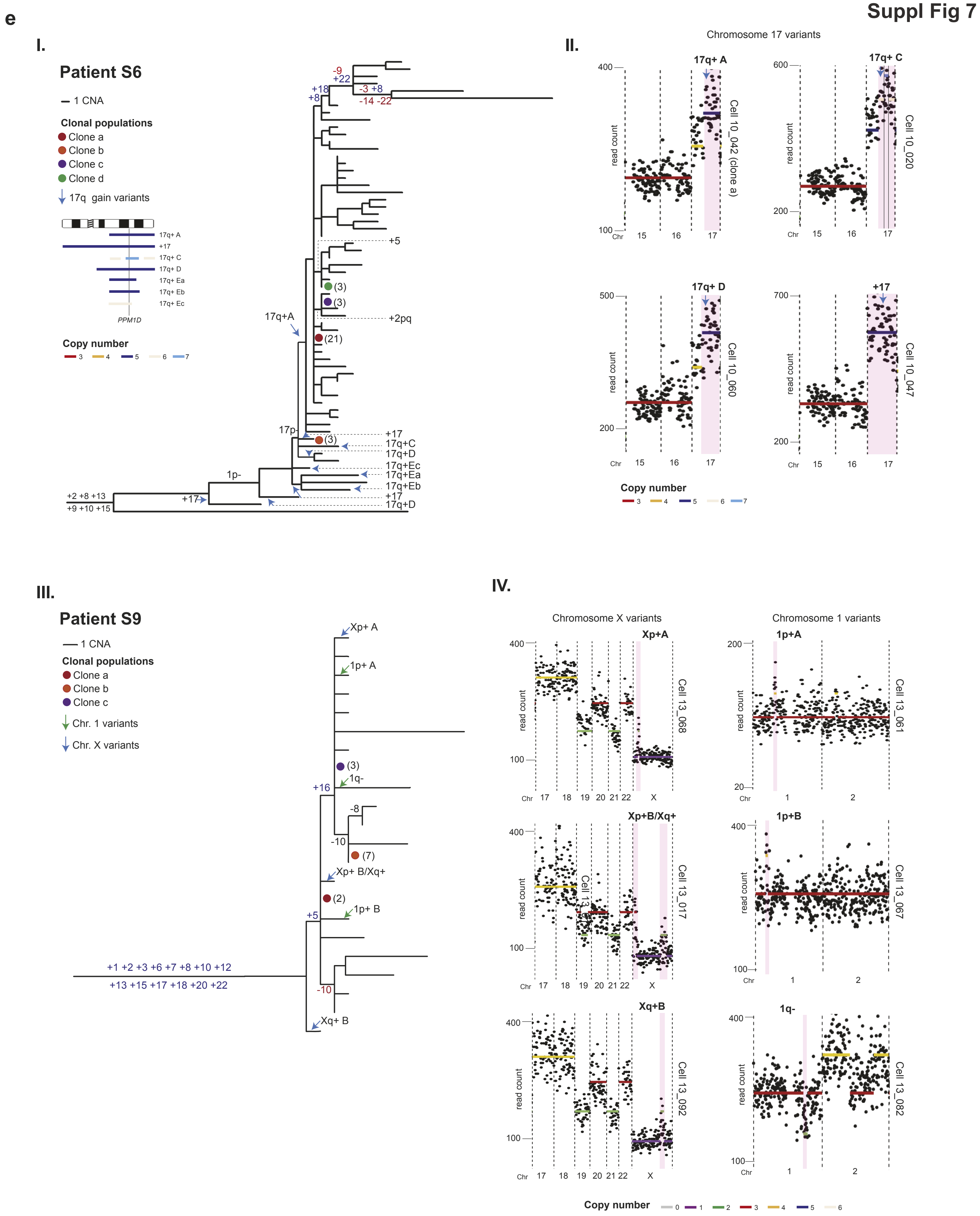
e
I.
Patient S6
1 CNA
Clonal populations
Clone a
Clone b
Clone c
Clone d
17q gain variants
17q+ A
+17
17q+ C
17q+ D
17q+ Ea
17q+ Eb
17q+ Ec
PPM1D
Copy number
3 4 5 6 7
17q+A
17p-
1p-
+17
+2 +8 +13
+9 +10 +15
+5
+2pq
(3)
(3)
(21)
(3)
17q+C
17q+D
17q+Ec
17q+Ea
17q+Eb
17q+D
-9
+22
+18
+8
-3 +8
-14 -22
II.
Chromosome 17 variants
17q+ A
Cell 10_042 (clone a)
17q+ C
Cell 10_020
17q+ D
Cell 10_060
+17
Cell 10_047
read count
Chr 15 16 17
Copy number
3 4 5 6 7
III.
Patient S9
1 CNA
Clonal populations
Clone a
Clone b
Clone c
Chr. 1 variants
Chr. X variants
Xp+ A
1p+ A
(3)
1q-
+16
-8
-10
(7)
Xp+ B/Xq+
(2)
1p+ B
+5
-10
Xq+ B
+1 +2 +3 +6 +7 +8 +10 +12
+13 +15 +17 +18 +20 +22
IV.
Chromosome X variants
Chromosome 1 variants
Xp+A
Cell 13_068
1p+A
Cell 13_061
Xp+B/Xq+
Cell 13_017
1p+B
Cell 13_067
Xq+B
Cell 13_092
1q-
Cell 13_082
read count
Chr 17 18 19 20 21 22 X
Chr 1 2
Copy number
0 1 2 3 4 5 6

f

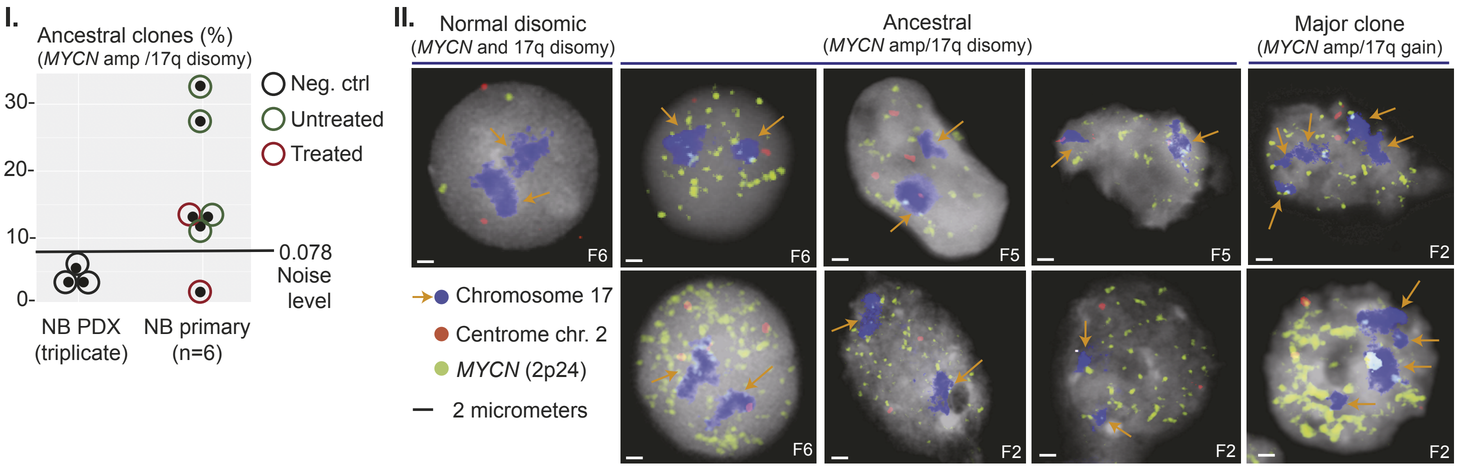
I.
Ancestral clones (%)
(MYCN amp /17q disomy)
30-
20-
10-
0-
Neg. ctrl
Untreated
Treated
0.078
Noise
level
NB PDX
(triplicate)
NB primary
(n=6)
II.
Normal disomic
(MYCN and 17q disomy)
Ancestral
(MYCN amp/17q disomy)
Major clone
(MYCN amp/17q gain)
F6
F6
F5
F5
F2
F6
F2
F2
F2
Chromosome 17
Centrome chr. 2
MYCN (2p24)
2 micrometers

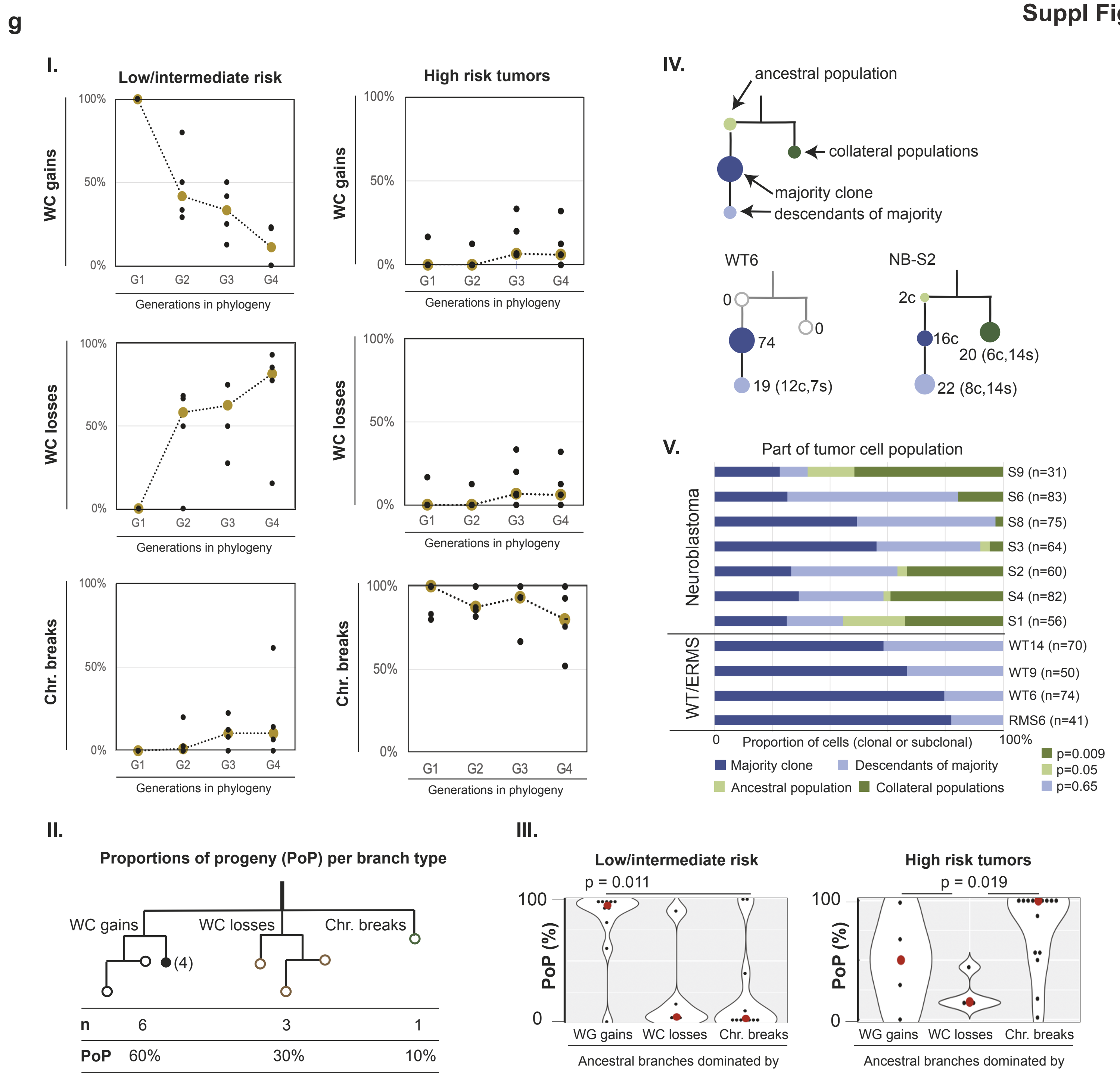
g
I.
Low/intermediate risk
High risk tumors
WC gains
WC losses
Chr. breaks
100%
50%
0%
G1
G2
G3
G4
Generations in phylogeny
II.
Proportions of progeny (PoP) per branch type
WC gains
WC losses
Chr. breaks
(4)
n 6 3 1
PoP 60% 30% 10%
III.
Low/intermediate risk
High risk tumors
p = 0.011
p = 0.019
PoP (%)
100
0
WG gains
WC losses
Chr. breaks
Ancestral branches dominated by
IV.
ancestral population
collateral populations
majority clone
descendants of majority
WT6
NB-S2
0
0
74
19 (12c,7s)
2c
16c
20 (6c,14s)
22 (8c,14s)
V.
Part of tumor cell population
Neuroblastoma
WT/ERMS
S9 (n=31)
S6 (n=83)
S8 (n=75)
S3 (n=64)
S2 (n=60)
S4 (n=82)
S1 (n=56)
WT14 (n=70)
WT9 (n=50)
WT6 (n=74)
RMS6 (n=41)
0
Proportion of cells (clonal or subclonal)
100%
Majority clone
Descendants of majority
Ancestral population
Collateral populations
p=0.009
p=0.05
p=0.65

Supplementary Figure 7. Evolutionary history at the single cell level. a:I. Evolution from ancestral *MYCN* and *MAML3* copy number gain in Patient S2. Single cell copy number profiles based on whole genome sequencing (WGS) from four representative cells showing transition from low copy-number gain of *MYCN* and *MAML3* (Cell 4_079) in absence of other detectable CNAs, through higher copy-number gain/amplification (Cell 4_084), followed by loss of 1p (Cell 4_003) and finally gain of 17q (Cell 4_025) in a major population (clones a-c in panel III). Figure annotations are as in Fig. 7. **a:II**. *MYCN* and *MAML3* region breakpoint clusters (BPC) from the same case. Here, *MYCN* amplicon structure was homogeneous with a single type of cassette (A) detected, while there was a range of *MAML3* amplicons, including a set of primordial amplicons (P), amplicon cassette A framed by BPC #14 and 17# and amplicon C in which the additional BPCs #15 and #16 are observed. **a:III.** Single cell phylogeny of the diagnostic biopsy of the same tumor taking into account *MYCN* and *MAML3* BPCs as well as other detected CNAs. Early ancestral branching manifests as diversity in breakpoints around *MAML3*, followed by clonal expansion (Clones a-c) once deletions in 1p and 11q as well as 17q gain have been acquired. There is also evidence of parallel evolution (colored arrows), i.e the emergence of variant chromosome structures, having different breakpoints, but leading to loss or gain of overlapping segments in 1p, 9q, 17q and 20q. Parallel events in 20q (green arrows) separate the main clonal populations. **a:IV**. Examples of parallel breakpoints detected as shifts in copy numbers along the chromosomal arms. The resulting CNAs are in regions with previously reported putative neuroblastoma drivers as annotated at the bottom of the figure,[1-6] where also the locations of CNA variants across clones (a-c) or single cells are denoted (bars colored according to copy number). **a:V.** *MYCN* (left) and *MAML3* copy numbers assessed by single cell WGS in Patient S2 across the main clonal populations (a-c) and the ancestral population (P) having only *MYCN* and *MAML* gain but lacking 1p-, 11q- and 17q+. Red circles represent median; significance testing by Mann Whitney U-test (two-sided) using Benjamini-Hochberg correction for multiple comparisons. Note a significant increase in *MYCN* and *MAML3* copy numbers with evolution from P to a-c. **b:I.** Maximum likelihood phylogeny of a non-*MYCN* amplified high-risk neuroblastoma (Patient S4) based on single cell copy number profiling. Stem anomalies including 11q deletion (11q-) and 17q gain (17q +) (present in all tumor cells) are followed by branching evolution through deletions with distinct breakpoints in 3p (3p- A-C; blue arrows). Three cells (e.g cell 1_029; black arrowheads) also accumulate multiple whole-chromosome aberrations (aneuploidy), indicating features of saltatory evolution. The number of cells in each clone is given in parentheses. **b:II**. Chromosome 3 breakpoints corresponding to parallel early branches in b:I. **b:III**. Representative CNA profile of the main clone (a) in B:I, in comparison to the highly aneuploid cell 1_029. Stem anomalies are in purple type. **b:IV**. Maximum likelihood phylogeny of a low-risk neuroblastoma (Patient S8) based on single cell copy number profiling. Stem anomalies including multiple whole chromosome gains are followed by early branching through alterations in chromosome 6, including a trisomy (+6) and two distinct deletions (6q- and 6p-). More distal branching is largely contingent on breaks in chromosome 1 and 17. **b:V**. Representative examples of distinct breakpoints in chromosome 6 and 1, leading to branching evolution in b:IV. **c:I.** Maximum likelihood phylogeny of a *MYCN*-amplified high-risk neuroblastoma (Patient S3) based on single cell copy number profiling. Stem anomalies include *MYCN* amplification, other structural alterations of chromosome 2, a deletion in the long arm of chromosome 14 (14q-), gain of two copies of 17q (17q++), and trisomy 18 (+18). Subsequent branching into the main clonal populations (a-d) is largely contingent on structural alterations of a subset of chromosomes (colored arrows) including chromosome 1, 2, 4 and 6. Filled circles represent clonal populations with the number of cells in parenthesis. **c:II.** Examples of parallel breakpoints detected as shifts in copy numbers along the chromosomal arms of a subset of chromosomes. Copy number (colored lines; key at bottom if panel) is estimated by the number of reads at whole genome sequencing. **c:III**.

Maximum likelihood phylogeny of a non-*MYCN* amplified high-risk neuroblastoma (Patient S5) based on single cell copy number profiling. The stem anomalies include multiple structural alterations including 1p loss (1p-), 17q gain (17q+) and complex chromothripsis-like breakpoints (CBP) in chromosome 5. Further evolution is initiated by branching through further rearrangement of chromosome 5. **c:IV**. Examples of chromosome 5 variants observed in different cells, where A-F represent different segments gained or lost. **d:I.** Maximum likelihood phylogeny of an intermediate risk neuroblastoma (Patient S7) based on single cell copy number profiling. Stem anomalies include gain of one or two (x2) copies of multiple whole chromosomes. Further evolution occurs by gain and loss of whole chromosomes as well as structural rearrangements because of chromosome breaks, including multiple breakpoints in chromosomes 1 and 17 in parallel lineages (green and blue arrows). While most early alterations are whole chromosome gains (blue type), late branching occurs largely because of whole chromosome losses (red type). There were no clonal populations detected, i.e. each cell had a unique CNA profile. **d:II.** Chromosome ideogram maps of parallel gains in chromosome arms 1q and 17q, defined by unique breakpoints A-D and A-B, respectively. **d:III.** Examples of chromosome arm 1q variant breakpoints observed in different cells. **d:IV.** Whole genome profiles representing proximal (early) and distal (late) branches of the phylogeny, respectively, where the former has a domination of whole chromosome gains while the latter has accumulated multiple losses, including absence of several whole chromosomes (nullisomies, arrows). **e:I.** Maximum likelihood phylogeny of an intermediate risk neuroblastoma (Patient S6) based on single cell copy number profiling. Stem anomalies include gains of multiple whole chromosomes, while subsequent branching occurs mainly through structural variation of chromosome 17 (green arrows), where variant 17q+A is ancestral to most clonal populations (a, c, d; number of cells per clone in parentheses). Inset clarifies breakpoints of different variants A-E; variants Ea-Ec share a proximal breakpoint but have distinct distal breakpoints. **e:II**. Examples of chromosome arm 17q gain occurring through different breakpoints (A, C, D) or through trisomy 17 (+17) in different cells. Copy number profiles of chromosomes 15 and 16 are shown as a reference. **e:III**. Maximum likelihood phylogeny of an intermediate-risk neuroblastoma (Patient S9). Stem anomalies include gain of one or two (x2) copies of multiple whole chromosomes. Further evolution occurs predominantly by additional whole chromosome gains and structural rearrangements involving chromosomes 1 and X (green and blue arrows). **e:IV**. Breakpoint variants in chromosomes 1 and X, with profiles of other chromosomes shown as a reference. **f:I.** Detection of ancestral clones by fluorescence in situ hybridization (FISH). Proportion of neuroblastoma cells (ascertained by gain/amplification of *MYCN*) showing disomic 17q status as detected by FISH. Cultured PDX cells where no ancestral population is retained according to single cell WGS are used as a negative control to set a background level of 17q disomy by analysis of a technical triplicate. The noise level is calculated as the geometric mean+2 standard deviations. In NB primary tumor material 5/6 tumors showed a frequency above the background level of MYCN-amplified cells with retained disomic 17q status. **f:II**. Representative FISH-images with probes directed against the *MYCN* region (green), centromere 2 (red) and chromosome 17 (blue). Orange arrows denote nuclear domains occupied by chromosome 17 material. **g:I.** Timing of aberrations along evolutionary history assessed by single cell WGS. Relative frequencies of whole chromosome (WC) gains, WC losses and chromosome (Chr.) breaks (partial deletions, duplications) across generations (G) 1 through 4 in copy number-based single cell phylogenies from high- (n=5) and low/intermediate risk (n=4) neuroblastomas, respectively. In low/intermediate risk cases, there is a significant decrease of WC gain across successive generations ($p<0.0001$; Chi-Square test) with a parallel increase in chromosome losses and breaks. In high-risk cases, the relative proportions of these aberrations remain stable across generations, with a maintained high proportion of chromosome breaks. Single data points are represented by black dots, medians

by brown circles connected by a stippled trend line. **g:II.** Principle for calculation of the proportion of progeny (PoP) per branch, resulting from branches dominated by WC gains, WC losses and chromosomal breaks (structural rearrangements). The number of descendant cells from each branch (n) is set to arbitrary values to illustrate a hypothetical population of 10 analyzed cells. **g:III**. PoP values for WC gains, WC losses, and chromosomal breaks across generations 2-4 from low/intermediate risk (n=4) and high-risk (n=5) neuroblastomas. Significance testing by Mann Whitney U-test (two-sided) using Benjamini-Hochberg correction for multiple comparisons. **g:IV.** Four phylogenetically distinct tumor cell populations whose proportions are compared to assess whether there is early or late branching (top ideogram). Late branching is exemplified by a Wilms tumor (WT6; left lower subpanel) where only the largest clone and its descendants are detected. Early branching is exemplified by a neuroblastoma (NB-S2; right lower subpanel), where there are detectable populations ancestral and collateral to the largest clone; c signifies cells in clones, s cells detected as single unique genotypes. **g:V.** Relative proportions of cells (n= number of cells sequenced) belonging to the different phylogenetic classes described in g:IV in neuroblastoma compared to a published control group of Wilms tumors and embryonal rhabdomyosarcomas (WT/ERMS);[7] neuroblastoma Patient S7 did not have any clonal populations and was not included in the analysis. The detectable proportion of cells collaterally related to the largest clone is larger in neuroblastomas than in WT/RMS (p-values in figure by Mann-Whitney U-test, two-sided, using Benjamini-Hochberg correction for multiple comparisons). Source data are provided as a Source Data file.

a. Patient 1 (2n)

Suppl Fig 8

I. VAFs

	Pre1	Pre2	Pre3	Pre4	MP1	MP2	MP3	MP4
1:159901733	0,5	0,3	0,4	0,6	0,4	0,3	0,1	0,1
10:79613219	0,5	0,5	0,5	0,5	0,4	0,2	0,2	0,1
12:7361601	0,4	0,5	0,5	0,5	0,3	0,3	0,1	0,1
3:40528900	0,4	0,4	0,5	0,5	0,3	0,4	0,2	0,1
17:7736407	0,5	0,4	0,4	0,5	0,4	0,2	0,2	0,1
X:150911102	1,0	1,0	0,9	0,8	0,9	0,5	0,2	0,1
14:24113619	0,4	0,5	0,5	0,4	0,3	0,2	0,2	0,1
5:35047956	0,3	0,3	0,3	0,3	0,3	0,2	0,2	0,1
19:58565182	0,3	0,6	0,5	0,4	0,3	0,3	0,2	0,1
17:10438615	0,5	0,4	0,4	0,4	0,4	0,3	0,1	0,1
22:51160216	0,5	0,5	0,3	0,4	0,3	0,2	0,3	0,1
15:23261896	0,4	0,3	0,5	0,4	0,4	0,2	0,1	0,0
6:29394620	0,3	0,2	0,3	0,2	0,5	0,3	0,2	0,1
16:68155990	0,1	0,1	0,2	0,2	0,3	0,3	0,2	0,1
3:52012313	0,1	0,1	0,1	0,1	0,4	0,3	0,1	0,1
19:7927142	0,1	0,1	0,1	0,0	0,4	0,4	0,3	0,1
4:187115755	0,3	0,4	0,5	0,5	0,0	0,0	0,0	0,0
7:128475431	0,2	0,4	0,4	0,4	0,0	0,0	0,0	0,0
15:80884025	0,2	0,4	0,4	0,4	0,0	0,0	0,0	0,0
16:846823	0,3	0,3	0,3	0,4	0,0	0,0	0,0	0,0
19:22497622	0,3	0,2	0,4	0,2	0,0	0,0	0,0	0,0
6:46656489	0,3	0,3	0,2	0,2	0,0	0,0	0,0	0,0
16:75147997	0,0	0,0	0,0	0,0	0,4	0,2	0,2	0,0
19:3964253	0,0	0,0	0,0	0,0	0,4	0,3	0,1	0,0
5:23522841	0,0	0,0	0,0	0,0	0,2	0,2	0,0	0,0
19:7964898	0,0	0,0	0,0	0,0	0,5	0,2	0,1	0,0
6:35446217	0,0	0,0	0,0	0,0	0,3	0,2	0,0	0,0

II. Original clone sizes

	Pre1	Pre2	Pre3	Pre4	MP1	MP2	MP3	MP4	subclone
1:159901733	1,0	0,7	0,9	1,2	1,1	1,1	0,8	0,8	stem
10:79613219	1,1	1,0	1,0	1,1	1,2	1,0	1,0	1,0	stem
12:7361601	1,0	1,2	1,1	1,1	0,9	1,1	0,8	1,4	stem
3:40528900	0,9	1,0	1,2	1,0	0,8	1,2	1,3	0,9	stem
17:7736407	1,0	1,0	0,9	1,0	1,2	0,8	1,0	1,0	stem
X:150911102	1,0	1,1	1,0	0,9	1,2	1,0	0,4	0,9	stem
14:24113619	0,9	1,2	1,1	1,0	0,9	0,8	1,3	1,4	stem
5:35047956	1,0	0,9	0,8	0,9	1,2	1,0	1,4	1,2	stem
19:58565182	0,8	1,3	1,1	1,0	0,9	1,0	1,2	1,1	stem
17:10438615	1,2	1,0	0,9	0,9	1,1	1,2	0,3	1,6	stem
22:51160216	1,1	1,0	0,8	0,9	0,9	0,8	1,7	1,0	stem
15:23261896	0,9	0,8	1,0	0,9	1,1	0,9	0,8	0,5	stem
6:29394620	0,4	0,3	0,3	0,3	1,3	1,1	1,3	0,8	D
16:68155990	0,3	0,2	0,4	0,3	1,0	1,0	1,0	0,7	D
3:52012313	0,2	0,3	0,2	0,3	1,1	1,2	0,8	1,0	D
19:7927142	0,3	0,3	0,2	0,1	1,2	1,4	1,5	0,9	D
4:187115755	0,8	1,0	0,7	0,8	0,0	0,0	0,0	0,0	C
7:128475431	0,5	0,8	0,8	0,9	0,0	0,0	0,0	0,0	C
15:80884025	0,5	0,9	1,0	0,8	0,0	0,0	0,0	0,0	C
16:846823	0,7	0,6	0,7	0,8	0,0	0,0	0,0	0,0	C
19:22497622	0,5	0,4	0,9	0,5	0,0	0,0	0,0	0,0	C
6:46656489	0,3	0,3	0,3	0,2	0,0	0,0	0,0	0,0	E
16:75147997	0,0	0,0	0,0	0,0	1,2	0,9	1,2	0,0	B
19:3964253	0,0	0,0	0,0	0,0	1,0	1,0	0,8	0,0	B
5:23522841	0,0	0,0	0,0	0,0	0,8	1,1	0,0	0,0	A
19:7964898	0,0	0,0	0,0	0,0	0,8	0,9	0,0	0,0	A
6:35446217	0,0	0,0	0,0	0,0	1,3	0,7	0,0	0,0	A

III. Clone sizes calculated with DeCiFer

	Pre1	Pre2	Pre3	Pre4	MP1	MP2	MP3	MP4
1:159901733	1,0	0,7	1,0	1,3	1,1	1,0	0,8	0,8
10:79613219	1,1	1,0	1,1	1,1	1,2	0,9	1,0	0,9
12:7361601	1,0	1,2	1,2	1,1	0,9	1,0	0,8	1,3
3:40528900	0,9	1,0	1,2	1,1	0,9	0,8	1,3	0,8
17:7736407	1,0	1,0	1,0	1,0	1,2	0,7	1,0	0,9
X:150911102	1,0	1,1	1,1	0,9	1,2	0,9	0,9	0,8
14:24113619	0,9	1,2	1,1	1,0	0,9	0,8	1,3	1,3
5:35047956	1,0	1,0	0,9	1,0	1,4	1,2	0,9	0,8
19:58565182	0,8	1,3	1,2	1,0	0,9	1,0	1,2	1,0
17:10438615	1,2	1,0	0,9	0,9	1,1	1,1	0,3	0,8
22:51160216	1,1	1,0	0,8	0,9	0,9	0,7	0,8	0,9
15:23261896	0,9	0,8	1,0	0,9	1,1	0,9	0,8	0,5
6:29394620	0,7	0,5	0,7	0,6	1,3	1,0	1,3	0,8
16:68155990	0,3	0,2	0,4	0,3	1,0	1,0	1,0	0,7
3:52012313	0,2	0,3	0,2	0,3	1,1	1,1	0,8	0,9
19:7927142	0,3	0,3	0,2	0,1	1,2	1,4	0,8	0,8
4:187115755	0,8	1,0	1,4	1,4	0,0	0,0	0,0	0,0
7:128475431	0,5	0,8	0,9	0,9	0,0	0,0	0,0	0,0
15:80884025	0,5	0,9	1,0	0,8	0,0	0,0	0,0	0,1
16:846823	0,7	0,6	0,8	0,8	0,0	0,0	0,0	0,1
19:22497622	0,5	0,4	0,9	0,5	0,0	0,0	0,0	0,0
6:46656489	0,6	0,7	0,6	0,5	0,0	0,0	0,0	0,0
16:75147997	0,0	0,0	0,0	0,0	1,2	0,8	1,2	0,0
19:3964253	0,0	0,0	0,0	0,1	1,0	1,0	0,8	0,1
5:23522841	0,0	0,0	0,0	0,0	0,9	1,2	0,0	0,1
19:7964898	0,0	0,0	0,0	0,0	1,3	0,6	0,3	0,0
6:35446217	0,0	0,0	0,0	0,0	0,8	0,9	0,0	0,1

IV. Phylogenetic tree based on original data

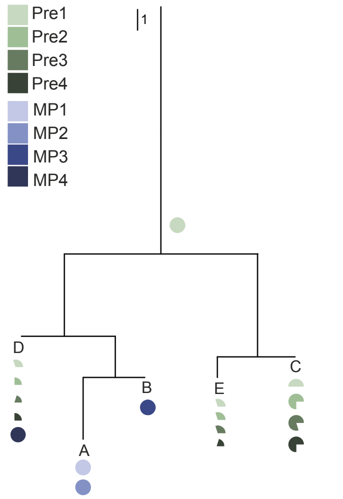

V.

SNVs originally detected	27
SNVs on CNAs without probability score in DeCiFer	0
SNVs not assigned to a cluster by DeCiFer	17

b. Patient 3 (5n)

Suppl Fig 8

I.

VAFs

	Pre1	Pre2	Pre3	Post1	Post2	Post3	Post4	
22:50485645	0,4	0,4	0,3	0,0	0,2	0,0	0,0	▲
19:51885796	0,0	0,0	0,0	0,2	0,0	0,4	0,4	▲
2:86998753	0,0	0,0	0,0	0,2	0,0	0,2	0,2	▲
10:99342123	0,0	0,0	0,1	0,0	0,0	0,0	0,0	▲

II.

Original clone sizes

	Pre1	Pre2	Pre3	Post1	Post2	Post3	Post4	
22:50485645	1,2	1,1	0,9	0,0	0,7	0,0	0,0	B
19:51885796	0,0	0,0	0,0	0,7	0,0	1,1	1,1	A
2:86998753	0,0	0,0	0,0	0,7	0,0	0,9	1,1	A
10:99342123	0,0	0,0	0,2	0,0	0,0	0,0	0,0	C

III.

Clone sizes calculated with DeCiFer

- DeCiFer analysis not applicable

IV.

Phylogenetic tree based on original data

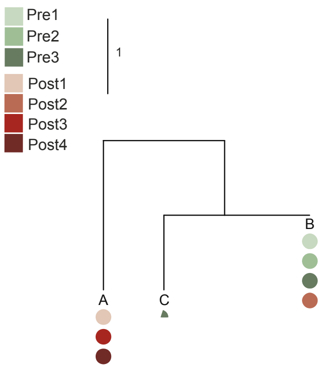

V.

SNVs originally detected	4
SNVs on CNAs without probability score in DeCiFer	4
SNVs not assigned to a cluster by DeCiFer	-

c. Patient 4 (2n)

Suppl Fig 8

I. VAFs

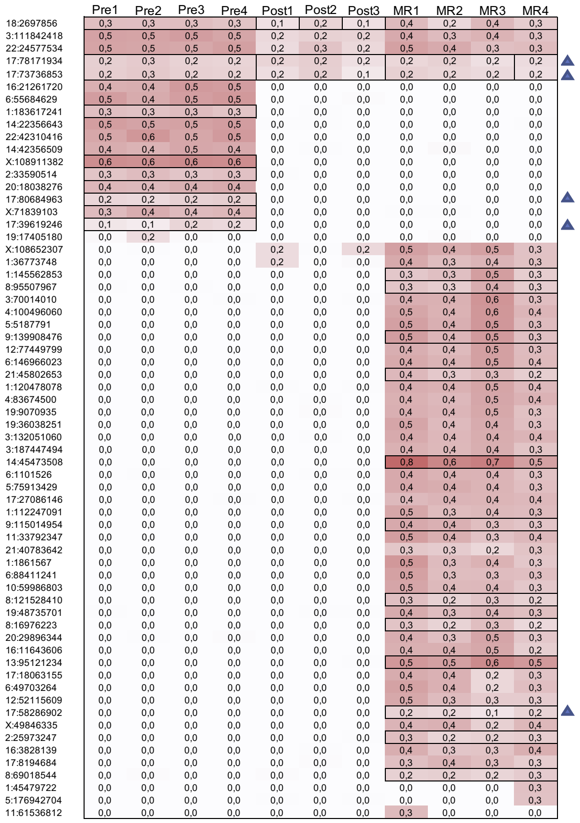

II. Original clone sizes

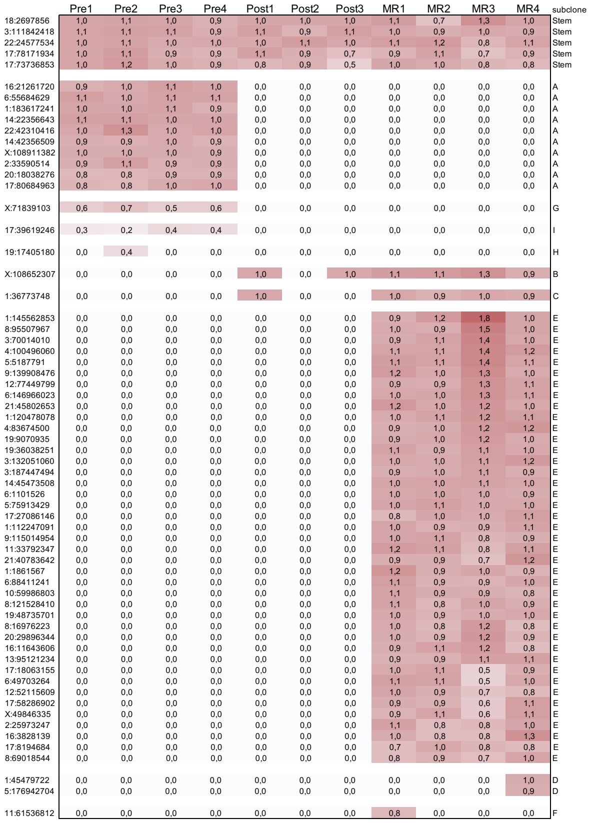

III. Clone sizes calculated with DeCiFer

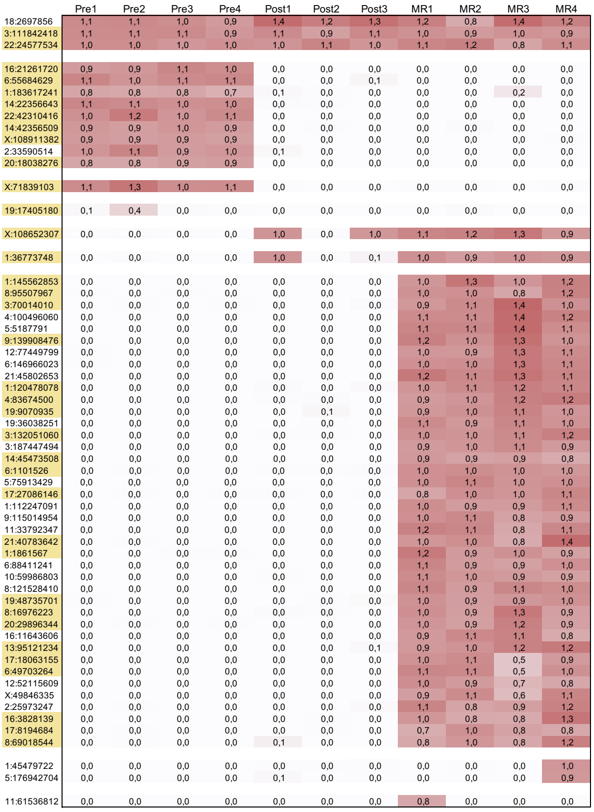

IV. Phylogenetic tree based on original data

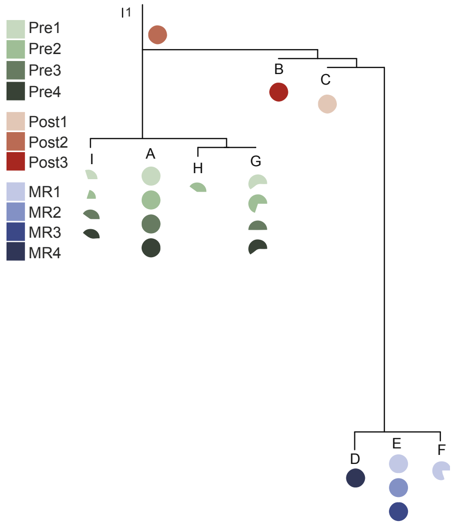

V.

SNVs originally detected	64
SNVs on CNAs without probability score in DeCiFer	5
SNVs not assigned to a cluster by DeCiFer	36

d. Patient 5 (4n)

I. VAFs

	Pre1	Pre2	Pre3	Pre4	Post2
21:43412638	0,5	0,4	0,5	0,4	0,3
12:27556449	0,5	0,5	0,4	0,5	0,3
4:100203534	0,5	0,4	0,4	0,5	0,3
7:155755666	0,5	0,5	0,5	0,5	0,3
2:11706620	0,5	0,5	0,4	0,5	0,3
4:114117664	0,5	0,5	0,5	0,5	0,3
1:186916019	0,5	0,4	0,4	0,5	0,4
1:216420555	0,5	0,4	0,4	0,5	0,5
2:200245224	0,5	0,4	0,5	0,5	0,3
10:96540413	0,5	0,4	0,5	0,4	0,3
6:30608336	0,5	0,4	0,4	0,4	0,3
19:44739802	0,5	0,4	0,4	0,4	0,3
21:31720886	0,5	0,5	0,5	0,5	0,3
17:38507731	0,3	0,3	0,3	0,3	0,3
5:34757407	0,5	0,5	0,4	0,4	0,3
6:112382239	0,5	0,4	0,4	0,5	0,4
18:48439269	0,5	0,4	0,4	0,5	0,3
17:38319778	0,3	0,3	0,3	0,3	0,3
2:179477642	0,4	0,5	0,4	0,4	0,3
3:156877741	0,4	0,5	0,4	0,5	0,3
1:94495972	0,4	0,4	0,4	0,5	0,3
6:26508738	0,4	0,5	0,5	0,4	0,3
2:219922434	0,4	0,5	0,5	0,5	0,3
19:52147181	0,4	0,5	0,5	0,5	0,2
9:87635188	0,4	0,4	0,4	0,4	0,3
19:6213764	0,4	0,5	0,4	0,5	0,3
3:197463616	0,4	0,4	0,5	0,5	0,4
22:32439414	0,4	0,5	0,5	0,5	0,3
14:65241268	0,4	0,4	0,5	0,5	0,3
4:183090383	0,5	0,5	0,5	0,5	0,2
3:49282215	0,5	0,4	0,5	0,5	0,3
7:75182877	0,4	0,5	0,4	0,5	0,2
22:24509680	0,4	0,5	0,5	0,4	0,2
1:152327823	0,4	0,4	0,4	0,4	0,4
21:43522324	0,4	0,4	0,4	0,4	0,2
19:51518765	0,4	0,5	0,4	0,5	0,2
10:13361313	0,4	0,5	0,5	0,5	0,3
19:45853933	0,4	0,4	0,4	0,4	0,2
20:34243186	0,5	0,5	0,6	0,6	0,2
1:158911889	0,4	0,5	0,4	0,5	0,1
15:40574518	0,5	0,5	0,5	0,6	0,1
4:187517847	0,5	0,4	0,4	0,5	0,2
3:172835306	0,4	0,4	0,5	0,4	0,2
8:22472163	0,4	0,3	0,3	0,2	0,3
3:69168802	0,4	0,3	0,3	0,2	0,3
X:107821279	0,8	0,6	0,6	0,5	0,1
18:46798712	0,4	0,3	0,3	0,2	0,0
15:43749005	0,4	0,3	0,4	0,3	0,0
22:37466621	0,5	0,3	0,3	0,2	0,0
8:87162450	0,4	0,3	0,3	0,2	0,0
14:96135938	0,4	0,3	0,3	0,2	0,0
18:47801624	0,4	0,3	0,3	0,2	0,0
16:29439723	0,4	0,3	0,3	0,2	0,0
22:40080460	0,4	0,3	0,3	0,2	0,0
5:143346	0,3	0,2	0,2	0,2	0,0
19:33687669	0,4	0,3	0,3	0,2	0,0
16:57687854	0,4	0,3	0,3	0,2	0,0
8:73982064	0,4	0,3	0,3	0,2	0,0
22:30973069	0,0	0,3	0,2	0,2	0,0
16:24828143	0,1	0,3	0,2	0,2	0,0
X:153218358	0,0	0,6	0,5	0,5	0,0
2:97474358	0,0	0,3	0,2	0,2	0,0
X:151869805	0,1	0,6	0,4	0,4	0,0
4:123229238	0,0	0,3	0,2	0,2	0,0
15:42982905	0,0	0,3	0,2	0,3	0,0
15:43767792	0,0	0,2	0,2	0,3	0,0
20:20013273	0,0	0,1	0,1	0,2	0,0
3:132390692	0,0	0,2	0,1	0,3	0,0
2:228147100	0,1	0,1	0,1	0,2	0,0
6:99857064	0,1	0,1	0,1	0,2	0,0
19:54261563	0,4	0,0	0,1	0,0	0,0
20:6011976	0,4	0,0	0,1	0,0	0,0
X:77913206	0,7	0,0	0,1	0,0	0,0
17:77078834	0,3	0,0	0,1	0,0	0,0
13:52972327	0,4	0,0	0,1	0,0	0,0
5:110097136	0,4	0,0	0,1	0,0	0,0
1:167343434	0,4	0,0	0,1	0,0	0,0
4:1326650	0,4	0,0	0,1	0,0	0,0
20:52602035	0,3	0,0	0,1	0,0	0,0
14:103418903	0,3	0,0	0,1	0,0	0,0
10:118616136	0,2	0,0	0,0	0,0	0,0

II. Original clone sizes

	Pre1	Pre2	Pre3	Pre4	Post2	subclo
21:43412638	1,2	1,0	1,1	1,0	1,2	Stem
12:27556449	1,2	1,1	1,0	1,0	1,1	Stem
4:100203534	1,2	1,0	1,0	1,0	1,1	Stem
7:155755666	1,1	1,2	1,0	1,0	1,0	Stem
2:11706620	1,1	1,1	1,0	1,1	1,1	Stem
4:114117664	1,1	1,1	1,1	1,0	1,1	Stem
1:186916019	1,1	1,0	1,0	1,1	1,0	Stem
1:216420555	1,1	1,0	1,0	1,0	1,0	Stem
2:200245224	1,1	1,0	1,0	1,0	1,1	Stem
10:96540413	1,1	1,0	1,1	0,9	1,1	Stem
6:30608336	1,1	1,0	1,0	1,0	1,1	Stem
19:44739802	1,1	1,0	1,0	1,0	1,1	Stem
21:31720886	1,1	1,1	1,1	1,0	1,0	Stem
17:38507731	1,1	1,0	1,1	1,0	1,0	Stem
5:34757407	1,1	1,1	1,0	1,0	1,0	Stem
6:112382239	1,1	1,0	1,0	1,1	1,0	Stem
18:48439269	1,0	0,9	1,0	1,2	1,3	Stem
17:38319778	1,0	1,1	1,0	1,0	1,0	Stem
2:179477642	1,0	1,1	1,0	1,0	1,1	Stem
3:156877741	1,0	1,1	1,0	1,1	0,9	Stem
1:94495972	1,0	1,0	1,0	1,0	1,0	Stem
6:26508738	1,0	1,1	1,1	1,0	1,0	Stem
2:219922434	1,0	1,1	1,1	1,0	1,2	Stem
19:52147181	1,0	1,1	1,1	1,0	1,0	Stem
9:87635188	1,0	1,0	1,0	1,1	1,0	Stem
19:6213764	1,0	1,1	1,0	1,1	1,1	Stem
3:197463616	1,0	0,9	1,1	1,0	1,1	Stem
22:32439414	1,0	1,1	1,1	1,0	1,0	Stem
14:65241268	0,9	1,0	1,1	1,1	1,1	Stem
4:183090383	1,1	1,1	1,1	1,1	1,0	Stem
3:49282215	1,1	1,0	1,1	1,0	1,1	Stem
7:75182877	1,0	1,1	1,0	1,0	1,2	Stem
22:24509680	1,0	1,1	1,1	1,0	0,9	Stem
1:152327823	1,0	1,0	1,0	1,0	0,9	Stem
21:43522324	1,0	1,0	1,0	0,9	0,9	Stem
19:51518765	1,0	1,1	1,0	1,0	0,9	Stem
10:13361313	1,0	1,1	1,0	1,1	0,9	Stem
19:45853933	0,9	1,0	1,0	0,9	0,9	Stem
20:34243186	1,1	1,0	1,1	1,0	0,9	Stem
1:158911889	1,0	1,1	1,0	1,0	0,9	Stem
15:40574518	1,2	1,1	0,9	1,0	0,9	Stem
4:187517847	1,1	1,0	0,9	1,0	0,9	Stem
3:172835306	1,0	0,9	1,1	1,0	0,9	Stem
8:22472163	0,9	0,7	0,8	0,5	1,0	A
3:69168802	0,8	0,7	0,7	0,5	0,9	A
X:107821279	1,0	0,8	0,7	0,6	0,2	G
18:46798712	0,9	0,7	0,6	0,4	0,2	G
15:43749005	1,0	0,7	0,7	0,5	0,0	B
22:37466621	1,1	0,8	0,7	0,5	0,0	B
8:87162450	1,0	0,8	0,7	0,5	0,0	B
14:96135938	1,0	0,7	0,7	0,4	0,0	B
18:47801624	1,0	0,7	0,7	0,4	0,0	B
16:29439723	1,0	0,7	0,7	0,5	0,0	B
22:40080460	0,9	0,7	0,7	0,5	0,0	B
5:143346	0,9	0,7	0,7	0,5	0,0	B
19:33687669	0,9	0,8	0,7	0,5	0,0	B
16:57687854	0,9	0,7	0,8	0,5	0,0	B
8:73982064	0,8	0,7	0,7	0,5	0,0	B
22:30973069	0,1	0,7	0,4	0,5	0,0	C
16:24828143	0,1	0,7	0,4	0,5	0,0	C
X:153218358	0,0	0,8	0,6	0,6	0,0	C
2:97474358	0,0	0,7	0,5	0,5	0,0	C
X:151869805	0,1	0,8	0,5	0,5	0,0	C
4:123229238	0,1	0,6	0,5	0,5	0,0	C
15:42982905	0,0	0,7	0,4	0,4	0,0	C
15:43767792	0,0	0,3	0,4	0,5	0,0	E
20:20013273	0,1	0,3	0,4	0,5	0,0	E
3:132390692	0,1	0,4	0,3	0,6	0,0	E
2:228147100	0,1	0,3	0,3	0,6	0,0	E
6:99857064	0,1	0,3	0,3	0,5	0,0	E
19:54261563	1,0	0,0	0,2	0,0	0,0	D
20:6011976	0,9	0,0	0,2	0,0	0,0	D
X:77913206	1,0	0,0	0,1	0,0	0,0	D
17:77078834	1,0	0,0	0,2	0,0	0,0	D
13:52972327	0,9	0,0	0,2	0,0	0,0	D
5:110097136	0,9	0,0	0,1	0,0	0,0	D
1:167343434	0,9	0,0	0,2	0,0	0,0	D
4:1326650	0,9	0,0	0,1	0,0	0,0	D
20:52602035	0,7	0,0	0,2	0,0	0,0	D
14:103418903	0,8	0,0	0,2	0,0	0,0	D
10:118616136	0,4	0,0	0,1	0,0	0,0	F

III. Clone sizes calculated with DeCiFer

	Pre1	Pre2	Pre3	Pre4	Post2
21:43412638	0,9	1,2	1,2	1,1	1,2
2:11706620	0,9	1,2	1,2	1,2	1,1
2:200245224	0,8	1,1	1,2	1,1	1,1
6:30608336	1,2	1,1	1,1	1,1	1,1
19:44739802	1,2	1,1	1,1	1,1	1,1
21:31720886	1,2	1,2	1,2	1,1	1,0
5:34757407	1,2	0,9	1,1	1,1	1,1
18:48439269	1,2	1,1	1,1	0,9	1,0
2:179477642	1,2	0,9	1,1	1,1	1,1
6:26508738	1,2	1,2	1,2	1,1	1,0
2:219922434	1,2	1,2	0,8	1,1	1,2
19:52147181	1,1	1,2	1,2	1,1	1,0
19:6213764	1,1	1,2	1,1	1,2	1,1
22:32439414	1,1	0,8	1,2	1,1	1,0
14:65241268	1,0	1,1	0,8	1,2	1,1
22:24509680	1,2	1,2	1,2	1,1	0,9
21:43522324	1,1	1,2	1,1	1,0	0,9
19:51518765	1,1	0,9	1,1	1,1	0,9
19:45853933	1,1	1,1	1,1	1,0	0,9
8:22472163	1,0	0,8	0,8	1,1	1,0
X:107821279	1,0	1,0	1,0	1,1	0,6
18:46798712	1,0	0,8	1,4	1,0	0,5
22:37466621	1,2	0,9	0,8	1,0	0,2
8:87162450	1,2	0,9	0,8	1,1	0,2
14:96135938	1,2	0,8	0,8	1,0	0,2
18:47801624	1,1	0,8	0,8	0,9	0,2
16:29439723	1,1	0,9	1,5	1,1	0,2
22:40080460	1,1	0,8	0,8	1,1	0,3
19:33687669	1,0	0,9	0,8	1,0	0,1
16:57687854	1,0	0,8	0,9	1,1	0,2
8:73982064	1,0	0,8	0,8	1,0	0,1
22:30973069	0,2	0,8	0,9	1,1	0,0
16:24828143	0,3	0,8	0,9	1,0	0,0
X:153218358	0,1	1,0	1,2	1,1	0,0
2:97474358	0,1	0,8	1,2	1,1	0,0
X:151869805	0,4	1,0	1,1	1,1	0,0
2:228147100	0,3	0,6	0,6	1,2	0,1
19:54261563	1,1	0,1	0,4	0,0	0,0
X:77913206	1,0	0,2	0,6	0,0	0,0
13:52972327	1,0	0,1	0,4	0,0	0,1
5:110097136	1,0	0,1	0,3	0,0	0,0
14:103418903	0,9	0,1	0,4	0,0	0,0

IV. Phylogenetic tree based on original data

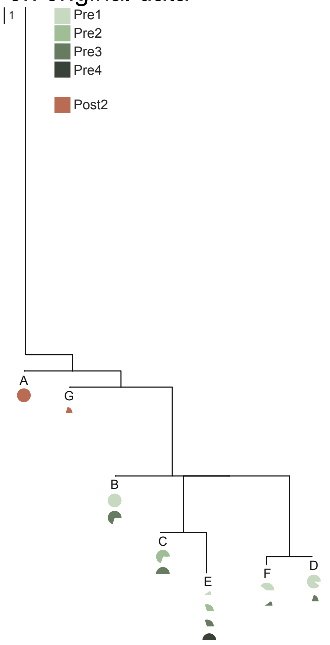

V.

SNVs originally detected	81
SNVs on CNAs without probability score in DeCiFer	39
SNVs not assigned to a cluster by DeCiFer	37

e. Patient 6 (4n)

I.

VAFs

	Pre1	Post1	Post2	Post3	Post4	
12:70949723	0,4	0,3	0,4	0,2	0,3	▲
21:38095404	0,2	0,0	0,0	0,0	0,0	▲
5:41201636	0,2	0,0	0,0	0,0	0,0	▲
8:144790019	0,2	0,0	0,0	0,0	0,0	▲
22:31011463	0,2	0,0	0,0	0,0	0,0	
17:36485406	0,2	0,0	0,0	0,0	0,0	▲
7:100662038	0,1	0,0	0,0	0,0	0,0	▲
10:24825621	0,0	0,0	0,3	0,0	0,0	▲
14:93151449	0,0	0,0	0,3	0,0	0,0	▲
7:43827557	0,0	0,0	0,2	0,0	0,0	▲
17:48265971	0,0	0,0	0,3	0,0	0,0	▲
9:139405727	0,0	0,0	0,3	0,0	0,0	▲
8:1616661	0,0	0,0	0,2	0,0	0,0	▲
5:140745456	0,0	0,0	0,2	0,0	0,0	▲
3:32932653	0,0	0,0	0,2	0,0	0,0	▲
18:34378538	0,0	0,0	0,2	0,0	0,0	▲
2:99012939	0,0	0,0	0,2	0,0	0,0	
20:58564309	0,0	0,0	0,0	0,0	0,2	
1:100731921	0,0	0,0	0,0	0,0	0,2	▲
16:72156855	0,0	0,0	0,0	0,0	0,2	▲
4:54011676	0,0	0,0	0,0	0,0	0,2	▲
6:43044748	0,0	0,0	0,0	0,0	0,2	▲
8:121706096	0,0	0,0	0,0	0,0	0,2	▲
10:52576069	0,0	0,0	0,0	0,0	0,2	▲
4:57777257	0,0	0,0	0,0	0,0	0,1	▲
5:141051106	0,0	0,0	0,0	0,0	0,1	▲
1:916362	0,0	0,0	0,0	0,0	0,1	▲
3:58064498	0,0	0,0	0,0	0,2	0,0	▲
1:6679978	0,0	0,0	0,0	0,2	0,0	▲
19:11325121	0,0	0,2	0,0	0,0	0,0	▲
22:17072765	0,0	0,1	0,0	0,0	0,0	
12:77222187	0,0	0,1	0,0	0,0	0,0	▲
11:7111477	0,0	0,2	0,0	0,0	0,0	▲
7:99306748	0,0	0,1	0,0	0,0	0,0	▲
19:57723439	0,0	0,1	0,0	0,0	0,0	▲
1:182569467	0,0	0,1	0,0	0,0	0,0	▲
4:110791192	0,0	0,1	0,0	0,0	0,0	▲
X:63488593	0,0	0,3	0,0	0,0	0,0	
8:96057774	0,0	0,2	0,0	0,0	0,0	▲
5:149584317	0,0	0,1	0,0	0,0	0,0	▲
17:62487031	0,0	0,2	0,0	0,0	0,0	▲

II.

Original clone sizes

	Pre1	Post1	Post2	Post3	Post4	subclone
12:70949723	1,1	0,9	1,1	1,2	1,0	stem
21:38095404	1,1	0,0	0,0	0,0	0,0	A
5:41201636	1,1	0,0	0,0	0,0	0,0	A
8:144790019	1,0	0,0	0,0	0,0	0,0	A
22:31011463	1,0	0,0	0,0	0,0	0,0	A
17:36485406	0,9	0,0	0,0	0,0	0,0	A
7:100662038	0,7	0,0	0,0	0,0	0,0	E
10:24825621	0,0	0,0	1,2	0,0	0,0	B
14:93151449	0,0	0,0	1,2	0,0	0,0	B
7:43827557	0,0	0,0	1,1	0,0	0,0	B
17:48265971	0,0	0,0	1,1	0,0	0,0	B
9:139405727	0,0	0,0	1,1	0,0	0,0	B
8:1616661	0,0	0,0	1,1	0,0	0,0	B
5:140745456	0,0	0,0	1,1	0,0	0,0	B
3:32932653	0,0	0,0	1,1	0,0	0,0	B
18:34378538	0,0	0,0	1,0	0,0	0,0	B
2:99012939	0,0	0,0	1,0	0,0	0,0	B
20:58564309	0,0	0,0	0,0	0,0	1,2	C
1:100731921	0,0	0,0	0,0	0,0	1,1	C
16:72156855	0,0	0,0	0,0	0,0	1,1	C
4:54011676	0,0	0,0	0,0	0,0	1,1	C
6:43044748	0,0	0,0	0,0	0,0	1,1	C
8:121706096	0,0	0,0	0,0	0,0	1,1	C
10:52576069	0,0	0,0	0,0	0,0	1,0	C
4:57777257	0,0	0,0	0,0	0,0	0,7	G
5:141051106	0,0	0,0	0,0	0,0	0,6	G
1:916362	0,0	0,0	0,0	0,0	0,6	G
3:58064498	0,0	0,0	0,0	0,7	0,0	F
1:6679978	0,0	0,0	0,0	0,6	0,0	F
19:11325121	0,0	1,1	0,0	0,0	0,0	D
22:17072765	0,0	1,0	0,0	0,0	0,0	D
12:77222187	0,0	1,0	0,0	0,0	0,0	D
11:7111477	0,0	1,0	0,0	0,0	0,0	D
7:99306748	0,0	0,9	0,0	0,0	0,0	D
19:57723439	0,0	0,9	0,0	0,0	0,0	D
1:182569467	0,0	0,9	0,0	0,0	0,0	D
4:110791192	0,0	0,9	0,0	0,0	0,0	D
X:63488593	0,0	0,9	0,0	0,0	0,0	D
8:96057774	0,0	0,9	0,0	0,0	0,0	D
5:149584317	0,0	0,9	0,0	0,0	0,0	D
17:62487031	0,0	0,8	0,0	0,0	0,0	D

III.

Clone sizes calculated with DeCiFer

	Pre1	Post1	Post2	Post3	Post4
22:31011463	1,2	0,0	0,0	0,0	0,0
2:99012939	0,0	0,0	1,3	0,0	0,0
20:58564309	0,0	0,0	0,0	0,0	0,8
22:17072765	0,0	0,8	0,0	0,0	0,0
X:63488593	0,0	1,2	0,0	0,0	0,0

IV.

Phylogenetic tree based on original data

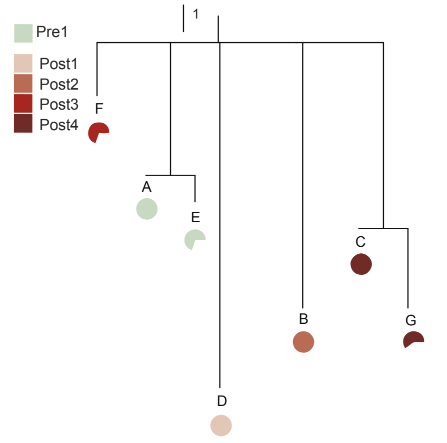

V.

SNVs originally detected	41
SNVs on CNAs without probability score in DeCiFer	36
SNVs not assigned to a cluster by DeCiFer	1

f. Patient 7 (4n)

I.

VAFs

	Pre1	Pre2	Post1	Post2	Post3
1:53736908	0,3	0,1	0,1	0,0	0,1
2:171655329	0,3	0,2	0,4	0,1	0,3
1:100542849	0,3	0,2	0,3	0,2	0,2
19:44514912	0,2	0,1	0,1	0,1	0,1
7:100685011	0,2	0,1	0,2	0,0	0,0
19:9058832	0,2	0,1	0,1	0,0	0,0
12:51442950	0,2	0,1	0,1	0,0	0,0
22:22550209	0,2	0,0	0,1	0,0	0,0
15:63845936	0,2	0,1	0,1	0,0	0,0
2:160862179	0,0	0,0	0,1	0,0	0,0
2:28754972	0,0	0,0	0,0	0,7	0,8

II.

Original clone sizes

	Pre1	Pre2	Post1	Post2	Post3	subclone
1:53736908	1,0	1,0	1,0	1,0	1,0	stem
2:171655329	0,9	1,0	1,3	0,9	1,1	stem
1:100542849	0,9	0,8	0,8	0,9	1,0	stem
19:44514912	0,9	0,8	1,2	1,3	1,1	stem
7:100685011	1,0	0,9	1,0	0,0	0,0	A
19:9058832	1,2	0,7	0,8	0,0	0,0	A
12:51442950	1,1	1,0	1,1	0,0	0,0	A
22:22550209	1,2	0,4	1,1	0,0	0,0	D
15:63845936	1,2	0,6	1,0	0,0	0,0	D
2:160862179	0,0	0,0	1,1	0,0	0,0	C
2:28754972	0,0	0,0	0,0	1,0	1,0	B

III.

Clone sizes calculated with DeCiFer

	Pre1	Pre2	Post1	Post2	Post3
19:44514912	1,0	0,9	1,1	1,2	1,0
19:9058832	1,3	0,9	0,7	0,0	0,0
12:51442950	1,2	1,1	1,0	0,0	0,0
15:63845936	1,3	0,7	0,9	0,0	0,0

IV.

Phylogenetic tree based on original data

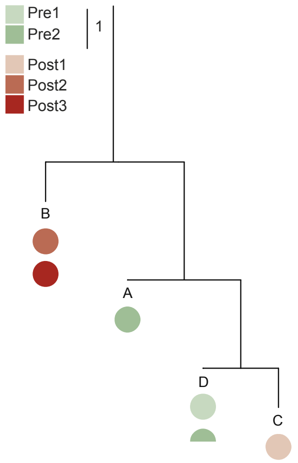

V.

SNVs originally detected	11
SNVs on CNAs without probability score in DeCiFer	7
SNVs not assigned to a cluster by DeCiFer	2

g. Patient 8 (4n)

I.

VAFs

	Pre1	Pre2	Pre3	PostA1	PostA2	PostA3	PostA4	PostB1	PostB2	PostB3	PostB4	
17:55184483	0,4	0,4	0,4	0,4	0,3	0,4	0,2	0,2	0,2	0,2	0,2	▲
19:9727817	0,4	0,2	0,3	0,3	0,3	0,5	0,2	0,1	0,1	0,2	0,1	▲
1:179504153	0,2	0,3	0,1	0,2	0,2	0,3	0,1	0,2	0,1	0,2	0,1	▲
17:57287727	0,2	0,2	0,2	0,2	0,1	0,3	0,1	0,1	0,1	0,1	0,1	▲
17:53150381	0,0	0,0	0,0	0,1	0,1	0,2	0,2	0,0	0,0	0,0	0,0	▲
1:1271681	0,0	0,0	0,0	0,1	0,1	0,2	0,1	0,0	0,0	0,0	0,0	▲
16:66757725	0,0	0,0	0,0	0,0	0,0	0,3	0,1	0,0	0,0	0,0	0,0	
2:202163967	0,0	0,0	0,0	0,0	0,0	0,2	0,0	0,0	0,0	0,0	0,0	▲
20:40713320	0,0	0,0	0,0	0,0	0,0	0,1	0,0	0,0	0,0	0,0	0,0	

II.

Original clone sizes

	Pre1	Pre2	Pre3	PostA1	PostA2	PostA3	PostA4	PostB1	PostB2	PostB3	PostB4	subclone
17:55184483	0,9	1,0	0,9	1,2	1,1	1,0	0,8	1,2	1,0	0,8	1,0	stem
1:179504153	0,7	1,0	0,4	1,1	1,3	1,1	1,1	1,5	0,9	1,6	0,8	stem
17:57287727	0,9	1,2	1,1	1,2	0,7	1,3	1,0	1,0	1,0	0,9	0,9	stem
19:9727817	1,0	0,5	0,7	1,2	0,9	0,9	0,9	0,8	0,9	1,1	0,8	stem
1:1271681	0,0	0,0	0,0	0,5	1,0	1,1	0,9	0,0	0,0	0,0	0,0	C
17:53150381	0,0	0,0	0,0	0,4	1,0	1,0	1,4	0,0	0,0	0,0	0,0	C
16:66757725	0,0	0,0	0,0	0,0	0,0	0,9	0,7	0,0	0,0	0,0	0,0	B
2:202163967	0,0	0,0	0,0	0,0	0,0	1,2	0,0	0,0	0,0	0,0	0,0	A
20:40713320	0,0	0,0	0,0	0,0	0,0	0,8	0,0	0,0	0,0	0,0	0,0	A

III.

Clone sizes calculated with DeCiFer

	Pre1	Pre2	Pre3	PostA1	PostA2	PostA3	PostA4	PostB1	PostB2	PostB3	PostB4
16:66757725	0,0	0,0	0,0	0,1	0,5	1,2	1,2	0,0	0,1	0,0	0,0
20:40713320	0,0	0,0	0,0	0,0	0,1	1,1	0,6	0,0	0,1	0,0	0,0

IV.

Phylogenetic tree based on original data

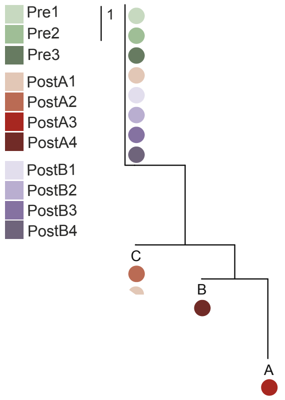

V.

SNVs originally detected	9
SNVs on CNAs without probability score in DeCiFer	7
SNVs not assigned to a cluster by DeCiFer	0

h. Patient 10 (4n)

Suppl Fig 8

I.

VAFs

	Pre1	Pre2	Pre3	Pre4	MR1	MR2	MR3	MR4	
6:25769239	0,2	0,2	0,1	0,2	0,3	0,3	0,4	0,2	▲
16:53913822	0,2	0,2	0,1	0,2	0,3	0,4	0,3	0,2	▲
12:98941301	0,1	0,1	0,1	0,1	0,2	0,2	0,3	0,1	▲
15:102241291	0,2	0,2	0,1	0,2	0,3	0,3	0,4	0,2	
15:102261465	0,2	0,2	0,1	0,3	0,3	0,3	0,4	0,1	
12:120616495	0,2	0,2	0,2	0,3	0,2	0,2	0,2	0,1	▲
2:141660540	0,1	0,1	0,0	0,2	0,1	0,2	0,2	0,1	▲
1:152285079	0,2	0,2	0,2	0,3	0,3	0,3	0,4	0,2	
4:155489497	0,1	0,1	0,0	0,1	0,3	0,3	0,4	0,2	
19:11943705	0,2	0,2	0,1	0,3	0,3	0,3	0,4	0,2	
X:117900851	0,0	0,0	0,0	0,0	0,2	0,2	0,3	0,1	▲
22:46403078	0,0	0,0	0,0	0,0	0,1	0,2	0,2	0,1	
1:27059196	0,0	0,0	0,0	0,0	0,2	0,2	0,2	0,1	
19:856154	0,0	0,0	0,0	0,0	0,1	0,2	0,2	0,1	
17:33353517	0,0	0,0	0,0	0,0	0,2	0,2	0,3	0,1	▲
2:198949533	0,0	0,0	0,0	0,0	0,2	0,2	0,2	0,1	▲
12:56088592	0,0	0,0	0,0	0,0	0,2	0,2	0,2	0,1	▲
21:38137508	0,0	0,0	0,0	0,0	0,2	0,2	0,2	0,1	
14:22521102	0,0	0,0	0,0	0,0	0,1	0,1	0,2	0,1	▲
12:40834578	0,0	0,0	0,0	0,0	0,1	0,2	0,2	0,1	▲
10:98799731	0,0	0,0	0,0	0,0	0,3	0,3	0,4	0,2	
14:24779861	0,0	0,0	0,0	0,0	0,1	0,1	0,2	0,1	▲
15:85632568	0,0	0,0	0,0	0,0	0,2	0,2	0,2	0,1	
4:88116540	0,0	0,0	0,0	0,0	0,3	0,3	0,4	0,2	
14:47342756	0,0	0,0	0,0	0,0	0,1	0,1	0,1	0,1	▲
2:26508378	0,0	0,0	0,0	0,0	0,2	0,1	0,2	0,1	▲
8:27922112	0,0	0,0	0,0	0,0	0,1	0,2	0,2	0,1	▲
4:69098054	0,0	0,0	0,0	0,0	0,1	0,2	0,2	0,1	
3:123675200	0,0	0,0	0,0	0,0	0,2	0,2	0,2	0,1	▲
12:50186092	0,0	0,0	0,0	0,0	0,1	0,1	0,2	0,1	▲
11:102819808	0,0	0,0	0,0	0,0	0,2	0,3	0,3	0,1	▲
15:60641310	0,0	0,0	0,0	0,0	0,2	0,1	0,2	0,1	
1:113241385	0,0	0,0	0,0	0,0	0,1	0,1	0,2	0,1	
1:149858851	0,0	0,0	0,0	0,0	0,1	0,2	0,2	0,1	
19:47823200	0,0	0,0	0,0	0,0	0,1	0,2	0,1	0,1	
3:38043300	0,0	0,0	0,0	0,0	0,2	0,2	0,3	0,1	▲
1:19526187	0,0	0,0	0,0	0,0	0,1	0,2	0,2	0,1	
19:9077798	0,0	0,0	0,0	0,0	0,1	0,2	0,2	0,1	
15:99440045	0,0	0,0	0,0	0,0	0,2	0,1	0,2	0,1	
12:50186107	0,0	0,0	0,0	0,0	0,1	0,2	0,2	0,1	▲
10:70652529	0,0	0,0	0,0	0,0	0,1	0,2	0,2	0,1	
4:159789451	0,0	0,0	0,0	0,0	0,3	0,3	0,4	0,1	
4:187630428	0,0	0,0	0,0	0,0	0,3	0,4	0,4	0,1	
11:66314209	0,0	0,0	0,0	0,0	0,1	0,1	0,1	0,1	▲
11:118267168	0,0	0,0	0,0	0,0	0,2	0,2	0,2	0,1	▲
3:48604078	0,0	0,0	0,0	0,0	0,2	0,2	0,3	0,1	▲
17:33749492	0,0	0,0	0,0	0,0	0,1	0,2	0,2	0,1	▲
7:43485122	0,0	0,0	0,0	0,0	0,1	0,1	0,1	0,0	▲
8:3047594	0,0	0,0	0,0	0,0	0,2	0,2	0,2	0,0	▲
4:4421857	0,0	0,0	0,0	0,0	0,2	0,2	0,2	0,0	
10:53059353	0,0	0,0	0,0	0,0	0,1	0,1	0,2	0,0	

II.

Original clone sizes

	Pre1	Pre3	Pre2	Pre4	MR1	MR2	MR3	MR4	
6:25769239	1,1	0,8	0,9	1,0	1,0	1,0	1,1	0,9	stem
16:53913822	1,0	0,7	0,7	0,9	1,0	1,0	0,8	1,0	stem
12:98941301	0,7	0,9	0,7	1,0	0,9	1,2	0,7	1,0	stem
15:102241291	0,9	1,2	0,7	0,8	0,8	1,0	1,0	0,9	stem
15:102261465	1,0	0,7	0,8	1,0	0,9	0,9	1,0	0,8	stem
12:120616495	1,2	1,0	1,1	1,2	0,9	0,9	1,0	1,1	stem
2:141660540	0,9	0,6	0,8	1,1	0,9	0,9	0,9	0,7	stem
1:152285079	0,9	1,1	0,9	1,2	1,0	0,9	1,1	1,1	stem
4:155489497	0,8	0,5	0,9	0,9	1,0	1,0	1,0	1,1	stem
19:11943705	0,8	0,6	1,0	1,0	0,9	0,9	1,1	0,9	stem
X:117900851	0,0	0,0	0,0	0,0	1,0	1,2	1,2	1,0	A
22:46403078	0,0	0,0	0,0	0,0	0,8	1,3	1,2	0,9	A
1:27059196	0,0	0,0	0,0	0,0	0,9	1,1	1,2	1,0	A
19:856154	0,0	0,0	0,0	0,0	0,9	1,0	0,9	1,2	A
17:33353517	0,0	0,0	0,0	0,0	1,0	1,0	0,9	0,9	A
2:198949533	0,0	0,0	0,0	0,0	1,1	1,2	1,0	1,0	A
12:56088592	0,0	0,0	0,0	0,0	1,0	1,0	1,0	0,9	A
21:38137508	0,0	0,0	0,0	0,0	1,0	1,2	1,0	0,9	A
14:22521102	0,0	0,0	0,0	0,0	0,9	1,0	0,9	0,9	A
12:40834578	0,0	0,0	0,0	0,0	0,9	0,9	0,9	0,9	A
10:98799731	0,0	0,0	0,0	0,0	1,0	1,0	1,0	1,1	A
14:24779861	0,0	0,0	0,0	0,0	1,0	0,9	1,0	1,0	A
15:85632568	0,0	0,0	0,0	0,0	1,0	1,1	0,9	1,0	A
4:88116540	0,0	0,0	0,0	0,0	0,9	0,9	1,0	1,0	A
14:47342756	0,0	0,0	0,0	0,0	1,0	0,9	0,8	1,1	A
2:26508378	0,0	0,0	0,0	0,0	1,1	0,8	0,9	1,3	A
8:27922112	0,0	0,0	0,0	0,0	0,8	1,1	0,9	1,1	A
4:69098054	0,0	0,0	0,0	0,0	0,7	1,3	1,0	1,0	A
3:123675200	0,0	0,0	0,0	0,0	0,8	0,8	1,0	1,0	A
12:50186092	0,0	0,0	0,0	0,0	0,9	0,8	0,9	1,0	A
11:102819808	0,0	0,0	0,0	0,0	0,8	1,1	1,0	1,0	A
15:60641310	0,0	0,0	0,0	0,0	0,9	0,8	0,9	1,0	A
1:113241385	0,0	0,0	0,0	0,0	0,7	0,8	0,8	1,0	A
1:149858851	0,0	0,0	0,0	0,0	0,6	1,2	1,1	0,9	A
19:47823200	0,0	0,0	0,0	0,0	0,9	0,9	0,8	0,9	A
3:38043300	0,0	0,0	0,0	0,0	0,8	0,9	1,2	0,9	A
1:19526187	0,0	0,0	0,0	0,0	0,8	1,0	0,8	0,9	A
19:9077798	0,0	0,0	0,0	0,0	0,8	1,0	1,2	0,8	A
15:99440045	0,0	0,0	0,0	0,0	1,0	0,8	1,1	0,8	A
12:50186107	0,0	0,0	0,0	0,0	0,8	1,1	0,9	0,8	A
10:70652529	0,0	0,0	0,0	0,0	0,8	0,9	0,9	0,8	A
4:159789451	0,0	0,0	0,0	0,0	0,8	0,9	1,1	0,8	A
4:187630428	0,0	0,0	0,0	0,0	1,0	1,0	1,1	0,8	A
11:66314209	0,0	0,0	0,0	0,0	1,0	1,0	0,9	0,7	A
11:118267168	0,0	0,0	0,0	0,0	1,0	0,9	0,9	0,7	A
3:48604078	0,0	0,0	0,0	0,0	0,9	1,1	1,2	0,7	A
17:33749492	0,0	0,0	0,0	0,0	0,8	0,9	0,9	0,7	A
7:43485122	0,0	0,0	0,0	0,0	1,1	1,0	1,0	0,6	A
8:3047594	0,0	0,0	0,0	0,0	1,3	1,0	1,2	0,6	A
4:4421857	0,0	0,0	0,0	0,0	1,1	1,0	1,0	0,5	A
10:53059353	0,0	0,0	0,0	0,0	0,9	0,9	1,2	0,5	A

III.

Clone sizes calculated with DeCiFer

	Pre1	Pre2	Pre3	Pre4	MR1	MR2	MR3	MR4
15:102241291	1,0	0,9	1,5	0,8	0,9	1,0	0,9	0,9
15:102261465	1,0	1,0	0,8	1,0	0,9	1,0	0,9	0,9
1:152285079	1,0	1,0	1,0	1,2	1,1	1,0	1,1	1,1
4:155489497	0,9	1,0	0,7	1,0	1,0	1,0	1,0	1,1
19:11943705	0,9	0,8	0,8	1,1	1,0	1,0	1,1	0,9
22:46403078	0,0	0,0	0,0	0,0	0,9	1,4	1,2	0,9
1:27059196	0,0	0,0	0,0	0,0	1,0	1,1	1,2	1,1
19:856154	0,0	0,0	0,0	0,0	1,0	1,0	0,8	1,2
21:38137508	0,0	0,0	0,0	0,0	1,0	1,2	1,0	0,9
10:98799731	0,0	0,0	0,0	0,0	1,1	1,0	1,0	1,1
15:85632568	0,0	0,0	0,0	0,0	1,1	1,1	0,9	1,0
4:88116540	0,0	0,0	0,0	0,0	1,0	1,0	1,0	1,0
4:69098054	0,0	0,0	0,0	0,0	0,8	1,3	0,9	1,0
15:60641310	0,0	0,0	0,0	0,0	1,0	0,9	0,9	1,0
1:113241385	0,0	0,0	0,0	0,0	0,8	0,8	0,8	1,0
1:149858851	0,0	0,0	0,0	0,0	0,7	1,2	1,1	0,9
19:47823200	0,0	0,0	0,0	0,0	1,0	0,9	0,8	0,9
1:19526187	0,0	0,0	0,0	0,0	0,8	1,1	0,8	0,9
19:9077798	0,0	0,0	0,0	0,0	0,8	1,0	1,1	0,9
15:99440045	0,0	0,0	0,0	0,0	1,1	0,8	1,1	0,9
10:70652529	0,0	0,0	0,0	0,0	0,9	0,9	0,9	0,8
4:159789451	0,0	0,0	0,0	0,0	0,9	0,9	1,1	0,8
4:187630428	0,0	0,0	0,0	0,0	1,1	1,1	1,0	0,8
4:4421857	0,0	0,0	0,0	0,0	1,2	1,1	1,0	0,6
10:53059353	0,0	0,0	0,0	0,0	0,9	0,9	1,1	0,5

IV.

Phylogenetic tree based on original data

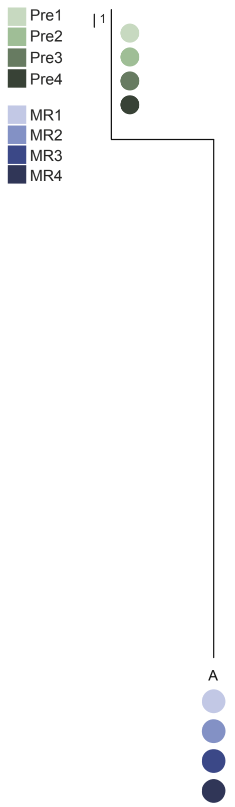

V.

SNVs originally detected	51
SNVs on CNAs without probability score in DeCiFer	26
SNVs not assigned to a cluster by DeCiFer	15

i. Patient 11 (4n)

Suppl Fig 8

I.

VAFs

	Post1	Post3	Post4	MR1	MR2	
2:71351549	0,2	0,1	0,1	0,4	0,4	▲
1:112045814	0,1	0,2	0,1	0,2	0,3	
17:7578403	0,0	0,0	0,0	0,4	0,5	▲
2:158485128	0,0	0,0	0,0	0,2	0,1	▲
1:27690040	0,0	0,0	0,0	0,1	0,1	
19:1009522	0,0	0,0	0,0	0,1	0,1	

II.

Original clone sizes

	Post1	Post3	Post4	MR1	MR2	subclone
2:71351549	1,0	1,0	1,0	1,0	1,1	Stem
1:112045814	1,0	1,0	1,0	0,8	1,2	Stem
17:7578403	0,0	0,0	0,0	1,1	1,3	A
2:158485128	0,0	0,0	0,0	1,4	0,9	A
1:27690040	0,0	0,0	0,0	0,7	0,8	B
19:1009522	0,0	0,0	0,0	0,6	0,7	B

III.

Clone sizes calculated with DeCiFer

	Post1	Post3	Post4	MR1	MR2
1:112045814	1,0	1,5	1,3	1,2	0,9
1:27690040	0,0	0,0	0,0	1,0	1,2
19:1009522	0,0	0,0	0,0	0,9	1,0

IV.

Phylogenetic tree based on original data

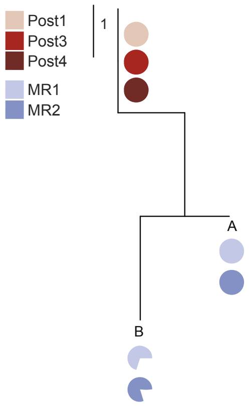

V.

SNVs originally detected	6
SNVs on CNAs without probability score in DeCiFer	3
SNVs not assigned to a cluster by DeCiFer	0

j. Patient 12 (2n)

I.

VAFs

	Pre1	Pre2	Pre3	MR1
10:43623541	0,4	0,5	0,4	0,3
12:10782086	0,4	0,4	0,5	0,3
1:232943750	0,4	0,4	0,4	0,3
17:6381992	0,8	0,8	0,8	0,3
16:82104444	0,4	0,5	0,4	0,2
1:11086116	0,4	0,4	0,4	0,2
10:102286080	0,4	0,4	0,4	0,2
6:70669833	0,4	0,4	0,4	0,2
20:49576731	0,4	0,5	0,4	0,2
5:58489362	0,5	0,4	0,4	0,2
3:63830747	0,5	0,4	0,4	0,2
2:233998573	0,4	0,4	0,5	0,2
12:53565844	0,4	0,5	0,5	0,2
14:102431252	0,5	0,5	0,4	0,3
4:77926641	0,8	0,6	0,9	0,4
1:100676193	0,4	0,4	0,4	0,2
16:48395847	0,4	0,5	0,4	0,2
4:110765234	0,4	0,3	0,4	0,2
13:23912863	0,3	0,3	0,3	0,3
3:39129652	0,3	0,4	0,5	0,3
3:123851653	0,3	0,1	0,5	0,0
18:14511971	0,3	0,1	0,4	0,0
15:92937412	0,3	0,1	0,4	0,0
14:23518335	0,0	0,4	0,0	0,0
10:135381757	0,0	0,3	0,0	0,0
2:228012226	0,0	0,3	0,0	0,0
13:32768256	0,0	0,2	0,0	0,0
1:200881330	0,0	0,3	0,0	0,0
3:119135064	0,0	0,3	0,0	0,0
11:49179588	0,0	0,2	0,0	0,0
4:108999551	0,0	0,2	0,0	0,0
14:63416887	0,0	0,2	0,0	0,0
2:215279046	0,0	0,0	0,0	0,3
10:124186506	0,0	0,0	0,0	0,3
3:152880425	0,0	0,0	0,0	0,3
10:134898430	0,0	0,0	0,0	0,3
3:47734764	0,0	0,0	0,0	0,3
20:33578623	0,0	0,0	0,0	0,3
2:91944010	0,0	0,0	0,0	0,3
5:169135219	0,0	0,0	0,0	0,2
4:143344979	0,0	0,0	0,0	0,2
5:158141004	0,0	0,0	0,0	0,2
17:46700134	0,0	0,0	0,0	0,1
8:124658341	0,0	0,0	0,0	0,2
15:75660309	0,0	0,0	0,0	0,2
19:51919289	0,0	0,0	0,0	0,2
17:56492647	0,0	0,0	0,0	0,1
17:72920987	0,0	0,0	0,0	0,1
1:248569381	0,0	0,0	0,0	0,2
21:45784039	0,0	0,0	0,0	0,4
12:54718920	0,0	0,0	0,0	0,2
7:158456984	0,0	0,0	0,0	0,1
19:43244650	0,0	0,0	0,0	0,2
6:80721212	0,0	0,0	0,0	0,2
8:86171580	0,0	0,0	0,0	0,2
1:17419059	0,0	0,0	0,0	0,2
6:141005051	0,0	0,0	0,0	0,2
17:38140783	0,0	0,0	0,0	0,1
5:153783677	0,0	0,0	0,0	0,2
11:68852726	0,0	0,0	0,0	0,2
8:30700555	0,0	0,0	0,0	0,2
13:32836333	0,0	0,0	0,0	0,3
11:113258630	0,0	0,0	0,0	0,2

II.

Original clone sizes

	Pre1	Pre2	Pre3	MR1	subclone
10:43623541	1,0	1,2	1,1	1,1	stem
12:10782086	1,0	1,1	1,1	1,1	stem
1:232943750	1,0	1,0	1,1	1,0	stem
17:6381992	1,1	1,3	1,2	1,0	stem
16:82104444	1,0	1,2	1,1	1,0	stem
1:11086116	1,0	1,1	1,0	1,0	stem
10:102286080	1,0	1,1	1,1	1,0	stem
6:70669833	1,0	1,1	1,1	1,0	stem
20:49576731	1,0	1,2	1,1	1,0	stem
5:58489362	1,1	1,1	1,1	1,0	stem
3:63830747	1,1	1,1	1,1	1,0	stem
2:233998573	1,0	1,1	1,1	1,0	stem
12:53565844	1,0	1,2	1,1	1,0	stem
14:102431252	1,1	1,0	1,0	1,0	stem
4:77926641	1,0	0,9	1,1	0,9	stem
1:100676193	1,0	1,1	1,1	0,9	stem
16:48395847	1,0	1,2	1,1	0,9	stem
4:110765234	1,0	1,0	1,1	1,0	stem
13:23912863	1,0	1,0	1,1	0,9	stem
3:39129652	0,7	1,1	1,2	1,1	A
3:123851653	0,7	0,2	1,1	0,0	C
18:14511971	0,6	0,2	1,1	0,0	C
15:92937412	0,7	0,3	1,0	0,0	C
14:23518335	0,0	0,8	0,0	0,0	D
10:135381757	0,0	0,7	0,0	0,0	D
2:228012226	0,0	0,7	0,0	0,0	D
13:32768256	0,0	0,7	0,0	0,0	D
1:200881330	0,0	0,7	0,0	0,0	D
3:119135064	0,0	0,7	0,0	0,0	D
11:49179588	0,0	0,6	0,0	0,0	D
4:108999551	0,0	0,5	0,0	0,0	F
14:63416887	0,0	0,5	0,0	0,0	F
2:215279046	0,0	0,0	0,0	1,2	B
10:124186506	0,0	0,0	0,0	1,1	B
3:152880425	0,0	0,0	0,0	1,1	B
10:134898430	0,0	0,0	0,0	1,1	B
3:47734764	0,0	0,0	0,0	1,1	B
20:33578623	0,0	0,0	0,0	1,0	B
2:91944010	0,0	0,0	0,0	1,0	B
5:169135219	0,0	0,0	0,0	1,0	B
4:143344979	0,0	0,0	0,0	1,0	B
5:158141004	0,0	0,0	0,0	1,0	B
17:46700134	0,0	0,0	0,0	1,0	B
8:124658341	0,0	0,0	0,0	1,0	B
15:75660309	0,0	0,0	0,0	1,0	B
19:51919289	0,0	0,0	0,0	1,0	B
17:56492647	0,0	0,0	0,0	1,0	B
17:72920987	0,0	0,0	0,0	1,0	B
1:248569381	0,0	0,0	0,0	1,0	B
21:45784039	0,0	0,0	0,0	1,0	B
12:54718920	0,0	0,0	0,0	1,0	B
7:158456984	0,0	0,0	0,0	1,0	B
19:43244650	0,0	0,0	0,0	1,0	B
6:80721212	0,0	0,0	0,0	1,0	B
8:86171580	0,0	0,0	0,0	1,0	B
1:17419059	0,0	0,0	0,0	0,9	B
6:141005051	0,0	0,0	0,0	0,9	B
17:38140783	0,0	0,0	0,0	0,9	B
5:153783677	0,0	0,0	0,0	0,9	B
11:68852726	0,0	0,0	0,0	0,9	B
8:30700555	0,0	0,0	0,0	0,9	B
13:32836333	0,0	0,0	0,0	0,9	B
11:113258630	0,0	0,0	0,0	0,7	E

III.

Clone sizes calculated with DeCiFer

	Pre1	Pre2	Pre3	MR1
10:43623541	1,0	1,1	1,0	1,1
12:10782086	1,0	1,0	1,0	1,0
1:232943750	1,0	0,9	1,0	1,0
17:6381992	0,9	0,9	0,9	0,7
16:82104444	1,0	1,0	1,0	1,0
1:11086116	1,0	1,0	1,0	1,0
10:102286080	1,0	1,0	1,0	1,0
6:70669833	1,0	1,0	1,0	1,0
20:49576731	1,0	1,0	1,0	1,0
5:58489362	1,0	1,0	1,0	1,0
3:63830747	1,0	1,0	1,0	1,0
2:233998573	1,0	1,0	1,0	0,9
12:53565844	1,0	1,0	1,0	0,9
14:102431252	1,0	1,0	0,9	1,3
4:77926641	1,0	0,9	1,0	1,1
1:100676193	1,0	1,0	1,0	0,9
16:48395847	1,0	1,1	1,0	0,9
4:110765234	1,0	0,9	1,0	1,2
13:23912863	1,0	1,0	1,1	1,0
3:39129652	0,7	1,0	1,1	1,1
3:123851653	0,7	0,2	1,0	0,0
18:14511971	0,6	0,2	1,0	0,0
15:92937412	0,7	0,3	0,9	0,0
14:23518335	0,1	0,8	0,0	0,0
10:135381757	0,1	0,6	0,0	0,0
2:228012226	0,1	0,6	0,0	0,0
13:32768256	0,1	0,7	0,0	0,0
1:200881330	0,1	0,6	0,0	0,0
3:119135064	0,0	0,6	0,0	0,0
11:49179588	0,0	0,5	0,0	0,0
4:108999551	0,0	0,5	0,0	0,0
14:63416887	0,0	0,5	0,0	0,0
2:215279046	0,0	0,0	0,0	1,1
10:124186506	0,0	0,0	0,0	1,1
3:152880425	0,0	0,0	0,0	1,1
10:134898430	0,0	0,0	0,0	1,1
3:47734764	0,0	0,0	0,0	1,1
20:33578623	0,0	0,0	0,0	1,0
2:91944010	0,0	0,0	0,0	1,0
5:169135219	0,0	0,0	0,0	1,0
4:143344979	0,0	0,0	0,0	1,2
5:153783677	0,0	0,0	0,0	0,9
5:158141004	0,0	0,0	0,0	1,0
17:46700134	0,0	0,0	0,0	1,5
8:124658341	0,0	0,0	0,0	1,2
15:75660309	0,0	0,0	0,0	1,0
19:51919289	0,0	0,0	0,0	1,0
17:56492647	0,0	0,0	0,0	1,4
17:72920987	0,0	0,0	0,0	1,4
1:248569381	0,0	0,0	0,0	1,0
21:45784039	0,0	0,0	0,0	1,0
12:54718920	0,0	0,0	0,0	1,0
7:158456984	0,0	0,0	0,0	1,4
19:43244650	0,0	0,0	0,0	1,0
6:80721212	0,0	0,0	0,0	0,9
8:86171580	0,0	0,0	0,0	1,1
1:17419059	0,0	0,0	0,0	0,9
6:141005051	0,0	0,0	0,0	0,9
17:38140783	0,0	0,0	0,0	1,3
11:68852726	0,0	0,0	0,0	0,9
8:30700555	0,0	0,0	0,0	1,0
13:32836333	0,0	0,0	0,0	1,0
11:113258630	0,0	0,0	0,0	0,7

IV. Phylogenetic tree based on original data

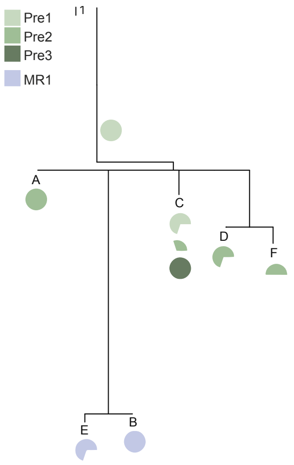

V.

SNVs originally detected	63
SNVs on CNAs without probability score in DeCiFer	0
SNVs not assigned to a cluster by DeCiFer	30

Supplementary Figure 8. Comparisons between original MCFs and MCFs obtained by DeCiFer in clinical samples. a-j. VAFs for all identified mutations are displayed as heatmaps where each column represents a sample and each row a variant denoted by its genomic position (**I**). Variants present on a chromosomal copy number aberration are marked by black borders. Details regarding the copy number background for the specific variants in the different samples are found in **Supplementary Data 2b**. The variants in heatmaps **I**, **II** and **III** are displayed in the same order, which is according to the clustering of the variants done visually based on similarities in MCF-patterns over the different samples. The final clusters are illustrated by gaps in heatmap **II**. The final MCFs were obtained by calculating the median of the MCFs in the different groups for the specific samples and the median was rounded to the nearest 0.1 and given to all variants in that group, see **Supplementary Data 1d** for final values. Clone sizes produced by DeCiFer are illustrated in **III**. The color scale is specific for each heat map, and the highest value in each plot is depicted with dark red and the lowest value with a light red. The remaining data points are illustrated at a color scale ranging between these values. All individual values are depicted in the plot."Variants not assigned to a cluster by DeCiFer are marked in orange and variants lacking an MCF-value due to their location on a copy number segment with an allelic composition for which DeCiFer lacked a default state tree estimate are indicated by a blue triangle to the right of heatmap **I**. For each case, a cleaned version of the phylogenetic tree in **Supplementary Fig 1 (a-i)** is also shown in **IV** to facilitate easy comparison between the appearance of the heatmaps (**I-III**). The letters to the right of the heatmaps with original MCFs (**II**) correspond to the cluster names given by DEVOLUTION, shown at the branches of the phylogenetic trees (**IV**). The number of originally identified SNVs and SNVs excluded by DeCiFer are shown in **V**. The global appearance of the original MCF values (heatmap II) and the DeCiFer generated MCFs (heatmap III) were highly similar for most of the tumors. Differences that would result in minor differences in the clonal landscapes were detected in two patients, Patient1 (**Supplementary Fig. 8a**) and Patient5 (**Supplementary Fig. 8d**). Patient 1 **(a)** had a diploid tumor, where differences were detected in the Pre-samples for three variants marked by arrows. The two variants on chr 6 (gray and red arrows respectively) were located on a 1+2/2+0 allelic background in all Pre-samples. In the original MCF calculation we assumed the number of mutated alleles (M) to be two in both those fractions (**Supplementary Data 2b**), since that assumption generated MCF values <1 for all samples and MCF values that correlated with the overall appearance of the subclonal landscape (Heatmap **II**). The SNV on chr 4 (purple arrows) was located on a 1+1 background in samples Pre1 and Pre2, while a subclonal gain to 1+3 took place in Pre3 and Pre4 (**Supplementary Data 2b** and **heatmap d:I)**. In the original calculation, it was assumed that the subclonal gain took place on the mutated allele. This assumption was based on the same rationale as for the variants on chr 6, i.e. it generated a logical clonal landscape. The DeCiFer generated MCFs for the same variants (heatmap **III**) became higher and would not have made it possible to create a consistent phylogenetic tree, because it is impossible to fit a subclone containing the variant on chr 4 (red arrow) above or below the variant on chr 6 (grey arrow) in a tree. For patient 5 **(d),** a gray line illustrates differences between original MCFs and MCFs obtained by DeCiFer in several clusters in the tetraploid tumor. M had to be 2 in sample Pre1 in clusters A, G and B (heatmap II), since M=1 would generate MCF values close to 200%, and M>2 is unlikely on a 2+2 segment. Since the copy number background was identical in these cluster for all four pre samples (see heatmap **d:I,** and **Supplementary Data 2b**), the number of mutated alleles was assumed to be two for the other three pre-samples too, resulting in a 50% subclone in Pre4 since this sample had lower VAFs for these mutations (heatmap **d:I** and **Supplementary Data 2b**). DeCiFer on the other hand, assigned clonal values for these variants in sample Pre4

(heatmap d:III), hence assuming M=1 and implying a reverse order of events in Pre4 compared to the other Pre samples, i.e. the unlikely scenario that the SNVs present in all Pre-samples appeared after the genome duplication in one part of the tumor only. In addition, the DeCiFer-generated clone sizes were surprisingly high for variants in Post2 cluster B, although their VAFs were low (median 0.01) and were removed from the study basedon low MCFs (<0.1). Such surprisingly high MCF values were also generated by DeCiFer for Patient 8 **(g)**. Here the SNV on chr 16 in PostA2 and on chr 20 in PostA4 both had VAFs < 0.03 and were thus excluded in the original MCF calculations.

References to Supplementary Material

1. Hoebeeck, J. *et al.* Aberrant methylation of candidate tumor suppressor genes in neuroblastoma. *Cancer Lett* **273**, 336-46 (2009).
2. Chang, H.H. *et al.* Notch1 expression predicts an unfavorable prognosis and serves as a therapeutic target of patients with neuroblastoma. *Clin Cancer Res* **16**, 4411-20 (2010).
3. Saito-Ohara, F. *et al.* PPM1D is a potential target for 17q gain in neuroblastoma. *Cancer Res* **63**, 1876-83 (2003).
4. Cuende, J., Moreno, S., Bolanos, J.P. & Almeida, A. Retinoic acid downregulates Rae1 leading to APC(Cdh1) activation and neuroblastoma SH-SY5Y differentiation. *Oncogene* **27**, 3339-44 (2008).
5. Dasgupta, A., Alvarado, C.S., Xu, Z. & Findley, H.W. Expression and functional role of inhibitor-of-apoptosis protein livin (BIRC7) in neuroblastoma. *Biochem Biophys Res Commun* **400**, 53-9 (2010).
6. Decock, A. *et al.* Genome-wide promoter methylation analysis in neuroblastoma identifies prognostic methylation biomarkers. *Genome Biol* **13**, R95 (2012).
7. Andersson, N. *et al.* Extensive Clonal Branching Shapes the Evolutionary History of High-Risk Pediatric Cancers. *Cancer Res* **80**, 1512-1523 (2020).
