# Supplementary material for: Early evolutionary branching across spatial domains predisposes to clonal replacement under chemotherapy in neuroblastoma
Source: Nat Commun. 2024 Oct 18;15:8992. doi: 10.1038/s41467-024-53334-x (PMC11486966; doi:10.1038/s41467-024-53334-x)
Supplement: Supplementary file 3 — Description of Additional Supplementary Files [file 41467_2024_53334_MOESM3_ESM.pdf]

Description of Additional Supplementary Files

Supplementary Data 1. Patient cohorts and subclone landscapes

a. Patient cohort for multiregional sampling

b. Patient cohort and PDXs for single cell whole genome sequencing

c. Patient cohort for FISH analysis

d. Clone size heat maps

Supplementary Data 2. Genome profiles of patient samples and model systems

a. Copy number profiles of patient samples

b. Targeted resequencing of clinical samples

c. Copy number profile of cisplatin treated PDX tumors

d. Mutations identified in cisplatin treated PDX tumors by whole exome sequencing

e. Clonal deconvolution of sequence mutations identified in cisplatin treated PDX tumors

f. Copy number profile of COJEC treated PDX tumors

g. Recurrent mutations in COJEC treated PDX tumors

h. Copy number profile evolution under in vitro chemotherapy, IMR-32 cells

i. Copy number profile evolution under in vitro chemotherapy, SK-N-SH cells
