# Supplementary material for: Early evolutionary branching across spatial domains predisposes to clonal replacement under chemotherapy in neuroblastoma
Source: Nat Commun. 2024 Oct 18;15:8992. doi: 10.1038/s41467-024-53334-x (PMC11486966; doi:10.1038/s41467-024-53334-x)
Supplement: Supplementary file 6 — Reporting Summary [file 41467_2024_53334_MOESM6_ESM.pdf]

nature portfolio

Corresponding author(s): Gisselsson, David

Last updated by author(s): 9/30/2024

Reporting Summary

Nature Portfolio wishes to improve the reproducibility of the work that we publish. This form provides structure for consistency and transparency in reporting. For further information on Nature Portfolio policies, see our Editorial Policies and the Editorial Policy Checklist.

Statistics

For all statistical analyses, confirm that the following items are present in the figure legend, table legend, main text, or Methods section.

n/a	Confirmed	
☐	☒	The exact sample size (*n*) for each experimental group/condition, given as a discrete number and unit of measurement
☐	☒	A statement on whether measurements were taken from distinct samples or whether the same sample was measured repeatedly
☐	☒	The statistical test(s) used AND whether they are one- or two-sided<br>*Only common tests should be described solely by name; describe more complex techniques in the Methods section.*
☐	☒	A description of all covariates tested
☐	☒	A description of any assumptions or corrections, such as tests of normality and adjustment for multiple comparisons
☐	☒	A full description of the statistical parameters including central tendency (e.g. means) or other basic estimates (e.g. regression coefficient) AND variation (e.g. standard deviation) or associated estimates of uncertainty (e.g. confidence intervals)
☐	☒	For null hypothesis testing, the test statistic (e.g. *F*, *t*, *r*) with confidence intervals, effect sizes, degrees of freedom and *P* value noted<br>*Give P values as exact values whenever suitable.*
☒	☐	For Bayesian analysis, information on the choice of priors and Markov chain Monte Carlo settings
☒	☐	For hierarchical and complex designs, identification of the appropriate level for tests and full reporting of outcomes
☒	☐	Estimates of effect sizes (e.g. Cohen's *d*, Pearson's *r*), indicating how they were calculated

Our web collection on statistics for biologists contains articles on many of the points above.

Software and code

Policy information about availability of computer code

Data collection

The following standard software were used to analyze whole genome copy number profiling and sequencing data. References are given in Online Methods:

CytoScan HD SNP array (Thermo Fisher/Affymetrix) was used and the copy number profiles were visualized with Chromosome Analysis Suite (version 3.3.0.139; Thermo Fisher/Affymetrix) to analyze DNA from fresh frozen samples and cell culture experiments. CEL files were normalized by the R package Rawcopy, and scatter plots whose x-axis and y-axis denotes log2 ratio and allelic imbalance, respectively, were generated through the R package TAPS (Tumor Aberration Prediction Suite). CEL-files were converted to OSCHP-files with the OncoScan Console (version 1.3.0.39; Affymetrix). The Nexus Express software for Oncoscan (version 3.1) was used for visual inspection of the OSCHP-files. Aberration plots were generated with BioCircos (v0.3.4) in R (v4.1).

For patient derived xenograft analyses, paired end reads were mapped to the human reference genome (GRCh37 with decoys from the 1000 Genomes' Project) using BWA-MEM 0.7.1513. Duplicate reads were marked using sambamba 0.6.714. Somatic variant calling was performed using freebayes (with the –pooled-continuous, --pooled-discrete and -F 0.03 flags; https://arxiv.org/abs/1207.3907v2) and strelka2 (using the --exome flag). The Polyphen-2 tool, was used to predict the impact of mutations on protein function.

For WES of FFPE tumor material paired-end reads were aligned to the human reference genome hg19 by BWA-MEM (https://arxiv.org/abs/1303.3997v2). Duplicate reads marking and local realignment were performed by GATK (version 4.0.11.0)18. Mutect (version 1.1.7), GATK Mutect2 (version 4.0.11.0), and MuSE (version v1.0rc) were used to identify somatic single nucleotide variants (SNVs) and small insertions/deletions (indels). The SNVs and indels called by Mutect2 were further filtered with the GATK FilterMutectCalls. The variant vcf files were converted into maf files by the vcf2maf package (https://github.com/mskcc/vcf2maf).

Data analysis	The in-house software DEVOLUTION was used for clustering and phylogenetic analyses of bulk sequencing data . The full software including access links is published in Andersson et al. Commun Biol 4,1103 (2021). After clustering phylogenetic trees were constructed using standard R packages phangorn (v2.8.1) and visualized using the ggplot2 (v.3.3.5) package. Another in-house software was used to construct phylogenies from single cell WGS and this is published online at https://github.com/NatalieKAndersson/SCEM. Subclonal deconvolution was benchmarked against DeCiFer (v2.1.4).<br>See also separate code and software submission checklist.

For manuscripts utilizing custom algorithms or software that are central to the research but not yet described in published literature, software must be made available to editors and reviewers. We strongly encourage code deposition in a community repository (e.g. GitHub). See the Nature Portfolio guidelines for submitting code & software for further information.

Data

Policy information about availability of data

All manuscripts must include a data availability statement. This statement should provide the following information, where applicable:

- Accession codes, unique identifiers, or web links for publicly available datasets
- A description of any restrictions on data availability
- For clinical datasets or third party data, please ensure that the statement adheres to our policy

The datasets generated or analysed during the current study are available in curated raw data format in Table S2. Raw data plots from whole genotyping of clinical samples, PDX tumors and cell lines are available at Zenodo (https://doi.org/10.5281/zenodo.8017767) . Raw data from whole genome genotyping/sequencing have been doposited the the European Genome-phenome Archive (https://ega-archive.org) under accession number EGAS00001007650 and dataser ID: EGAD00001015012, EGAD00001015413, and EGAD00001015414. Access to raw genomic data will require assessment by a data access committee of the applicant's ethics permit as well as technical specifications of how the data should be stored to ensure compliance with ethics regulations of the study as well as GDPR. A data transfer agreement will have to made and signed by legal representatives.

Research involving human participants, their data, or biological material

Policy information about studies with human participants or human data. See also policy information about sex, gender (identity/presentation), and sexual orientation and race, ethnicity and racism.

Reporting on sex and gender	No specific analyses of sex and gender were performed as this was not within the scope of the study. Patients were enrolled based on the diagnosis and the availability of biological samples. The cohort contains children of both sexes as expected because the disease under study affects both sexes almost equally There is a slight increased risk for boys and there is also a slight male predominance in our small study cohort. Sex was assigned and not self-reported as most of the study participants were young children. The study cohort is too small for meaningful subdivision according to sex and specifying it may threaten privacy of participant.
Reporting on race, ethnicity, or other socially relevant groupings	No such parameters were reported. Albeit parental socioeconomic status may impact childhood cancer survival, our sample is too small for any survival analysis and it was beyond the scope of the study to analyze survival parameters.
Population characteristics	Children diagnosed with cancer in Sweden. None of the patients had earlier disease that could modify their outcome. Genetic characteristics of tumors are amply provided in the manuscript and clinical parameters in Supplementary Table S1a.
Recruitment	Patients selected from a 20-year consecutive regional cohort based on availability of tumor material for analysis.
Ethics oversight	The study was approved by the regional ethics review board under permit numbers L289-11 (genomic analyses; updated as L796-2017) and L605-05 (biobanking; updated as L883-2018). For data sharing, this was complemented by permit from the Swedish Ethical Review Authority (2023-01550-01).

Note that full information on the approval of the study protocol must also be provided in the manuscript.

Field-specific reporting

Please select the one below that is the best fit for your research. If you are not sure, read the appropriate sections before making your selection.

☒ Life sciences ☐ Behavioural & social sciences ☐ Ecological, evolutionary & environmental sciences

For a reference copy of the document with all sections, see nature.com/documents/nr-reporting-summary-flat.pdf

Life sciences study design

All studies must disclose on these points even when the disclosure is negative.

Sample size	This is an explorative study where all patients (n=12) from a consecutive cohort were enrolled based on availability of material for analysis.
Data exclusions	Patients having scarce material in biobanks/pathology archives were excluded, creating a bias towards patients with disease remaining after treatment. However, these patients are the ones most relevant from a clinical perspective and they were the ones that the study was in facto focused on.

Replication	Because the present study would take many years to reproduce in a clinical prospective study, we chose to use patient-derived xenografts (PDXs) as well as cell cultures to evaluate whether the data could be reproduced in prospective, controlled setting.
Randomization	This is an explorative study where all patients (n=12) from a consecutive cohort of 20 years were enrolled based on availability of material for analysis. No statistical method was used to predetermine sample size. No data were excluded from the analyses. Apart from assignment of animals to treatment or control arms, the experiments were not randomized.
Blinding	The investigators were not blinded to allocation during experiments and outcome assessment.

Reporting for specific materials, systems and methods

We require information from authors about some types of materials, experimental systems and methods used in many studies. Here, indicate whether each material, system or method listed is relevant to your study. If you are not sure if a list item applies to your research, read the appropriate section before selecting a response.

Materials & experimental systems

n/a	Involved in the study
☒	☐ Antibodies
☐	☒ Eukaryotic cell lines
☒	☐ Palaeontology and archaeology
☐	☒ Animals and other organisms
☐	☒ Clinical data
☒	☐ Dual use research of concern
☒	☐ Plants

Methods

n/a	Involved in the study
☒	☐ ChIP-seq
☒	☐ Flow cytometry
☒	☐ MRI-based neuroimaging

Eukaryotic cell lines

Policy information about cell lines and Sex and Gender in Research

Cell line source(s)	The IMR-32 (CCL-127) and SK-N-SH (HTB-11) neuroblastoma cell lines were from ATCC
Authentication	SNP array
Mycoplasma contamination	Tested negative.
Commonly misidentified lines (See ICLAC register)	Not present in ICLAC at search conducted Febr 16 2023

Animals and other research organisms

Policy information about studies involving animals; ARRIVE guidelines recommended for reporting animal research, and Sex and Gender in Research

Laboratory animals	NMRI nude mice were purchased from Taconic.
Wild animals	Not applicable.
Reporting on sex	Not collected or reported as data were obtained solely on tumor cells implanted to animals, not on the animal physiological response to tumors.
Field-collected samples	Not applicable.
Ethics oversight	All procedures were conducted according to the guidelines from the regional Ethics Committee for Animal Research (permit no. M11-15, 19012-19 and 289-2011 with update 2017).

Note that full information on the approval of the study protocol must also be provided in the manuscript.

Clinical data

Policy information about clinical studies

All manuscripts should comply with the ICMJE guidelines for publication of clinical research and a completed CONSORT checklist must be included with all submissions.

Clinical trial registration	This was not clinical trial.
Study protocol	This was not clinical trial.

Data collection	We reviewed pathology files of all patients <18 years of age diagnosed with and treated for a histopathologically verified neuroblastoma in the Southern Healthcare Region of Sweden from 1998-2018. From this cohort of 50 patients, we selected those from whom there were samples with >50% viable tumor cells available for DNA-analysis, material from at least two different disease time points, and samples from at least two intratumoral locations from at least one of these time points. This resulted in totally 12 patients.
Outcomes	This was not clinical trial.

Plants

Seed stocks	Not applicable.
Novel plant genotypes	Not applicable.
Authentication	Not applicable.
